# Maize-based polyculture, not monoculture, sustained precolonial societies in the Brazilian Cerrado

**DOI:** 10.1126/sciadv.aef7066

**Published:** 2026-07-15

**Authors:** Eliane Chim, Henry L. A. Fernandes, Sibeli A. Viana, Haruan Straioto, Elver Mayer, Sophia Huesges, Erin Scott, Diego T. Mendes, Danilo R. Zardo, Nicolás M. Stríkis, Gilmar Henriques, Rafael L. de Souza, Maria Ana Correia, Luana Caroline Nicolau, Erica de S. Rocha, Edward Koole, Julio C. Rodrigues, Talita Lima, Andersen Liryo, Andrei Isnardis, Lucas Bueno, Juvandi de S. Santos, Carlos Etchevarne, Marcia A. Alves, André Prous, Axel Steinhof, Francisco William da Cruz Júnior, Jana Ilgner, Ricardo Fernandes, Patrick Roberts, André Strauss

**Affiliations:** ^1^ Museu de Arqueologia e Etnologia, Universidade de São Paulo, São Paulo, Brazil.; ^2^ Department of Coevolution of Land Use and Urbanisation, Max Planck Institute of Geoanthropology, Jena, Germany.; ^3^ Universidade Federal do Recôncavo da Bahia, Cachoeira, Brazil.; ^4^ Pontifícia Universidade Católica de Goiás, Goiânia, Brazil.; ^5^ Universidade Federal do Vale do São Francisco, Senhor do Bonfim, Brazil.; ^6^ Laboratory Central Unit, Max Planck Institute of Geoanthropology, Jena, Germany.; ^7^ Museu Antropológico, Universidade Federal de Goiás, Goiânia, Brazil.; ^8^ Instituto de Geociências, Universidade de São Paulo, São Paulo, Brazil.; ^9^ Centro Interdisciplinar de Arqueologia e Evolução do Comportamento Humano, Universidade do Algarve, Faro, Portugal.; ^10^ Institut für Geowissenschaften, Johannes Gutenberg-Universität Mainz, Mainz, Germany.; ^11^ Museu Nacional, Universidade Federal do Rio de Janeiro, Rio de Janeiro, Brazil.; ^12^ Universidade Federal de Minas Gerais, Belo Horizonte, Brazil.; ^13^ Universidade Federal de Santa Catarina, Florianópolis, Brazil.; ^14^ Universidade Estadual da Paraíba, Campina Grande, Brazil.; ^15^ Universidade Federal da Bahia, Salvador, Brazil.; ^16^ Max Planck Institute for Biogeochemistry, Jena, Germany.; ^17^ Department of Archaeology, Max Planck Institute of Geoanthropology, Jena, Germany.; ^18^ Faculty of Archaeology, University of Warsaw, Warsaw, Poland.; ^19^ Climate Change and History Research Initiative, Princeton University, Princeton, NJ, USA.

## Abstract

Maize has long been argued to have played a key role in the transition to food production in the Americas. Recent work has even suggested that this included “monoculture” in parts of South America. Here, we report stable isotope data (δ^13^C, δ^15^N, and δ^18^O) of 101 human individuals from 37 late Holocene archaeological sites across the Cerrado (Brazilian savanna), Caatinga (dry forest), and Atlantic Forest biomes of central-eastern South America, in addition to present faunal baseline and radiocarbon dates. The results show cultural and economic diversity among archaeological traditions. Integrating archaeological, palaeoecological, and chronological data, we argue for the coexistence of a diversified and a more complex food production systems in the Cerrado biome, comparable to those of precolonial urban-scale populations practicing maize agriculture in the Amazon. These findings demonstrated that eastern South America populations practiced maize-based polyculture, reshaping current models of cultural and environmental dynamics in the Cerrado biome.

## Teaser

Evidence from human bones highlights complexity of ancient food production among precolonial societies in eastern South America.

## INTRODUCTION

The domestication of plants and the reliance on food production systems have been seen as crucial thresholds in human socioeconomic organization and environmental interactions (*1*, *2*). The movement of maize (*Zea mays*) following its domestication in Mexico c. 9000 calibrated years before the present (cal. B.P.) (*3*) has particularly frequently been associated with the emergence of socio-complexity in different parts of the Americas (*4*, *5*). While some studies have explored the idea of intensive farming systems (*5*), other research in South America, particularly in other areas of the Amazon Basin, has highlighted varied food production relationships, including diversified systems based on domesticated and wild plants, as well as the practice of polyculture (mixed cropping) (*6*–*10*). While the Amazon has played a critical role in reevaluations of the emergence of food production, social complexity, and environmental impacts in tropical contexts (*11*–*13*), other, highly biodiverse regions in South American have been relatively neglected. In the Cerrado (tropical savanna-like) biome of central-eastern South America, the sudden appearance of large villages after 1200 cal. B.P. represented a clear sociocultural change and has previously been associated with agricultural societies and a heavy reliance on domesticated plants, particularly maize (*14*–*17*). Macrobotanical remains show that maize was present in this region by at least 3700 cal. B.P. (*18*), and genomic analysis of maize dated to c. 650 cal. B.P. shows strong genetic links to Andean crops, suggesting a possible second west-to-east expansion of maize traditions, which could be associated with the emergence of large villages (*6*). Nevertheless, there is now a lack of direct evidence regarding the diet of these societies, leaving a substantial gap in understanding their overall economic organization, subsistence practices, and interactions with the environment.

Although the Cerrado biome was traditionally considered a resource-limited environment due to its low primary productivity, interannual dry periods and frequent fires (*19*), it has increasingly been recognized as a biodiversity hotspot (*20*) and an environment influenced by early management practices of small-scale societies (*21*). The biodiversity of this biome is now under serious threat from agricultural commodity expansion, droughts driven by global warming, and the lack of policies to protect nonforest ecosystems (*22*, *23*). Moreover, the archaeological record indicates considerable cultural diversity during the Holocene (*24*). Human occupations are detected mainly in rock shelters, where the first pottery remains appear around 3000 cal. B.P., the Una ceramic tradition (*25*, *26*). Major sociocultural changes and population growth are represented by the emergence and rapid expansion of open-air villages associated with the Aratu ceramic tradition between 1200 and 500 cal. B.P., with populations ranging from hundreds to two thousand people per village (*27*). The mechanism behind the appearance and expansion of the Una and Aratu tradition remains uncertain and may reflect local innovations, interactions with other populations, or a combination of both (*14*, *28*, *29*). Since cultivated plants predate pottery in the region, populations associated with these ceramic traditions have been linked to a horticultural diet, especially those of the Aratu ceramic tradition, which are thought to have possibly relied on domesticated plants (*14*, *15*). However, the relationship between maize consumption and the expansion of these large open-air villages remains a working hypothesis.

**Fig. 1.**
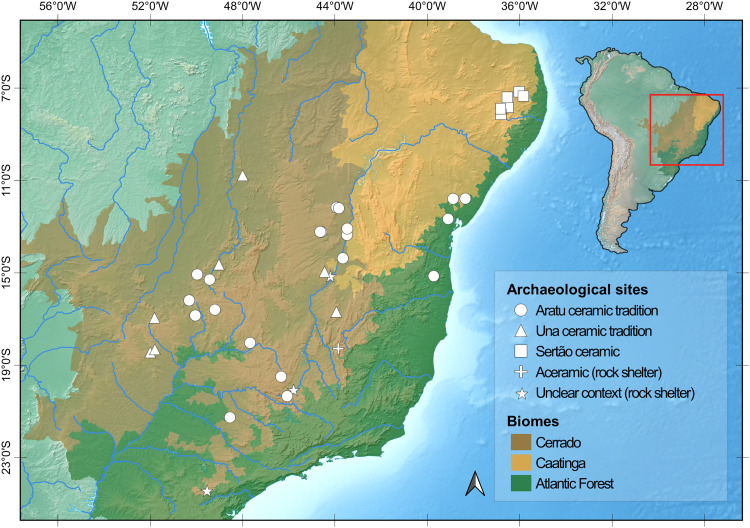
Location of the study area, biomes, and archaeological sites from late Holocene. Created for this study using QGIS 3.32.0 and Natural Earth.

Here, we present direct isotopic dietary evidence from late Holocene populations associated with large open-air villages of the Cerrado biome, along with new isotopic data from rock shelters and new radiocarbon dates. These open-air villages are contemporary with, and share the same circular pattern as, those of the Upper Xingu (*30*), where large-scale anthropogenic transformations of Amazonian landscapes and vast urban networks have been identified (*31*). Our analytical framework encompasses late Holocene populations from central-eastern South America, including Aratu tradition villages in the Cerrado and Atlantic Forest biomes, rock shelters attributed to the Una tradition and one lacking ceramic in the Cerrado biome, and sites associated with sertão pottery in the semiarid Caatinga biome (text S1). We analyze stable carbon (δ^13^C), nitrogen (δ^15^N), and oxygen (δ^18^O) isotopes from bone collagen and dental enamel of 101 human individuals from 37 archaeological sites (Fig. 1 and text S2), reflecting a diverse cultural spectrum throughout the last millennium. Furthermore, to obtain reference values, we also analyze faunal remains from five archaeological sites from the same period and region (text S2). δ^13^C, δ^15^N, and δ^18^O analyses provide direct insights into reliance on C_3_ or C_4_ resources, trophic level and aquatic resources use, and hydrology (text S3). Together, our sample set provides a key opportunity to explore economic organization, subsistence practices, and interactions with the environment of the societies of the large open-air villages (Aratu), comparing them with the producers of the pottery found in rock shelters (Una) and other contemporaneous populations.

## RESULTS

### Chronology

Archaeological, stratigraphic, and chronological information are presented in detail in Supplementary Text and data S1, allowing the contextualization of the samples. Bayesian chronological modeling of radiocarbon dates indicates that the samples analyzed from the Aratu tradition open-air villages (1043 to 333 cal. B.P.) and Una tradition (922 to 465 cal. B.P.) are partially contemporary, as well as remains associated with sertão ceramic and without cultural classifications (1055 to 526 cal. B.P.) (figs. S35 to S38 and data S1). Therefore, overall, our samples come from a period dated between 1055 and 333 cal. B.P.

### Stable isotope analysis of collagen

**Fig. 2.**
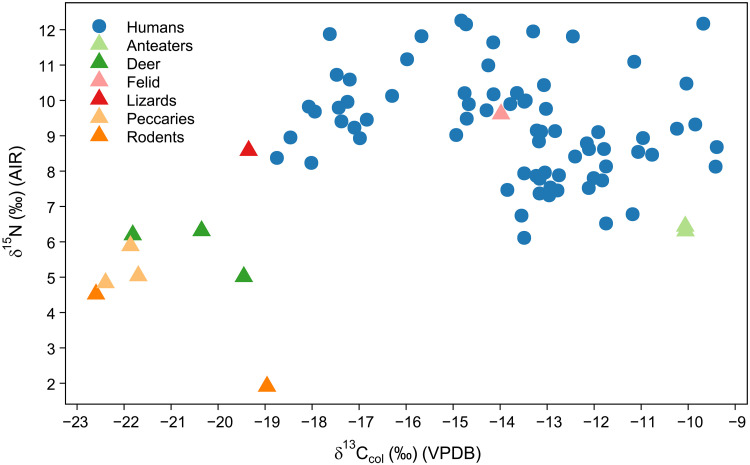
Human and fauna bone collagen δ^13^C and δ^15^N values (data S2). The samples date between 1055 and 333 cal. B.P. VPDB, Vienna Pee Dee belemnite.

Collagen preservation was considered suitable when samples presented collagen yield >1% and C:N ratios between 2.9 and 3.5 (*32*). We processed 124 samples, of which 109 had collagen yield >1% and 85 presented C:N ratios between 2.9 and 3.5. We obtained collagen results from 12 faunal samples and from the bones or dentine of 73 human individuals (Fig. 2 and data S2). Two individuals were excluded from the final analysis to avoid potential effects of weaning, as the samples came from the dentine of a deciduous tooth and a subadult long bone.

All faunal samples are from sites in the Cerrado. The herbivorous and omnivorous groups, comprising deer (*n* = 3), lizards (*n* = 1), rodents (*n* = 2), and peccaries (*n* = 3), have δ^13^C_col_ values ranges of −22.6 to −18.9 per mil (‰) (mean = −20.9 ± 1.4‰), while the carnivore, a feline (*n* = 1), exhibits a δ^13^C_col_ value of −14.0‰, and myrmecophagous, represented by anteaters (*n* = 2), displays δ^13^C_col_ values of −10.1‰ (Fig. 2). δ^15^N values among the herbivorous, omnivorous, and myrmecophagous range from 4.5 to 6.4‰ (mean = 5.6 ± 0.8‰). The exceptions are one rodent with a lower δ^15^N value of 1.9‰ and the lizard with a higher value of 8.6‰, which is close to the feline’s δ^15^N value of 9.6‰ (Fig. 2). A Kruskal-Wallis tests show no statistically significant differences in δ^13^C (chi-square = 6.5, df = 3, *P* > 0.05) (table S1) and δ^15^N (chi-square = 4.8462, df = 3, *P* > 0.05) (table S2) distributions between herbivorous, omnivorous, carnivorous, and myrmecophagous animals.

**Fig. 3.**
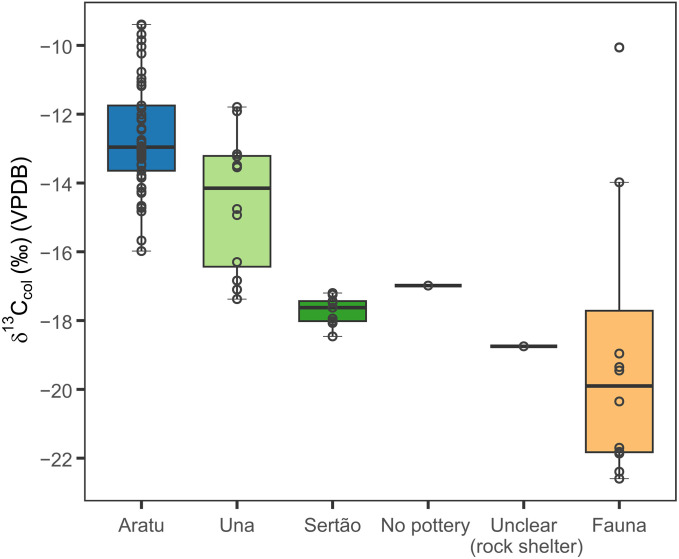
Human and fauna bone collagen δ^13^C values (data S2). Human individuals are shown according to their cultural and contextual affiliations: Aratu tradition, Una tradition, pottery makers from rock shelters in the Caatinga biome, individuals from rock shelters without pottery, and an individual from rock shelter with unclear cultural context. The samples date between 1055 and 333 cal. B.P. Boxes shows the median and the lower (25%) and upper (75%) quartiles, whiskers extend to 1.5 × interquartile range (IQR), and black dots represent outliers.

The δ^13^C_col_ values of humans vary widely, ranging from −18.7 to −9.4‰ (mean = −13.8 ± 2.4‰) (Figs. 2 and 3). Individuals from the Caatinga rock shelters associated with sertão pottery (*n* = 9, δ^13^C_col_ mean = −17.7 ± 0.4‰), as well as an individual from rock shelter not associated with pottery (*n* = 1, δ^13^C_col_ = −16.9‰), had lower δ^13^C_col_ than individuals from Una (*n* = 12, δ^13^C_col_ mean = −14.5 ± 1.9‰) and Aratu traditions (*n* = 49, δ^13^C_col_ mean = −12.7 ± 1.6‰). Welch’s analysis of variance (ANOVA) indicated a significant difference in δ^13^C values among Aratu, Una, and sertão individuals [*F*_(2,25.03)_ = 176.24, *P* < 0.001] (tables S3 to S5). Post hoc Games-Howell comparison tests indicated significant differences between Una and Aratu tradition individuals (*P* = 0.022) and between Aratu and Caatinga individuals (*P* = 0) and Una and sertão individuals (*P* = 0.0004) (table S6).

**Fig. 4.**
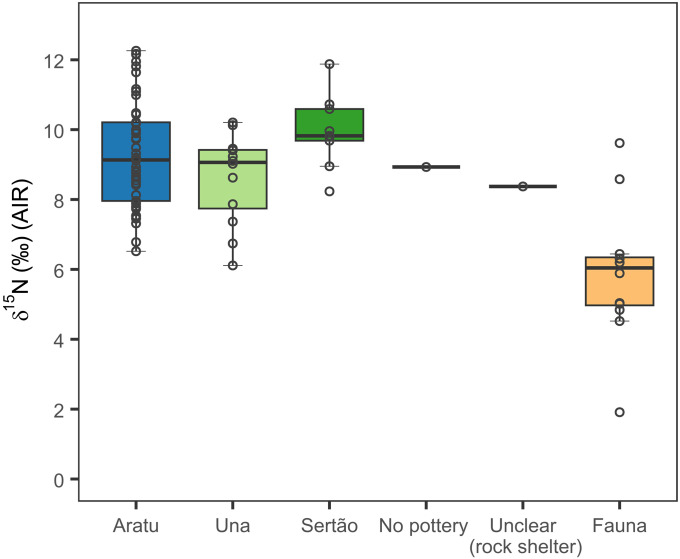
Human and fauna bone collagen δ^15^N values (data S2). Human individuals are shown according to their cultural and contextual affiliations: Aratu tradition, Una tradition, pottery makers from rock shelters in the Caatinga biome, individuals from rock shelters without pottery, and an individual from rock shelter with unclear cultural context. The samples date between 1055 and 333 cal. B.P. Boxes shows the median and the lower (25%) and upper (75%) quartiles, whiskers extend to 1.5 × IQR, and black dots represent outliers.

The human δ^15^N values range from 6.1 to 12.3‰, showing similar results for the different groups (Fig. 4). Individuals from the Una tradition (*n* = 12, δ^15^N mean = 8.6 ± 1.3‰), and an individual from rock shelter not associated with pottery (*n* = 1, δ^15^N = 8.93‰), appear to have slightly lower δ^15^N than individuals from and Aratu tradition (*n* = 49, δ^15^N mean = 9.3 ± 1.6‰) and Caatinga rock shelters associated with sertão pottery (*n* = 9, δ^15^N mean = 10.0 ± 1.5‰). However, statistical comparisons indicated no significant differences in δ^15^N values among these groups [ANOVA: *F*(_2,67_) = 2.252, *P* = 0.113] (tables S3, S7, and S8). Although δ^15^N varies in different environments (*33*), here, human δ^15^N values from the Cerrado (*n* = 59, δ^15^N mean = 8.97 ± 1.3‰) and Caatinga (*n* = 9, δ^15^N mean = 9.9 ± 1.0‰) biomes showed no significant differences (Tukey post hoc pairwise comparisons, *P* = 0.093), while values from individuals from both biomes showed significant differences with individuals from Atlantic Forest (*n* = 4, δ^15^N mean = 11.9 ± 0.5‰) (Tukey post hoc pairwise comparisons, *P* < 0.05) (tables S9 to S12).

### Stable isotope analysis of tooth enamel

**Fig. 5.**
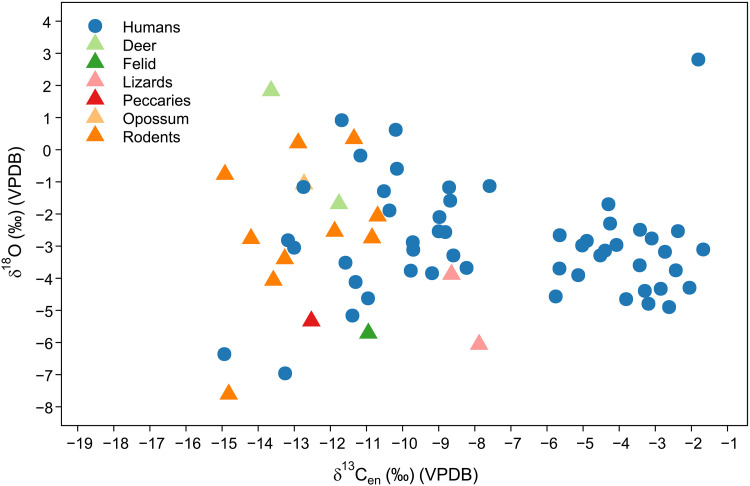
Human and fauna bulk tooth enamel δ^13^C and δ^18^O values (data S2). The samples date between 1055 and 333 cal. B.P.

We obtained the results of stable carbon and oxygen isotopes from the enamel of 17 faunal tooth samples and 52 human individuals (Fig. 5 and data S2). The faunal samples comprise mammals and reptiles from the Cerrado and presented δ^13^C_en_ with a fairly large range (−14.9 to −7.9‰). The mammal group, including feline (*n* = 1), deer (*n* = 2), opossum (*n* = 1), peccary (*n* = 1), and rodents (*n* = 10), has δ^13^C_en_ values ranging from −14.9 to −10.7‰ (mean = −12.7 ± 1.4‰), while the lizards (*n* = 2) have δ^13^C_en_ values ranging from −8.7 to −7.9‰ (mean = −8.3 ± 0.5‰). A Kruskal-Wallis test shows statistically significant differences (chi-square = 5, df = 2, *P* < 0.05) between mammals and reptiles, with post hoc analysis corroborating this (pairwise Wilcoxon = 0.015). The δ^18^O values from fauna also showed a large range, from −7.6 to 1.8‰ (mean = −2.8 ± 2.5‰). However, mammals (*n* = 15, δ^18^O mean = −2.5 ± 2.5‰) and reptiles (*n* = 2, δ^18^O mean = −5 ± 1.5‰) showed no significant differences in δ^18^O values (Kruskal-Wallis test, chi-square = 2.2222, df = 1, *P* > 0.05) (table S14). Considering the diet of animals, the herbivorous group includes just deer (*n* = 2), with δ^13^C_en_ values ranges of −13.6 to −11.8‰, while the omnivorous group, comprising lizards (*n* = 2), opossum (*n* = 1), rodents (*n* = 10), and peccary (*n* = 1), has δ^13^C_en_ values ranges of −14.9 to −7.9‰ (mean = −12.2 ± 2.1‰), and the carnivore, a feline (*n* = 1), exhibits δ^13^C_en_ value of −11.0‰. δ^18^O values among the herbivorous, omnivorous, and carnivorous range from −7.6 to 1.8‰ (mean = −2.8 ± 2.5‰). A Kruskal-Wallis test shows no statistically significant differences in δ^13^C_en_ (chi-square = 0.80672, df = 2, *P* > 0.05) and δ^18^O (chi-square = 3.8543, df = 2, *P* > 0.05) distributions between herbivorous, omnivorous, and carnivorous animals.

**Fig. 6.**
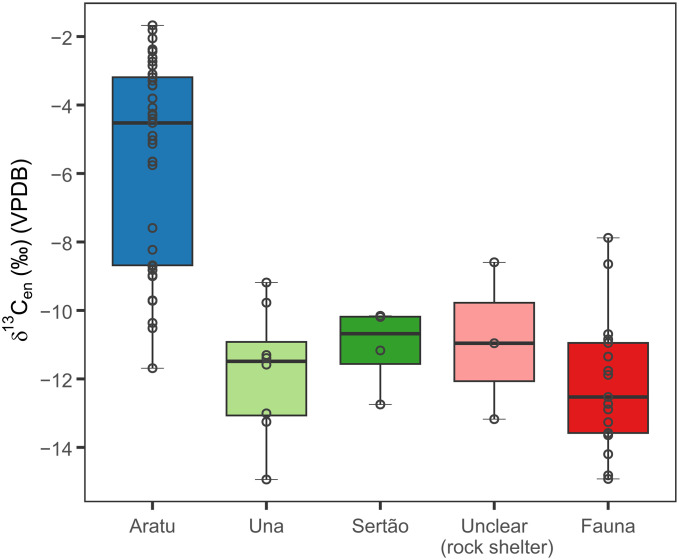
Human and fauna bulk tooth enamel δ^13^C values (data S2). Human individuals are shown according to their cultural and contextual affiliations: Aratu tradition, Una tradition, pottery makers from rock shelters in the Caatinga biome, and individual from rock shelter with unclear cultural context. The samples date between 1055 and 333 cal. B.P. Boxes shows the median and the lower (25%) and upper (75%) quartiles, and whiskers extend to 1.5 × IQR.

**Fig. 7.**
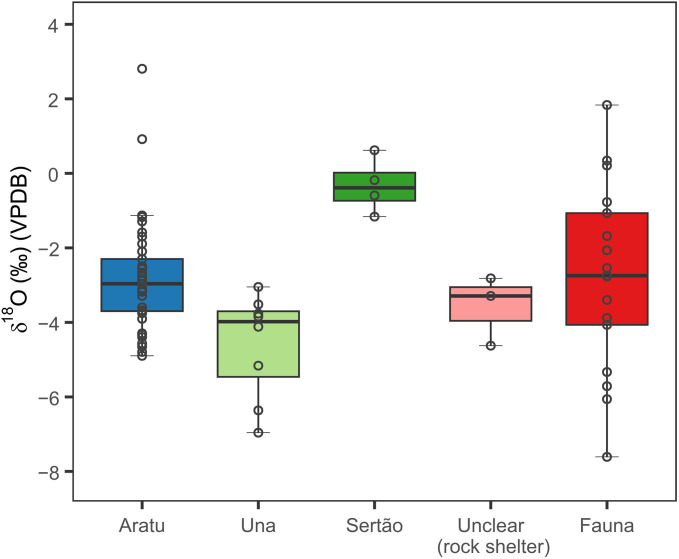
Human and fauna bulk tooth enamel δ^18^O values (data S2). Human individuals are shown according to their cultural and contextual affiliations: Aratu tradition, Una tradition, pottery makers from rock shelters in the Caatinga biome, and individual from rock shelter with unclear cultural context. The samples date between 1055 and 333 cal. B.P. Boxes shows the median and the lower (25%) and upper (75%) quartiles, and whiskers extend to 1.5 × IQR.

Human δ^13^C_en_ values have a larger range than the fauna, from −14.9 to −1.7‰, as well as δ^18^O values, ranging from −7.0 to 2.8‰ (Figs. 5 to 7 and data S2). Overall, individuals from rock shelters (*n* = 15, δ^13^C_en_ mean = −11.4 ± 1.7‰) had lower δ^13^C_en_ than individuals from the open-air villages (Aratu tradition) (*n* = 37, δ^13^C_en_ mean = −5.5 ± 2.9‰) (Fig. 6). Considering the cultural diversity of the sample set, individuals from the Una tradition (*n* = 8, δ^13^C_en_ mean = −11.8 ± 1.9‰), Caatinga rock shelters (*n* = 4, δ^13^C_en_ mean = −11.1 ± 1.2‰), and rock shelter with unclear cultural context (*n* = 3, δ^13^C_en_ mean = −10.9 ± 2.3‰) had lower δ^13^C_en_ than individuals from the Aratu tradition (*n* = 37, δ^13^C_en_ mean = −5.5 ± 2.9‰) (Fig. 6). In Aratu sites, however, δ^13^C_en_ values clearly represent two different dietary groups (Figs. 5 and 6), even between contemporary individuals from the same archaeological site. The Shapiro-Wilk test indicated nonnormal distribution for δ^13^C_en_ from Aratu tradition individuals (*P* < 0.05), whereas data from rock shelter individuals did not deviate from normality (*P* > 0.05) (table S13), because of that, a Kruskal-Wallis test was performed, although a Levene’s test indicated equal variances (*P* = 0.2028) (table S14). A Kruskal-Wallis test indicated a highly significant difference (chi-square = 25.783, df = 3, *P* < 0.001) between δ^13^C_en_ of humans from Aratu and Una traditions, Caatinga rock shelters, and rock shelters with unclear cultural context (table S15). Post hoc Wilcoxon comparison tests indicated no significant differences of δ^13^C_en_ between individuals from rock shelters, which include those associated with the Una tradition, Caatinga, and unclear cultural context (*P* > 0.05). Nevertheless, the post hoc tests indicated significant differences in δ^13^C_en_ between Aratu tradition and individuals from Caatinga rock shelters and rock shelters with unclear cultural contexts (*P* < 0.05) and in δ^13^C_en_ between Aratu and Una individuals (*P* < 0.001) (table S16).

## DISCUSSION

This study examines subsistence strategies in the precolonial Brazilian Cerrado based on isotopic and chronological evidence. Our stable isotope data are consistent with the hypothesis that food production may have played a key role in the expansion of the population from Aratu ceramic tradition villages, as the δ^13^C of bone collagen and dental enamel from the earliest directly dated individuals of this tradition were more ^13^C enriched, indicating clear use of C_4_ plant–based resources (i.e., maize). At the same time, our data show that the contemporaneous producers of the Una and Aratu ceramic traditions had markedly different diets, supporting the hypothesis that they were culturally distinct. In open-air sites, the combined evidence from our isotopic data, the presence of starch grains of maize and, probably, sweet potato (cf. *Ipomoea batatas*) (*34*), along with wear marks consistent with fermentation of alcoholic beverages in Aratu vessels (*35*), points to a broader social and dietary role for cultivated plants within village life. These findings suggest that the Aratu expansion was accompanied by food production, with maize not only serving as a dietary staple but also as a key element in food processing and possibly communal or ritual practices. However, it is still difficult to assess whether the Una tradition represents a homogeneous cultural unit, since stable isotope results indicate considerable dietary variation among individuals. Collagen δ^13^C primarily reflects the protein portion of the diet (*36*), and our data suggest intragroup variability, with individuals from rock shelters exhibiting different degrees of C_3_ and C_4_ resource consumption compared with the faunal baseline, which tends toward a C_3_-based diet. In contrast, δ^13^C_en_ values represent the whole diet (*37*) and are particularly sensitive to reflect plant consumption. Tooth enamel from individuals from rock shelters was more ^13^C depleted, consistent with the consumption of C_3_ plants, as do the faunal baseline. Overall, our results indicate the coexistence of small-scale horticulture and a more complex system of food management, embedded in both subsistence and social interaction across the Cerrado ecosystems.

Stable isotope measurements of tree leaves across Brazilian biomes show that precipitation and canopy effect further influence δ^15^N and δ^13^C values (*33*). Cerrado plants typically display mean δ^15^N values of −0.3 ± 2.3‰, evergreen forests show higher values of 4.3 ± 2.5‰, and Caatinga plants present even higher values of 9.3 ± 3.7‰, likely due to low leaching and plant uptake (*33*). For δ^13^C, Caatinga plants exhibit the highest mean values (−26.7 ± 1.6‰), followed by Cerrado plants (−28.9 ± 1.6‰) and evergreen forests (−31.8 ± 2.1‰), probably due to the canopy effect (*33*). Consequently, individuals from the Caatinga are expected to be more enriched in ^15^N and ^13^C, mirroring the values observed in local flora. This expectation is corroborated by bone collagen data from deer, a herbivore sampled in both the Cerrado and Caatinga biomes (*38*, *39*). Our data from the Cerrado show that deer (*n* = 3) display average values of δ^15^N and δ^13^C_col_ of 5.8 ± 0.7‰ and −20.5 ± 1.2‰, respectively (Fig. 2), consistent with published data from the same biome, where deer present average δ^15^N values of 5.1 ± 0.9‰ and δ^13^C_col_ values of −21.8 ± 1.0‰ (*n* = 16) (*38*). Deer from the Caatinga biome, however, display extremely high δ^15^N values, reaching 17.2‰, and δ^13^C_col_ of −18.4‰ (*n* = 1) (*39*), emphasizing the potential impacts of aridity on nitrogen enrichment. Meanwhile, our data from humans δ^15^N show no significant differences between individuals from these biomes, suggesting a lower intake of animal protein among the Caatinga population, despite the elevated δ^15^N values in the local ecosystem. The lack of faunal baseline limits interpretations of human diet in the Atlantic Forest; however, on the basis of the δ^15^N from flora (*33*), the analyzed individuals may have had a higher contribution of meat in their diet compared with individuals from the other biomes. For δ^13^C of bone collagen and dental enamel, our data indicate a lower intake of C_4_ resources among Caatinga individuals, while humans from the Cerrado exhibit a broad range of δ^13^C values that overlap with those from other biomes. This variability suggests significant cultural and economic diversity among Cerrado societies, highlighting different strategies of interactions with the environment and diets shaped more by cultural choices than ecological limitations.

Early 20th century anthropological accounts described the societies of central-eastern South America as predominantly medium to highly mobile foragers with low population densities (*40*), despite the presence of large archaeological settlements. Horticulture was considered secondary, largely confined to fertile riverbanks, and insufficient to support permanent sedentary populations (*41*). Instead, in the 20th century, these societies used a mixed strategy, dispersing into small hunting and gathering groups for most of the year and returning to their villages during harvest time (*40*, *41*). Ecological factors, such as soil quality and climate conditions, were assumed to be the key determinants of this subsistence pattern (*40*). However, this perspective was based largely on historical descriptions, not considering the impact of colonialism on the demographics and lifeways of these societies (*42*). Archaeological evidence shows diverse settlement patterns, with thousands of occupants (*27*). Dried plant remains have demonstrated that crops were cultivated in the Cerrado at least by 4300 cal. B.P., with plant macroremains appearing systematically in the archaeological record after 2000 cal. B.P. (*43*). From this period onward, the archaeobotanical record includes evidence of maize, manioc (cf. *Manihot esculenta*), bottle gourd (*Lagenaria siceraria*), beans (*Phaseolus lunatus* and *Phaseolus vulgaris*), cotton (cf. *Gossypium barbadense*), peanuts (*Arachis hypogaea*), and squash (*Cucurbita* spp.), as well as several species of wild plants (*18*, *43*, *44*). Yet until now, little was known about their actual dietary reliance on different resources across space and time. Our isotopic results begin to fill this gap. The diversity of plant remains, coupled with the variability in stable isotope data from rock shelter individuals, suggests a diversified system of food production. Conversely, isotopic values from individuals associated with the Aratu ceramic tradition indicate heavier reliance on maize, pointing to agriculture as a central element in the Aratu villages. Collectively, these results provide new chronological and dietary evidence for food production in eastern South America, challenge earlier characterizations of Cerrado societies as primarily mobile foragers, and demonstrate the existence of sophisticated land use practices centered on maize-based polyculture.

**Fig. 8.**
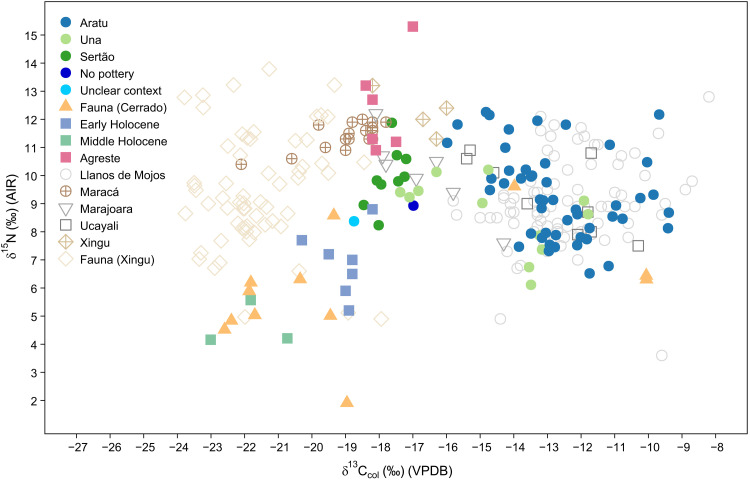
Human and fauna bone collagen δ^13^C and δ^15^N values from this study and previously published datasets (data S2 and S3). Individuals from the Aratu and Una pottery traditions, sertão pottery, rock shelters without pottery, rock shelters with unclear archaeological contexts, and faunal remains from the Cerrado biome (this work); early Holocene and middle Holocene humans from the Cerrado biome (*45*, *38*); humans from Agreste region (*39*); fauna (Xingu) and humans from Xingu region (*60*); Maracá humans (*61*); Marajoara humans (*62*); humans from Ucayali (*46*); and humans from Llanos de Mojos (*47*) (data S2 and S3).

When compared with previously published stable isotope data from humans in the Cerrado dated to the early and middle Holocene (*38*, *45*), our results indicate a dietary shift toward an increasing contribution of C_4_ foods, particularly maize, alongside greater meat intake in the late Holocene (Fig. 8). This increase in C_4_ resource consumption is evident in the δ^13^C_col_ analysis of individuals from the rock shelters, culminating in a diet based on maize among individuals from the Aratu open-air villages. These Aratu δ^13^C_col_ values are comparable to those from Ucayali (*46*) and Llanos de Mojos (*47*), urban-scale societies in Amazonian ecotones, where maize has been argued to have been cultivated under intensive agriculture with irrigation systems and earth works (Fig. 8) (*5*, *48*). However, the degree of similarity between these regions should not be overstated, as the Cerrado lacks the extensive landscape engineering and hydraulic infrastructure seen in regions like Llanos de Mojos (Casarabe culture). This comparison is better framed in terms of parallel developments in tropical savanna societies rather than implying equivalent socioecological systems. Furthermore, recent work from the southwestern Amazon emphasizes that even in regions with extensive agricultural infrastructure, food production systems remained highly diversified and included a wide range of cultivated and wild resources (*49*). Engaging with this perspective helps frame maize not as the sole organizing crop but as one key component within broader systems of landscape management.

In contrast to proposed (and debated) systems of intensive maize “monoculture” (*5*), the Cerrado data indicate a long-term reliance on diversified maize-based polyculture systems embedded within broader food production strategies. The multiproxy evidence from the Casarabe culture (*5*, *47*), as well as from other cultural regions of the Llanos de Moxos, including the north-central Exaltación Lakes region (*49*), likewise indicates diversified agricultural systems in which maize constituted one important component among a broad spectrum of cultivated and wild resources. However, it is unclear whether these different societies developed maize intensification independently or whether it spread through diffusion or migration. Maize reached the Cerrado 3700 cal. B.P., more than 2000 years before the emergence of the Aratu villages (*18*). Comparisons between archaeological and modern maize genomes across South America demonstrate that the archaeological samples analyzed are most closely related (*6*). At the same time, modern maize in the southwestern Amazon shows genetic affinities with modern Andean maize and with archaeological maize from the Cerrado dated to c. 650 cal. B.P. (*6*). With no evidence of Andean-Pacific maize lineages in the Cerrado before 650 cal. B.P., the emergence of the Aratu villages cannot be linked to an expansion of maize from the Andes. Instead, maize macroremains dated to 1100 to 500 cal. B.P. from Cerrado sites indicate that local cultural practices sustained crop diversity and the persistence of morphological traits of domesticated and primitive plants well after domestication (*50*). Together with our data, this evidence supports the hypothesis that the emergence of the Aratu tradition may reflect a local development (*14*), involving a deep knowledge of the local ecosystems and land use practices that maintained biodiversity for centuries.

Our stable isotope results support evidence of cultural and economic variability in central-eastern South America during the late Holocene. As in the Amazon, Cerrado societies developed diversified food production systems based on both domesticated and wild plants. Stable isotope analysis shows that individuals from Cerrado and Caatinga rock shelters had varied diets based on diverse plant resources, whereas individuals from Aratu tradition villages relied heavily on maize. Only a few individuals across contexts exhibited similar diets, highlighting the need for future research, where analyses of sexual dimorphism and ancient DNA could help clarify whether these similarities reflect exchange, intermarriage, or cultural practices. Our dataset does not yet clarify whether maize consumption followed a progressive increase, since contemporary individuals showed variable diets. Instead, the evidence suggests that the transition to maize as a staple food likely occurred between 4000 and 1200 cal. B.P., a critical but poorly understood period, lacking directly dated human skeletal remains. This chronological gap is a critical interval for understanding the transition toward increased maize consumption, but it remains poorly documented in the current dataset and stands as a key priority for future research. In this context, the complex evolutionary legacy of maize in South America, as highlighted by Kistler and colleagues (*6*), warrants further integration with future archaeological datasets to fully capture the transitional phases of maize adoption and intensification. While maize was a principal dietary component in the Llanos de Mojos between 1250 and 550 cal. B.P. (*47*), palaeoecological data and genomic evidence from maize remain insufficient to clarify possible interactions between societies from the two savannahs, the Cerrado and the Beni Savanna. Circular villages in the Upper Xingu, which emerged around 1100 cal. B.P. may have been influenced by migration or diffusion in a west-east movement linked to the Llanos de Mojos and indirectly to Andean states (*30*), but such a process is harder to detect for the Aratu tradition, especially since some of its earliest sites occur in the Cerrado core and the Brazilian coast (fig. S35). Overall, the rise of the Aratu villages appears to reflect long-term interactions between humans and Cerrado environments (*14*), with these widely distributed societies developing local adaptive land use strategies. The findings highlight maize-based polycultural systems as a viable and enduring alternative to models emphasizing intensive monoculture, underscoring the diversity of food production pathways in precolonial South America. These insights not only refine the archaeological record but also highlight the deep history of human practices relevant to the preservation of the Cerrado today.

## MATERIALS AND METHODS

### Samples

We analyzed samples from individuals linked to the Aratu tradition (*n* = 69), Una tradition (*n* = 14), pottery makers from the sertão in the Caatinga biome (*n* = 14), an archaeological site with macrobotanical remains of domesticated and wild plants, but without pottery (*n* = 1), and individuals whose cultural context remains uncertain from rock shelter (*n* = 3) (text S2). The number of individuals per site is presented in table S17. The cultural affiliation of individuals associated with the Aratu and Una traditions was identified primarily through ceramic remains, with landscape context also considered in cases where pottery displayed variable characteristics and lacked the full set of attributes of these traditions stricto sensu (text S2). Of the 101 individuals analyzed, 39 had bone and teeth analyzed, 50 had only bones, and 12 had only teeth (data S2). Ribs were preferably used to extract bone collagen, as they represent a dietary signal for approximately the last 10 years of life (*51*). When ribs were not available, we used long bone or mandible fragments which represent a longer period of life. When analyzing tooth enamel, we sampled molars, preferably third or second molars, formed between 7 to 16 and 3 to 8 years of age, respectively (*52*), to avoid the nursing or weaning effect.

To better interpret the results of the human analyses, we established the isotopic baseline using archaeological faunal remains. The faunal collection for the region is quite limited due to taphonomic processes. Faunal remains were identified by E. Chim and E. Mayer using modern reference collections housed in the University of São Paulo. The faunal isotopic baselines include 12 bones and 17 teeth from medium herbivorous mammals (Cervidae); omnivorous mammals such as peccary (Tayassuidae), opossums (*Didelphis* sp.) and rodents; the insectivorous mammal giant anteater (*Myrmecophaga tridactyla*); the carnivorous felid (Felidae); and reptiles, such as lizards (*Tupinambis* sp. and *Ameiva* sp.) (data S2). The specimens were selected from the archaeological sites of Lapa do Boquete, Gruta dos Milagres, GO-RS-01, GO-JA-03, and Vau I.

### Radiocarbon dating

Bone and dentine samples from 17 individuals underwent physical and chemical pretreatment at the Max Planck Institute of Geoanthropology’s Radiocarbon Laboratory. Following an acid-base-acid protocol based on the Longin method (*53*), collagen was extracted, filtered, and freeze-dried. This collagen was then combusted and graphitized at the Max Planck Institute of Biogeochemistry’s ^14^C-Analysis Laboratory before being measured using a MICADAS (Mini Carbon Dating System) AMS (Accelerator Mass Spectrometry) system (*54*, *55*), with data analysis performed according to established methods (*56*). An additional eight bone collagen and dentine samples were prepared and dated at Beta Analytic. Calibrations of ^14^C ages using SHCal20 atmospheric calibration curve and Bayesian chronological modeling results for radiocarbon dates were performed using OxCal 4.4.

### Stable isotope analysis of collagen

Collagen pretreatment of bone samples for stable isotope analysis was conducted at the Max Planck Institute of Geoanthropology, in Jena, Germany, according to published laboratory protocols (*57*). Human and faunal bones were cleaned using air abrasion or scalpel and then were cut into small pieces (300 to 1200 mg). To avoid cross-contamination, the workspace and the tools were cleaned with ethanol before sampling each bone. The samples were placed in 12-ml glass test tubes and demineralized by immersion in 0.5 M HCl and placement in a fridge. The acid was changed every 48 hours for 2 to 14 days. Once demineralized, each sample was rinsed three times with ultrapure MilliQ water and immersed in pH3 solution and placed in a heating block at 70° for 48 hours. Immediately after gelatinization, the soluble collagen solution was filtered using EZEE filters to remove insoluble residues. Samples were lyophilized in a freeze dryer for 48 or 72 hours. After freeze drying, each collagen sample was weighed to calculate the percent collagen yield. If the collagen yield was superior to 1%, then two 0.3- to 0.6-mg aliquots of each sample of purified collagen were weighed into tin capsules to be analyzed for δ^13^C and δ^15^N in duplicate using the Thermo Fisher Scientific Delta V Advantage IRMS (isotope ratio mass spectrometer) connected to a Flash2000 Organic Elemental Analyzer at the Max Planck Institute of Geoanthropology. Isotopic values are reported as the ratio of the heavier isotope to the lighter isotope (^13^C/^12^C or ^15^N/^14^N) as δ values in parts per mill (‰) relative to international standards, Vienna Pee Dee Belemnite (VPDB) for δ^13^C and atmospheric N2 (AIR) for δ^15^N. Carbon and nitrogen isotopic values were calibrated against international standards registered by the International Atomic Energy Agency (IAEA) and the U.S. Geological Survey (USGS) [IAEA-CH-6 (δ^13^C = −10.49‰); IAEA-N-2 (δ^15^N = 20.3‰); USGS40 (δ^13^C = −26.39‰; δ^15^N = −4.52‰); and UREA (δ^13^C = −36.54‰; δ^15^N = −2.35‰)] and an in-house standard for precision checks [fish gelatin (δ^13^C = −15.7 ± 0.2‰; δ^15^N = 13.9 ± 0.1‰)].

### Stable isotope analysis of tooth enamel

Pretreatment of tooth enamel samples for stable isotope analysis was conducted at the Max Planck Institute of Geoanthropology, in Jena, Germany, according to published laboratory protocols (*58*). Human and fauna teeth were cleaned using air abrasion. To avoid cross-contamination, the workspace and the tools were cleaned with ethanol before sampling each tooth. Using a diamond-tipped drill, ~10 mg of tooth enamel powder was sampled onto a piece of tin foil and placed in microcentrifuge tubes of 1.5 ml. To obtain a bulk sample, enamel was collected from the entire occlusal side of the tooth. In samples from small animals, such as rocky cave, microrodents, opossums, lizards, and fish, the roots of the teeth (preferably molars) were removed, and each tooth was crushed using an agate mortar and pestle. For each set of 20 samples, a blank sample was added, whose tooth enamel isotopic values are known. The samples were pretreated by adding 1 ml of 0.1 M acetic acid to each one, shaken, and left to rest for 8 min, when they were centrifuged for 2 min at 0.9 rpm. After that, the acid was removed using a pipette, and the samples were rinsed with MilliQ water and centrifuged three times. Then, the samples were placed in the freezer for 24 hours and lyophilized in a freeze dryer for 4 to 8 hours. From each sample, ~3 mg of pretreated enamel powder was transferred to 12-ml borosilicate glass vials to be analyzed for δ^13^C and δ^18^O using the Thermo Fisher Scientific Delta V Advantage IRMS connected to Gas Bench II. Carbon and oxygen isotope values were compared against international standards registered by the IAEA and the USGS [IAEA-603 (δ^13^C = 2.46‰; δ^18^O = −2.37‰); IAEA-CO-8 (δ^13^C = −5.76‰; δ^18^O = −22.7‰); USGS44 (δ^13^C = −42.2‰; δ^18^O = −13.8‰); and NBS18 (δ^13^C = −5.04‰; δ^18^O = −23.2‰)], and an in-house standard for precision checks [horse (δ^13^C = −12.4 ± 0.2‰; δ^18^O = −7.6 ± 0.2‰)].

### Statistical analysis

Statistical analyses were conducted using RStudio (*59*). All δ^13^C, δ^15^N, and δ^18^O datasets were tested for normality using a Shapiro-Wilk test and for homogeneity of variance using a Levene’s test. The significance of δ^13^C, δ^15^N, and δ^18^O value variance was assessed between herbivorous, omnivorous, and carnivorous fauna, as well as among humans from different cultural affiliations and biomes. When data met the assumptions of normality and homogeneity of variances, ANOVA was performed followed by Tukey post hoc test. For normally distributed data with unequal variances, Welch’s ANOVA and Games-Howell post hoc tests were used. When data did not meet the assumption of normality, the nonparametric Kruskal-Wallis test was applied, followed by Wilcoxon pairwise comparisons as a post hoc procedure. The complete statistical results are presented in tables S1 to S16.

### Ethics and permits

Permissions for stable isotope analysis and radiocarbon dating abroad were obtained from de National Institute of Historic and Artistic Heritage in Brazil (process nos. 01450.005371/2023-47, 01450.003290/2023-11, 01516.000865/2018-66, 01516.000877/2018-91, and 01508.000110/2021-66). Sampling access to the archaeological material was granted by the local curators at the following institutions: Museu de Arqueologia e Etnologia da Universidade de São Paulo, Instituto de Biociências da Universidade de São Paulo, Museu Arqueológico do Carste do Alto São Francisco, Instituto Goiano de Pré-História e Antropologia da Pontifícia Universidade Católica de Goiás, Museu Antropológico da Universidade Federal de Goiás, Universidade Federal do Recôncavo da Bahia, Universidade Federal da Bahia, and Museu de História Natural e Jardim Botânico da Universidade Federal de Minas Gerais e Universidade Estadual da Paraíba.

## Supplementary Materials

### The PDF file includes:

Supplementary Text Figs. S1 to S38 Tables S1 to S17 Legends for data S1 to S4 References
