# Supplementary material for: Maize-based polyculture, not monoculture, sustained precolonial societies in the Brazilian Cerrado
Source: Sci Adv. 2026 Jul 15;12(29):eaef7066. doi: 10.1126/sciadv.aef7066 (PMC13371897; doi:10.1126/sciadv.aef7066)
Supplement: Supplementary file 1 — Supplementary Text Figs. S1 to S38 Tables S1 to S17 Legends for data S1 to S4 References [file sciadv.aef7066_sm.pdf]

Science Advances
AAAS

Supplementary Materials for

Maize-based polyculture, not monoculture, sustained precolonial societies in the Brazilian Cerrado

Eliane Chim *et al.*

Corresponding author: Eliane Chim, chim@usp.br; Patrick Roberts, roberts@gea.mpg.de; André Strauss, strauss@usp.br

Sci. Adv. **12**, eaef7066 (2026)
DOI: 10.1126/sciadv.aef7066

The PDF file includes:

Supplementary Text
Figs. S1 to S38
Tables S1 to S17
Legends for data S1 to S4
References

Other Supplementary Material for this manuscript includes the following:

Data S1 to S4

1. Additional environmental, archaeological and ethnolinguistic background

Environmental contexts

The Cerrado is the second largest biome of the South American continent, extending over an area of 2,045,000 km² at altitudes ranging between 300 and 900 metres. It receives an annual precipitation of between 750 and 2,000 mm and experiences a dry period lasting 5-7 months with frequent fires (*63*). This biome is home to the headwaters of three of the largest South American river basins (Amazon/Tocantins, São Francisco and Prata), and features a diverse landscape mosaic consisting of grassland, scrubby cerrado, gallery forests and dry forests (*63, 64*). More than 40% of the 12,000 plant species are endemic (*65*), despite increasing deforestation, which has resulted in a 46% loss of native vegetation (*66*). In the east, the Cerrado has transitional environments with the Caatinga to the north and the Atlantic Forest to the south. The Caatinga biome is a seasonally dry tropical forest in a semi-arid region, which extends over an area of 850,000 km². With a dry period that can last for more than 8 months (*63*), it receives annual rainfall of less than 1,000 mm, with some regions experiencing even drier conditions and showing significant trends towards increasingly arid conditions (*67*). In some areas, non-woody plants comprise over 60% of the species diversity in the biome, with pockets of plant biodiversity in crystalline peneplains, sedimentary environments, inselbergs, riverine forests, and ecotonal areas (*68*). In contrast to these biomes, the Atlantic Forest is a rainforest, which is closely related to the coast. The climate in these regions varied during the Holocene. Speleothem records indicate abrupt fluctuations in precipitation in the current Cerrado, with wetter events lasting approximately 300 years in the early and middle Holocene and a maximum of 100 years in the late Holocene (*69*). Conversely, the Caatinga region experienced variations in precipitation levels which lasted for millennia, transitioning between wetter and drier conditions (*70*). Around 4,200 cal. BP, the climate abruptly shifted to a very dry state, resembling the current conditions, leading to an increase in the dominance of dry-adapted flora species characteristic of the biome (*71, 72*).

Archaeological background

The oldest ceramics found outside the Amazon in eastern South America, dated between 3,000 and 500 cal. BP, are generally classified as belonging to the Una tradition (*25, 73*). These ceramics, found in Cerrado rock shelters, are characterized by small, rounded vessels with a capacity of less than one litre, without painting or plastic decoration. They rarely feature a red engobe and have thin walls (ca. 0.4 – 10 mm) tempered with sand, caraipé or charcoal (*74–76*). Ceramic fragments are usually sparse and appear in archaeological layers with macrobotanical remains of both domesticated and wild plants (*18, 43, 77*). The exceptional preservation of organic materials in many associated sites has enabled the recovery of artifacts made of bone, wood, and plant fibers (*18, 43, 44, 78–80*), as well as funerary contexts (*81, 82*). Due to their simple design and wide chronological and spatial distribution, it is challenging to affirm that Una tradition represents a cultural or population unit. However, due to the limited evidence available to assess cultural variability in this context, human occupations from Cerrado rock shelters containing ceramics are here broadly considered as part of the Una tradition. This simple pottery appears in rock shelters previously occupied by non-ceramic populations and, aside from the pottery itself, in many sites no other technological or cultural changes were detected in the archaeological record, supporting the hypothesis of cultural continuity (*16*). Another hypothesis suggests that late pottery from the Una tradition and the Aratu tradition may be culturally related,

representing different expressions of the same society, with varying ceramic styles found in settlements located in distinct landscapes (e.g. sheltered and open-air) (*83*).

The sudden appearance of large circular villages after 1,200 cal. BP was a widespread phenomenon, extending from southeastern Amazonia, in the Upper Xingu region, and across the Cerrado to the Atlantic coast. These villages are composed of dozens of residential units with circular, elliptical or semicircular shapes (*15, 27*). Research has suggested that the emergence of these villages could be related to cultural, demographic, or linguistic dispersals (*16, 17, 28*). It has also been suggested that this development may reflect the west-to-east diffusion of cultural practices, related to interactions between Andean and lowland groups from savannas in Bolivia and Central Brazil (*30*), or an expansion linked to the spread of Arawak languages and the second wave of maize traditions (*6*). In the Upper Xingu, the emergence of this settlement pattern is related to the migration of Arawak speakers (*30*). However, this connection is not demonstrated in the broader eastern region, where the Uru ceramic tradition is found in the western Cerrado and the Aratu tradition extends from the Cerrado to the Atlantic coast (*14, 74*). The emergence of both Uru and Aratu villages has been viewed more as the result of a long and continuous process of integration among culturally distinct groups, as well as a local response to demographic pressures and external interactions (*14*).

The morphological similarity between archaeological and ethnohistorical documented pottery, characterized by shallow, flat-based vessels and large griddles, sometimes with handles or appendages, has led to the interpretation that manioc was a staple food among societies of the Upper Xingu and the Uru ceramic tradition (*14, 30*). The different shapes of the vessels of the Aratu tradition, the ancient presence of maize in the Cerrado and the frequent placement of villages near gallery forests, where fertile and humid soils could have favored the cultivation of this crop, contributed to the hypothesis that maize was a staple food (*14*). The Aratu pottery tradition includes pear-shaped, conical, globular or twin vases, from one litre to up to 420 litres in volume (*35*), made with mineral tempers of crushed sherd or sand, with smoothed surfaces, graphite or red engobe and almost no plastic or painted decoration (*35, 84*). This pottery, distributed over a large territory, is not homogeneous and has been classified into 'phases' of the same tradition according to the shape of the vessels (*15*). However, the absolute dates and the diversity of shapes represent regional variability more than diachronic phases (*16*). A feature shared across the Aratu dispersal area is the use of large pear-shaped pots as funerary urns, which have been found isolated or together, in the area outside the village or in archaeological sites known as 'urn fields', which may have more than a hundred human burials (*84*). The presence of human burials allows direct research into subsistence practices.

Similarities between archaeological context and present-day ethnolinguistic groups

Although it is not always possible to establish reliable links between archaeology, linguistics and ethnohistory, especially in the context of Indigenous societies before and after the European invasion, some similarities between Aratu and Una archaeological traditions to societies speaking Macro-Jê languages have been identified (*15, 17, 85*). Macro-Jê is a large language stock comprising ten language families, spoken across the lowlands of South America in the area south of the Amazon River (*86*). Among these, the Jê linguistic family is the one with the greatest diversity in number of languages, divided into two branches, *Cerratense* (dispersed in the Cerrado region) and *Meridional* (dispersed in the Southern Brazil) (*86*). There is a clear

overlap between the ethnohistorical places of Jê *Cerratense* branch and the area of dispersion of Una and Aratu archaeological sites (*15, 17, 87*). In addition, circular or semicircular villages were documented in societies of the Jê linguistic family, which occupied and still occupies central-eastern Brazil (*14, 85*). Just as archaeological evidence indicates that the origin of Una and Aratu traditions is the Cerrado, genetic evidence also suggests the origin of the Macro-Jê group in this biome, with an intricate and nonlinear mode of dispersion (*88*). Furthermore, genomic data from a skeleton coming from the Vau I site, an open-air village also studied in this research, showed strong similarities with a Jê-speaking group from Central Brazil (*89*). This does not mean that a direct connection between these archaeological traditions and the Macro-Jê languages is accepted, but there may be a relationship between the archaeological cultures and the ancestors of the Macro-Jê stock. It has been suggested that the word maize is a loanword in Macro-Jê stock languages, possibly of Andean origin and transmitted through Arawak languages (*6, 90*). However, this claim currently lacks linguistic support, as the Purí, Karirí and Yaathê languages families, in which this loanword occurs, are not part of the Macro-Jê stock (*86*), and the origin of the word for maize in the Karajá language family has not yet been accurately traced (*86*).

2. Archaeological sites

Abrigo do Ângelo

Abrigo do Ângelo (20°17'28"S 45°47'30"W, state of Minas Gerais, municipality of Doresópolis) is a rock shelter site (Fig. S1) located in the Cerrado biome, measuring approximately 66 m^2 with a height of 2 meters. The site features an open-air area in front of the sheltered zone, in which a sinkhole is causing erosion of the sedimentary package. The site contains chipped lithics, ceramics not assigned to any archaeological tradition, faunal remains, and three human burials (Fig. S2) (*91*). The three human burials were excavated in 2010 by Gilmar Henriques Jr. as part of a project conducted by the Museu Arqueológico do Carste do Alto São Francisco (MAC). Two of the burials appear to be secondary (Burials 01 and 03), containing bones from more than one individual (Fig. S3) (*91*). Here, we analyzed a deciduous molar tooth and a long bone fragment from one individual of a secondary burial (Burial 03, Individual 1) and dated the collagen from the tooth root (626-526 cal. BP, Data S1). This individual is associated with a ceramic occupation without cultural classification. The material is part of the collection of the MAC (Pains, MG).

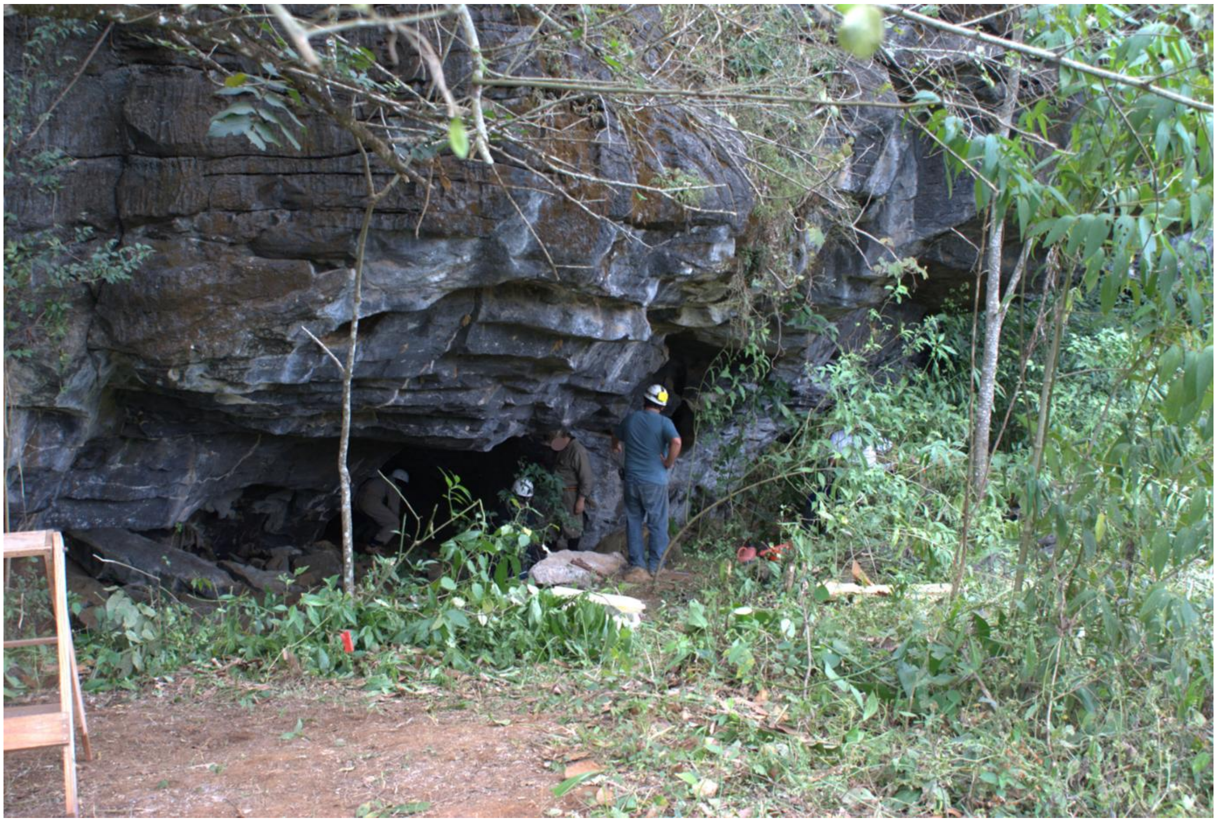

Fig. S1. Abrigo do Ângelo
External view of the rock shelter. Photo Credit: Gilmar Henriques.

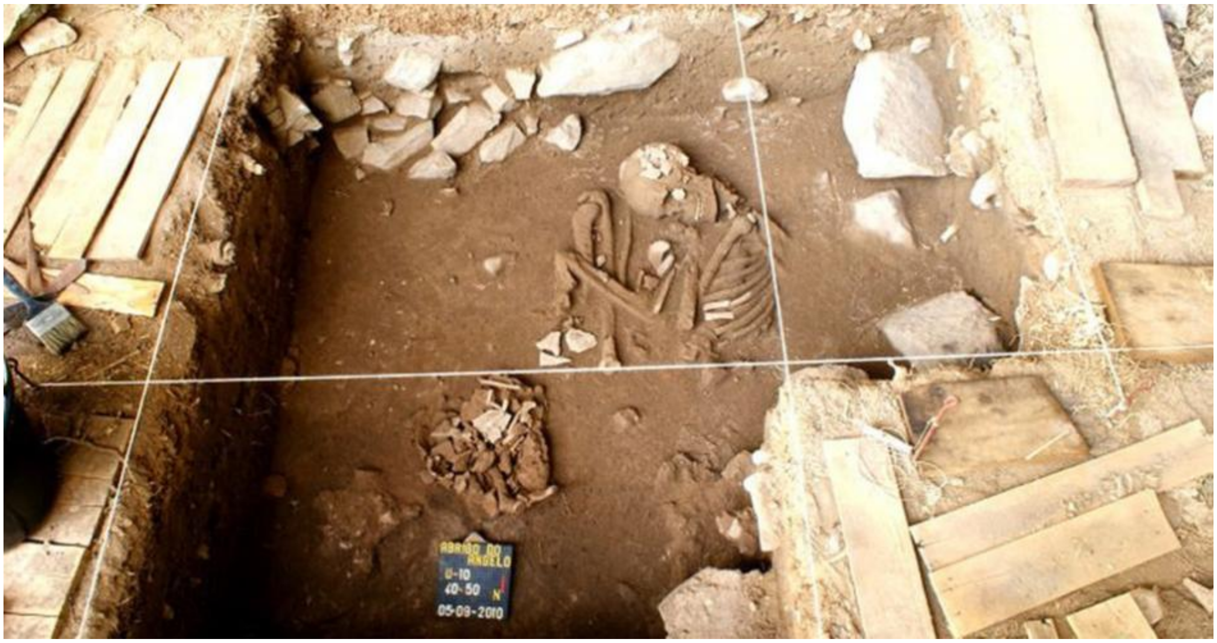

Fig. S2. Abrigo do Ângelo
Excavation with exposure of three human burials. Photo Credit: Gilmar Henriques.

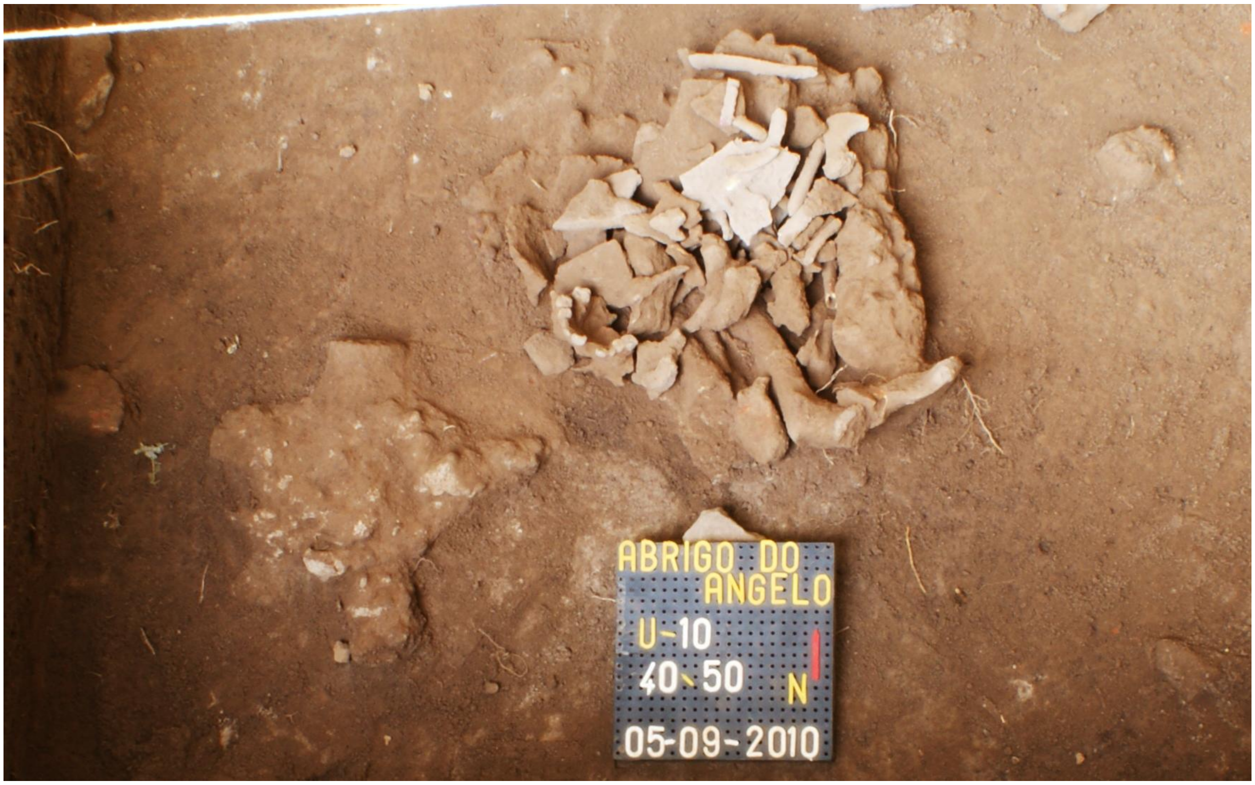

Fig. S3. Abrigo do Ângelo
Excavation with exposure of Burials 1 (left) and 3 (center). Photo Credit: Gilmar Henriques.

Abrigo Tuvira (GO-Ni-217)
The Tuvira rock shelter (14°38'34.29"S 49° 1'29.11"W, state of Goiás, municipality of Barro Alto) is an archaeological site located in a sandstone escarpment on the right bank of the Almas River, within the Araguaia-Tocantins River basin and the Cerrado biome. Spanning 25 m², the site was rescued in 1998, in a project conducted by the Museu Antropológico of the Universidade Federal de Goiás, before being submerged by the Serra da Mesa hydropower plants (*92, 93*). During excavations, three main layers of human occupations were identified. The oldest was dated between 8,400 and 7,900 cal. BP, the intermediate around 1,740 cal. BP and the most recent between approximately 670 and 440 cal. BP (*94*). A human burial was discovered associated with the most recent layer, consisting of a fully articulated skeleton of a single individual, with a pottery vessel from the Una tradition placed near the feet (*92*). In this study, we analyzed a femur fragment from this individual. The material is part of the collection of the Museu Antropológico of the Universidade Federal de Goiás (Goiânia, GO).

Água Limpa
Água Limpa (21° 0'30"S 48°29'0"W, state of São Paulo, municipality of Monte Alto) is a multi-period open-air site. It lies in the southern Cerrado, within the ecotone with the Atlantic Forest, in the Rio Grande basin, a tributary of the Paraná River (Prata River Basin). Archaeological research at the site was conducted under the coordination of Dr. Marcia Angelina Alves, as part of the TURVO project in 1993 and 1994 (*95*). The site covers approximately 7.000 m² and

comprises two main areas with archaeological remains (*95*). One area represents a village with nine housing structures, identified by dark, circular-shaped sediment patches (*95*). The other area contained combustion structures, smoothed undecorated pottery, painted pottery, chipped and polished stone tools, zooarchaeological remains and 11 human burials, of which nine were buried directly in the soil and two in undecorated funerary urns (*95*). Although not considered Aratu tradition *stricto sensu*, the human occupation associated with undecorated pottery, fragments of double vessels (also known as twinned vases), and human burials was linked to this tradition, as it displayed its characteristic elements. Here we analyzed long bone fragments from burials 01, 03, 04, 05, 06, 07, 08 and 09 and a rib from burial 02. The samples analyzed come from a period from 725 to 544 cal. BP (Data S1). The material is part of the collection of the Museu de Arqueologia e Etnologia of the Universidade de São Paulo (São Paulo, SP).

Água Vermelha

Água Vermelha (15°8'38"S 39°43'29"W, state of Bahia, municipality of Itaju do Colônia) is an open-air site located in the northeast part of the Atlantic Forest. A semicircular village with six elliptical-shaped patches of dark sediment, measuring between 10 and 25 meters in diameter, were identified in 2011 during research conducted by Dr. Carlos Etchevarne and Dr. Luydy Fernandes (*96*). Pottery fragments and chipped and polished lithic artifacts spread around the patches, over an area of 1,000m², were collected (*96*). Within this area, three funerary urns were excavated by them, all Aratu-shaped, with the Urn 1 featuring corrugated decoration (Fig. S4). Based on tooth eruption, Urn 1 contained an adult individual, Urn 2 held a subadult individual (~15 years old), and Urn 3 an infant (<1-year-old) (*97*). Here, we analyze a third molar from Urn 1 and a second molar from Urn 2, as well as bone samples from both individuals. The individual from Urn 1 is dated from 654 to 550 cal. BP (*96*) (Data S1). The material is part of the collection of the Laboratório de Arqueologia of the Faculdade de Filosofia e Ciências Humanas of the Universidade Federal da Bahia (Salvador, BA).

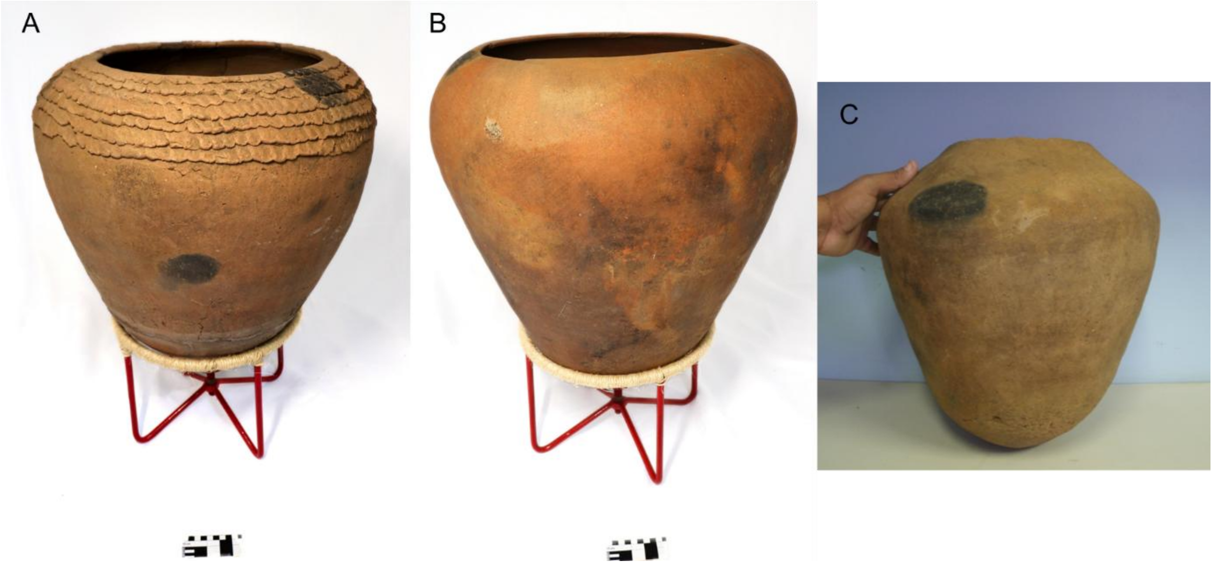

Fig. S4. Água Vermelha

Urn 1 (A), Urn 2 (B) and Urn 3 (C). Photo Credit: Mario Spock Fernandez (A and B) and Luydy Fernandes (C).

Alagamar

Located in the Caatinga biome, the Alagamar archaeological site (6°21'6.62"S 37°40'1.18"W, state of Paraíba, municipality of São João do Cariri) was excavated by Dr. Juvandi Santos in the 2000s. During this research, a funerary urn with typical features of pottery from the Sertão region was excavated. In this study, we analyzed a clavicle fragment from one individual buried in this urn. No additional information is available regarding the chronology of the site, however, by combining the archaeological context with ceramic dating from the region, it is estimated that the burial is less than 2000 years old. The material is part of the collection of the Museu de História Natural of the Universidade Estadual da Paraíba (Campina Grande, PB).

Antônio Beiraldo

A funerary urn from the Aratu tradition was discovered on the Antônio Beiraldo farm (20°20'49.25"S 45°51'30.61"O, state of Minas Gerais, municipality of Piumhí), located in the Cerrado biome within the upper São Francisco River basin. The open-air site lies near sinkholes in the limestone karstic region of Córrego do Cavalo and is crossed by a vicinal road (Fig. S5). Vehicle traffic along this road exposed pottery sherds scattered over an area of approximately 2,000 m², as well as the funerary urn. The urn was subsequently excavated in an emergency rescue operation conducted by Dr. Gilmar Henriques and Dr. Edward Koole in 2003 (Fig. S5), during the project "Arqueologia do Carste do Alto São Francisco". In this study, we analyze bone collagen from a long bone and dental enamel from a third molar belonging to Burial 1. The material is part of the collection of the Museu de Arqueologia e Etnologia of the Universidade de São Paulo (São Paulo, SP).

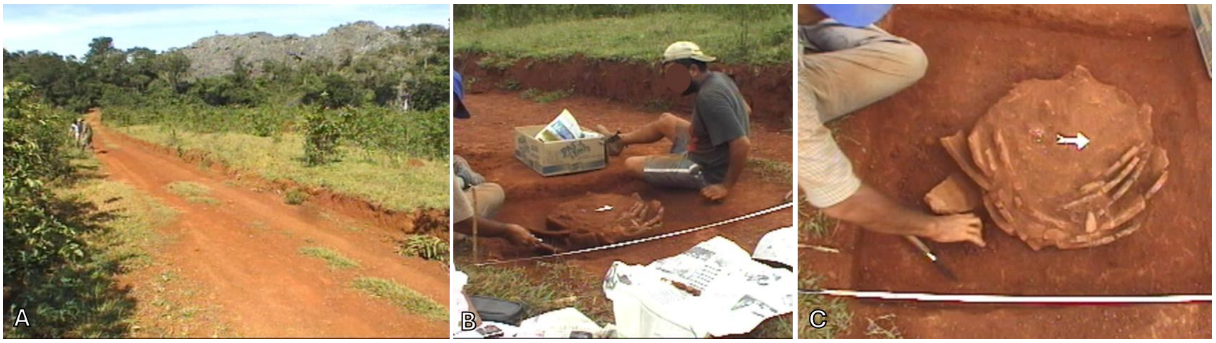

Fig. S5. Antônio Beiraldo

A) Road that crosses the archaeological site; B) excavation of the funerary urn; C) funerary urn. Photo Credit: Gilmar Henriques.

Bento

The Bento archaeological site (12°10'0.30"S 43°55'51.30"W, state of Bahia, municipality of Wanderley), situated in the Cerrado biome, was uncovered due to rapid rainfall-induced erosion that took place in 2021, which exposed at least nine human burials in funerary urns (Fig. S6) and hundreds of chipped and polished axe blades (Fig. S7). The pottery has been linked to the Aratu ceramic tradition. During the rescue of this previously unpublished site, in 2022, Eliane Chim, Henry Luydy Abraham Fernandes, Haruan Straioto, and Maria Ana Correia recovered pottery fragments, stone tools, and human skeletal remains. Here we analyzed a molar tooth and a long

bone fragment from Burial 8, a rib fragment from Burial 4 and a femur fragment from Burial 1. We dated Burial 4 (795-723 cal. BP) (Data S1). The site is located approximately 12 kilometers from the Piragiba site, which is also analyzed in this study. Radiocarbon dating indicates that human occupations in both Bento and Piragiba are contemporary (Data S1), although Piragiba has more datings, which extends its occupation chronology to 1043-664 cal. BP. The material is part of the collection of the Universidade Federal do Recôncavo da Bahia (Cachoeira, BA).

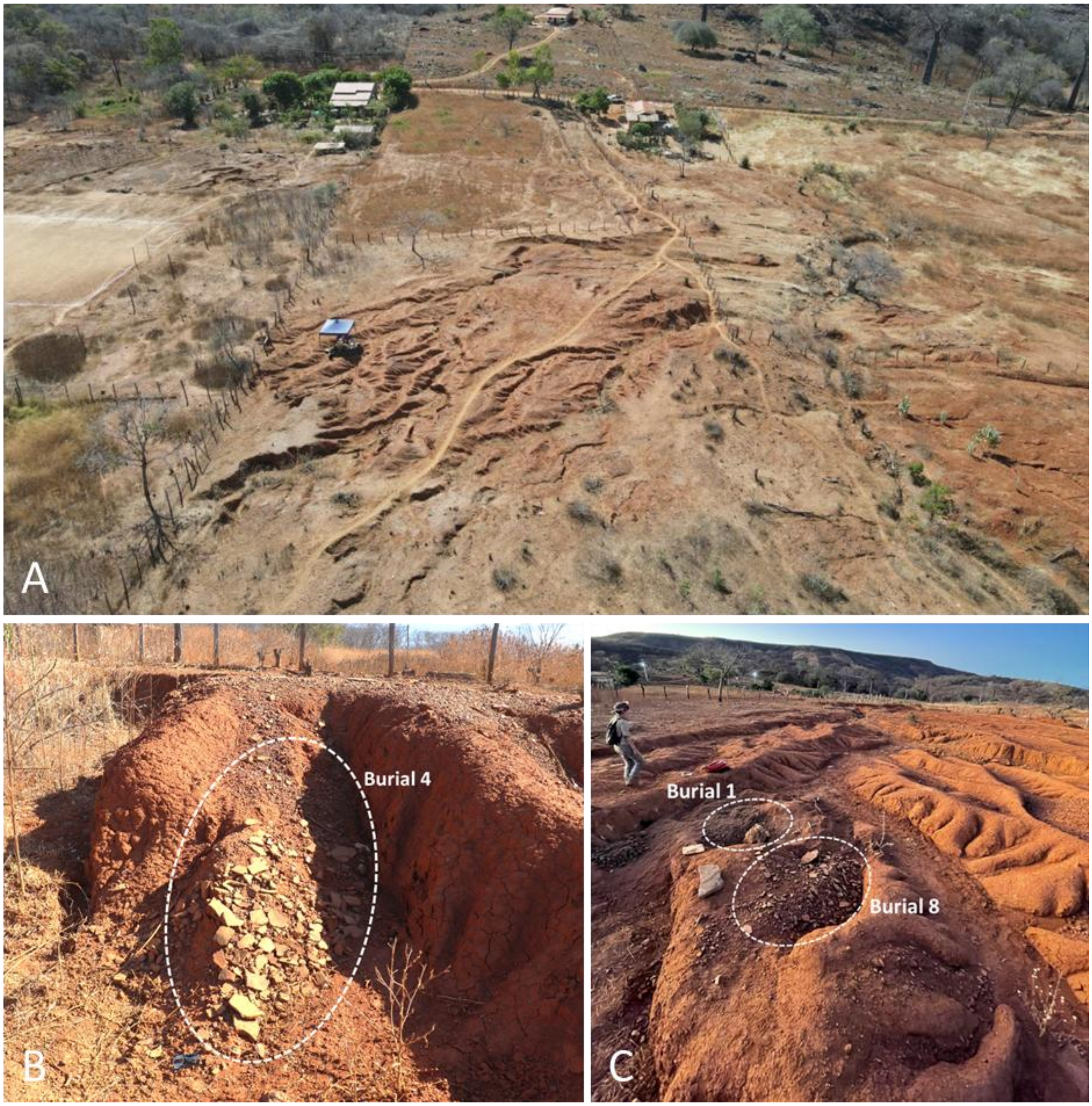

Fig. S6. Bento

A) Aerial view of Bento site, note the gully that is exposing the archaeological material; B) Pottery fragments of the Urn 4, where bone fragments were collected; C) area of dispersion of pottery fragments, bones and teeth from Urns 1 and 8. Photo Credit: Haruan Straioto (A); Eliane Chim (B, C).

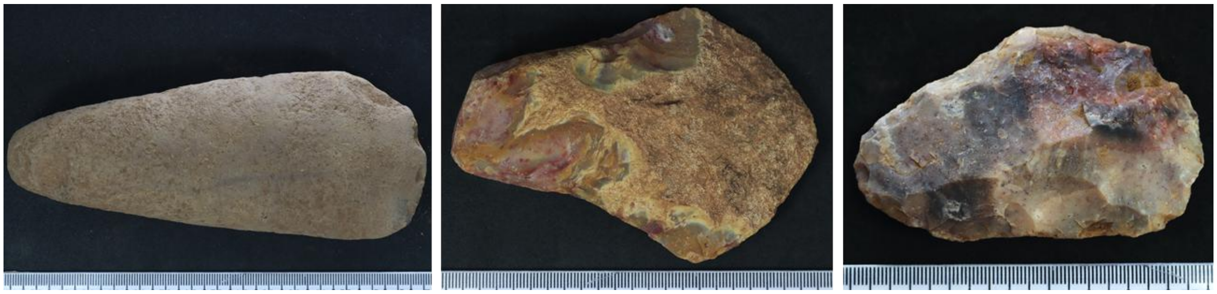

Fig. S7. Bento, lithic tools
Polished and chipped axe blades. Photo Credit: Gabo Garzon.

Bonsucesso (Go-RV.2)
Bonsucesso (16°51'1.44"S 50°2'55.68"W, state of Goiás, municipality of Palmeiras de Goiás), located in the Cerrado biome, is an open-air Aratu village consisting of 48 houses arranged in an oval pattern across an area of 370 by 380 meters, situated at the confluence of two streams within the Prata River Basin. The houses are identifiable by circular patches of black sediment, measuring between 7 and 17 meters in diameter (*98, 99*). Excavations conducted by Dra. Margarida Andreatta, between 1975 and 1977, uncovered chipped and polished stone tools, faunal remains, bones and horn artifacts, and pottery from the Aratu ceramic tradition (*99*). The pottery featured smoothed walls with minimal plastic decoration, spindle whorls, and double vases (also known as twinned vases) with no painting or other decoration (*99*). Located near the house "M12", a single funerary urn containing one individual was also excavated, and a sample of femur from this individual was analyzed in this study. Although charcoal dates from hearths of three houses indicated that the village was inhabited between ca. 1,200 and 750 cal. BP (*99*), bone collagen from Burial 1 was dated in the present study to 466-357 cal. BP (Data S1), suggesting a long term Aratu settlement or more than one occupation. The material is part of the collection of the Museu Antropológico of the Universidade Federal de Goiás (Goiânia, GO).

Buriti I (GO-JU-54)
Buriti I (16°11'43.10"S 50°18'56.70"W, state of Goiás, municipality of Sanclerlândia) is an open-air archaeological site with a diameter of 300 meters, located in the Cerrado biome. A funerary urn from the Aratu ceramic tradition (Fig. S8), standing 90 cm high and with a capacity of ca. 200 liters was excavated. The burial was accompanied by a small pottery vessel placed above the skull and a bowl also made of pottery, known in Brazil as 'opérculo,' serving as a lid for the urn. Additionally, small trapezoidal pendants made from bivalve shells, with two holes in the upper part, were found within the urn (Fig. S9). In this study, we analyzed a molar tooth from this individual. Another distinct archaeological occupation was identified at the same site, characterized by fragments of ceramics from the Tupiguarani tradition, a polished axe blade, and two small T-shaped tembetá made of semi-polished milky quartz (Fig. S9) (100). The material was excavated by Dr. Irmhild Wüst in 1992 and is part of the collection of the Museu Antropológico of the Universidade Federal de Goiás (Goiânia, GO).

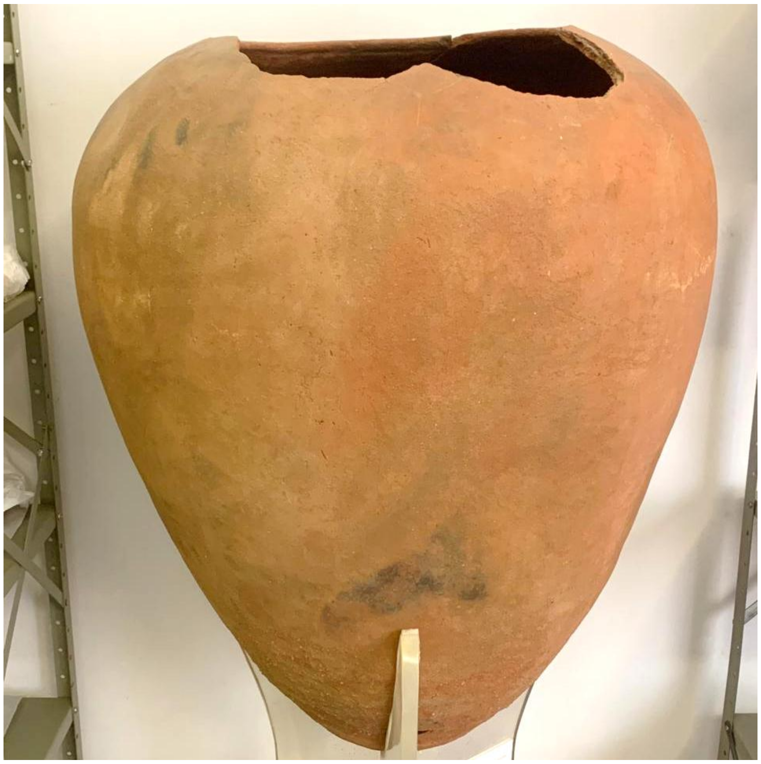

Fig. S8. Buriti I, funerary urn

Funerary Urn 1 standing 90 cm high and with a capacity of ca. 200 liters. Photo Credit: Rafael Lemos de Souza.

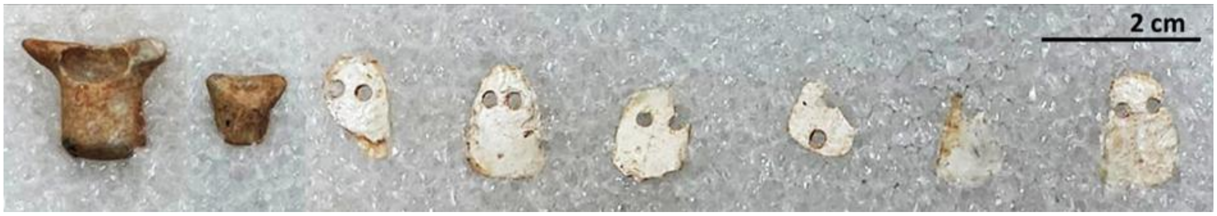

Fig. S9. Buriti I

T-shaped *tembetás* made of semi-polished milky quartz; small trapezoidal pendants made from bivalve shells, with two holes in the upper part, founded inside the Urn 1. Photo Credit: Diego Mendes.

<u>Camp – 01</u>

In the municipality of Camalaú (7°53'17.61"S 36°49'27.09"W, state of Paraíba), within the Caatinga biome, Dr. Juvandi Santos conducted archaeological research in 2007, collecting pottery, stone tools and human bones. Ceramic artifacts presented typical features of pottery from the Sertão region. Here, we analyzed a mandible fragment of one individual from this assemblage, which is part of the collection of the Museu de História Natural of the Universidade Estadual da Paraíba (Campina Grande, PB).

Embiara

In the Cerrado biome, Embiara (15° 4'18.47"S 49°58'2.85"W, state of Goiás, municipality Nova América) is an archaeological site discovered accidentally by local residents during maintenance of a rural road. In this site, a funerary urn from the Aratu tradition (Fig. S10) was excavated in an emergency rescue operation conducted by Dr. Diego Mendes and Dr. Rafael Lemos de Souza, in 2023. This was a grand burial of one individual. Alongside the human skeletal remains, two pendants (Fig. S11) and a circular object, probably a lip disc (*botoque*) or an ear plug (*alargador*), made of mineral material were found. In addition, the urn included a lid (*opérculo*) and was surrounded by three ceramic pots (Fig. S11). Here, we analyzed dental enamel and dentine collagen from a molar tooth from the individual of this urn. The material is part of the collection of the Museu Antropológico of the Universidade Federal de Goiás (Goiânia, GO).

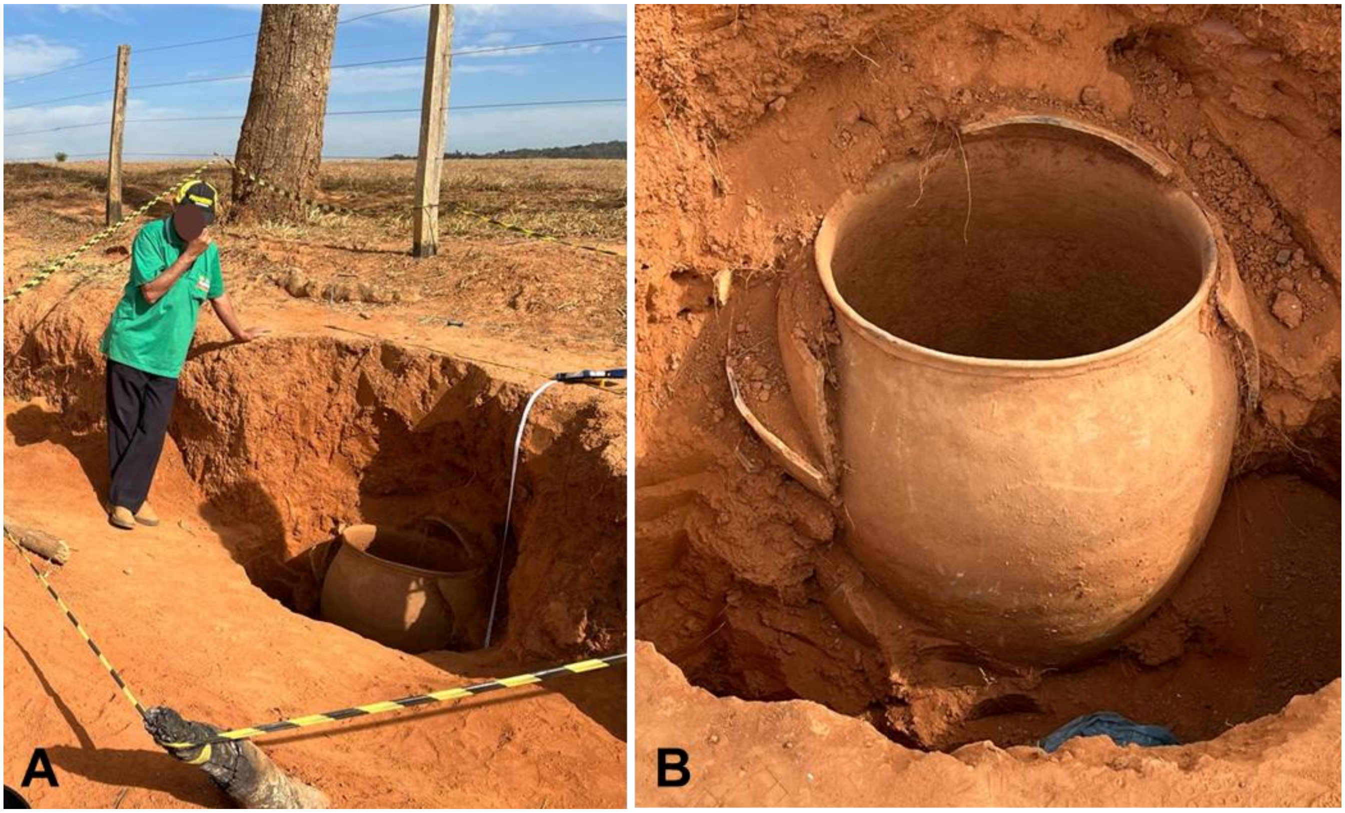

Fig. S10. Embiara

A and B) Excavation of the funerary urn. Photo Credit: Rafael Lemos de Souza.

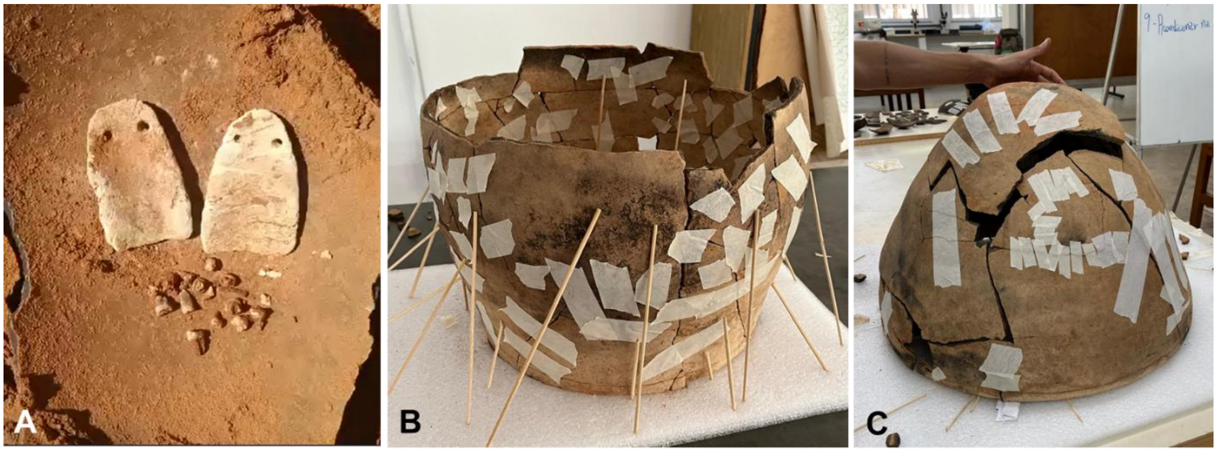

Fig. S11. Embiara

A) Two pendants and human teeth found inside the urn; B) Reconstruction of the ceramic vessel found close to the urn; C) reconstruction of the 'operculo' in the laboratory. Photo Credit: Rafael Lemos de Souza.

Furna dos Ossos

The Furna dos Ossos archaeological site (6°21'6.62"S 37°40'1.18"W, state of Paraíba, municipality of São João do Cariri) is located in a semi-arid region with a dry climate characteristic of the Caatinga biome in northeast Brazil (*101*). This small granite rock shelter measures 5 by 5 meters and features three openings, with many granite blocks scattered throughout (*101*) (Fig. S12). The site was discovered by the local population, who noticed human bones on the surface (*102*). Archaeological research led by Dr. Juvandi Santos, in 2008, involved collecting the bones, excavating the site, and uncovering a single rock painting (*101*). During this work, researchers also recovered faunal bones, pottery, and lithic materials. The ceramics, displaying regional characteristics typical of the Sertão, were dated using thermoluminescence (TL) to 890 ± 50 BP and 500 ± 30 BP (*103*) (Data S1). The human bones were found mixed together, making it impossible to identify individual burials. However, the minimum number of individuals was estimated to be 14 (*103*). In this study, we analyzed a phalanx fragment of one individual. The material is part of the collection of the Museu de História Natural of the Universidade Estadual da Paraíba (Campina Grande, PB).

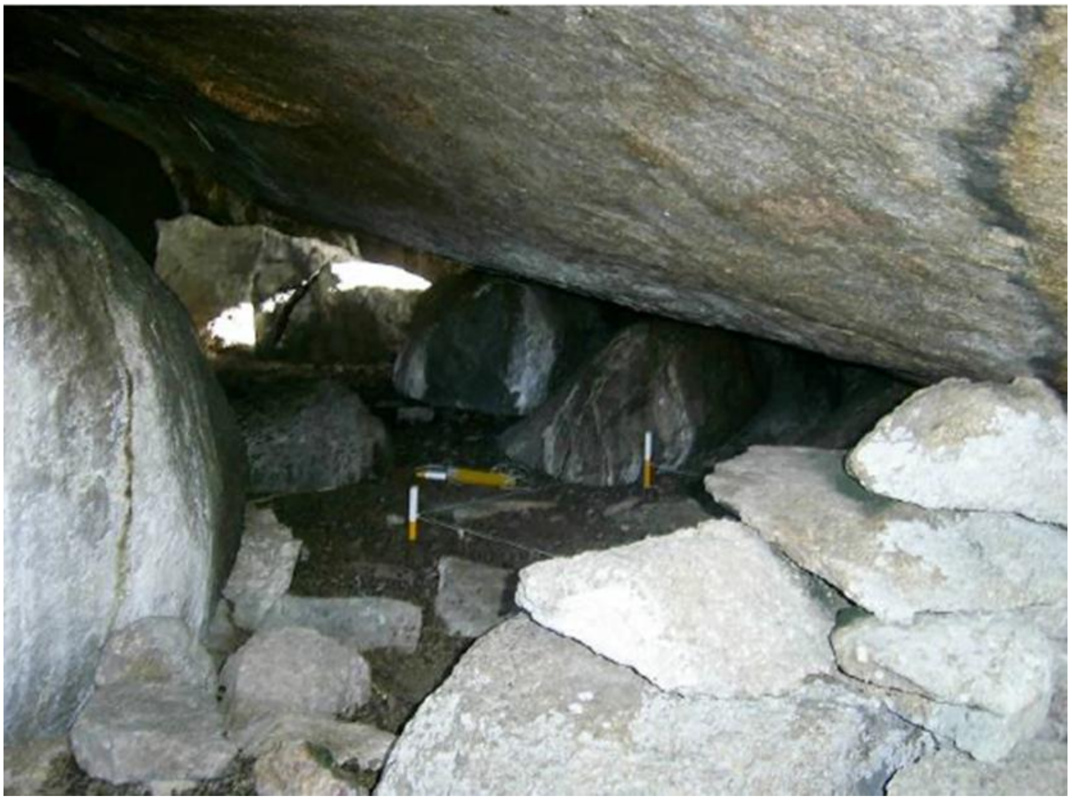

Fig. S12. Furna dos Ossos

Internal view of the Furna dos Ossos during archaeological excavation. Photo Credit: Juvandi de Sousa Santos.

GO-CP-14 (Abraão Dias)

In the Cerrado biome, the GO-CP-04 site (16°57'8.27"S 51°48'51.45"W, state of Goiás, municipality of Caiapônia) is a sandstone rock shelter with granite blocks at its base, measuring approximately 40 by 5 meters (Fig. S13). The sheltered area covered 160 m², though it is interrupted by a slope that has undergone erosive processes (*104*). Of this area, 105 m² were excavated between 1979 and 1981 by a team lead by Dr. Pedro Ignacio Schmitz (*29*). Rock paintings and engravings were identified at the site (Fig. S13). The excavation revealed a 30 cm sedimentary package containing pottery and lithic materials in all layers, as well as five hearths. The hearths, with diameters ranging from 50 to 80 cm and thicknesses from 5 to 20 cm, contained fragments of charcoal, fruits, seeds, maize cobs, and gastropods (*29*) (Fig. S14). During the excavation, a burial was identified in a shallow grave located between collapsed blocks, at a depth of 5 to 15 cm (*29*). Although it was a primary and complete skeleton, the bones were poorly preserved, and only a long bone, fragments of mandible and two molar teeth were recovered (*29*). Pottery sherds and gastropod shells were collected below the grave. Recent research at other sites in the Caiapônia region indicates that pottery was already present in local rock shelters around 800 cal. BP (Beta-434621) (*105*). Since this archaeological context features characteristics similar with the occupations of the Una ceramic tradition, such as well-preserved macrobotanical remains of domesticated and wild plants, simple, small, and rounded pottery, and is located in a rock shelter within the Cerrado biome, it is analyzed here within the framework of this archaeological tradition. From this burial, we analyzed a second molar and a long bone

fragment. The material is part of the collection of the Pontifícia Universidade Católica de Goiás (Goiânia, GO).

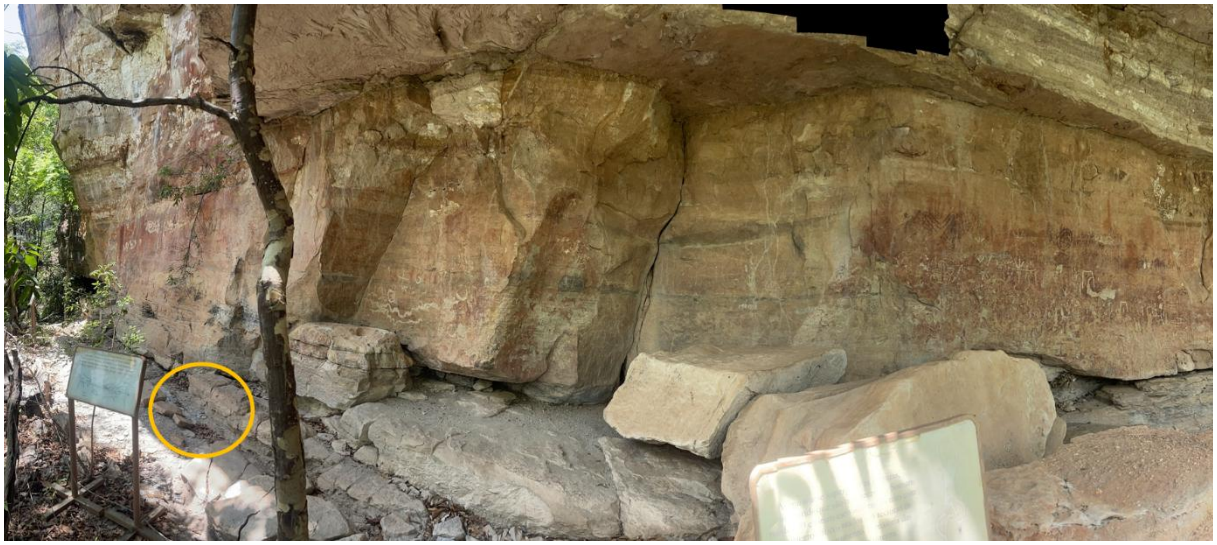

Fig. S13. GO-CP-04
Sheltered area of the GO-CP-04 site with rock art on the walls. Yellow circle indicates the area where Burial 01 was excavated. Photo credit: Sibeli A. Viana.

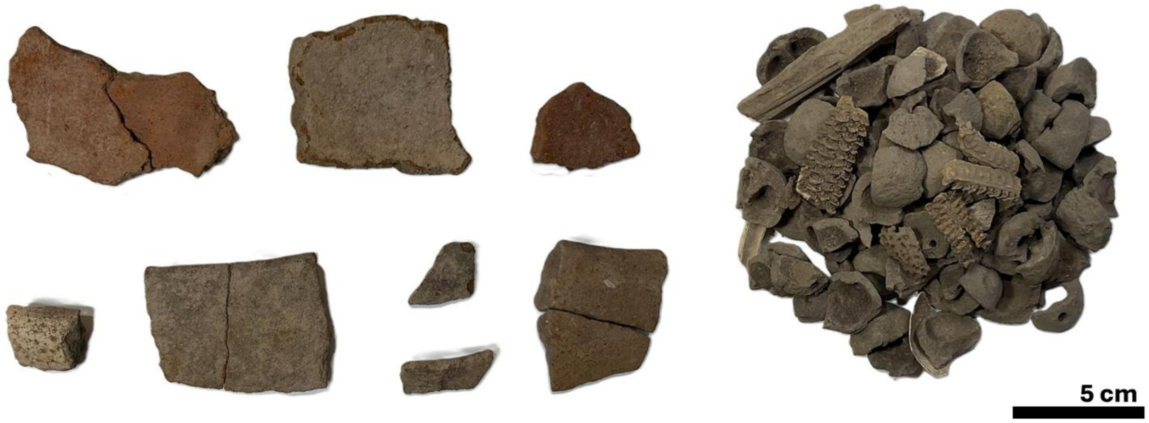

Fig. S14. GO-CP-04, pottery and macro-remains of plants
On the left, pottery fragments from the Una ceramic tradition; On the right, maize cobs, charred seed, wood fragments and gastropods shells. Photo Credit: Erica Rocha.

<u>GO-JA-01</u> <u>(Diogo</u> <u>Lemes)</u>
In the Cerrado biome, GO-JA-01 (18°19'12.04"S 51°48'0.02"W, state of Goiás, municipality of Serranópolis) is an archaeological site with evidence of human occupations throughout the Holocene. Most of the radiocarbon dates range from 12,700 to 7,300 cal. BP, reflecting a focus on pre-ceramic occupations during the initial research (*76*) (Data S1). However, there are also

radiocarbon dates from the late Holocene, from ca. 920 to 680 cal. BP, corresponding to occupations from the Una tradition (*76*) (Data S1). The site is a rock shelter that opens to the north-northeast, measuring 64 x 27 meters with a height of 34 meters at the drip line (Fig. S15). In this large chamber, which has 1,300 m², 52 m² were excavated, revealing a stratigraphic deposit between 150 and 200 cm thick (*76*). At the site, the collapsed blocks from the walls of the shelter served as an immediate source of raw material for lithic tools (*106*). In the earliest occupation periods of the shelter, limaces artifacts with unifacial shape predominate (Itaparica tradition), while in the more recent layers, bifacially chipped axes are present (Fig. S16). The site also contain rock engravings and paintings and faunal remains (*107*). Botanical materials were found throughout the stratigraphy, with greater abundance in the more recent layers.

The middle occupation (ca. 10,200 cal. BP to 7,300 cal. BP), called Serranópolis phase, contained macroremains of plants, such as cf. *Arachis hypogaea* (peanut), as well as wild plants (*108*). In the late Holocene occupation (ca. 920 to ca. 680 cal. BP), identified by the presence of the Una ceramic tradition, a greater variety of domesticated and managed plants was found, including *Gossypium* sp. (cotton), *Zea mays* (maize), *Anacardium* sp. (cashew), *Annona* sp. (araticum), *Hymenaea courbaril* (jatobá), and various palm species (*108*). The excavations were conducted by a team led by Dr. Pedro Ignacio Schmitz, between 1975 and 1982. According to the field report, six human burials were identified. Three burials were completely excavated and, of one, only the feet were excavated, as the body was at the limit of the excavation unit, in the stratigraphic profile (*76*). However, in the museum collection, at least four individuals are identified as "Burial 1," suggesting the possibility that the materials currently labeled as "Burial 1" may correspond to four distinct burials. This conclusion is supported by the presence of cranial bones from two adults and teeth from two subadults. Among the subadult individuals, one was determined to be approximately 8-9 years old, based on the presence of the upper left first molar and an erupting second molar. The other subadult was younger, identified by the presence of deciduous molars. Here we analyze a molar tooth and a rib from an adult individual, and the 2nd left upper molar from the individual of 8-9 years old. Radiocarbon dating from bone collagen confirms that 'Burial 1' is chronologically associated with the Una tradition (922-800 cal. BP) (Data S1). The material is part of the collection of the Pontifícia Universidade Católica de Goiás (Goiânia, GO).

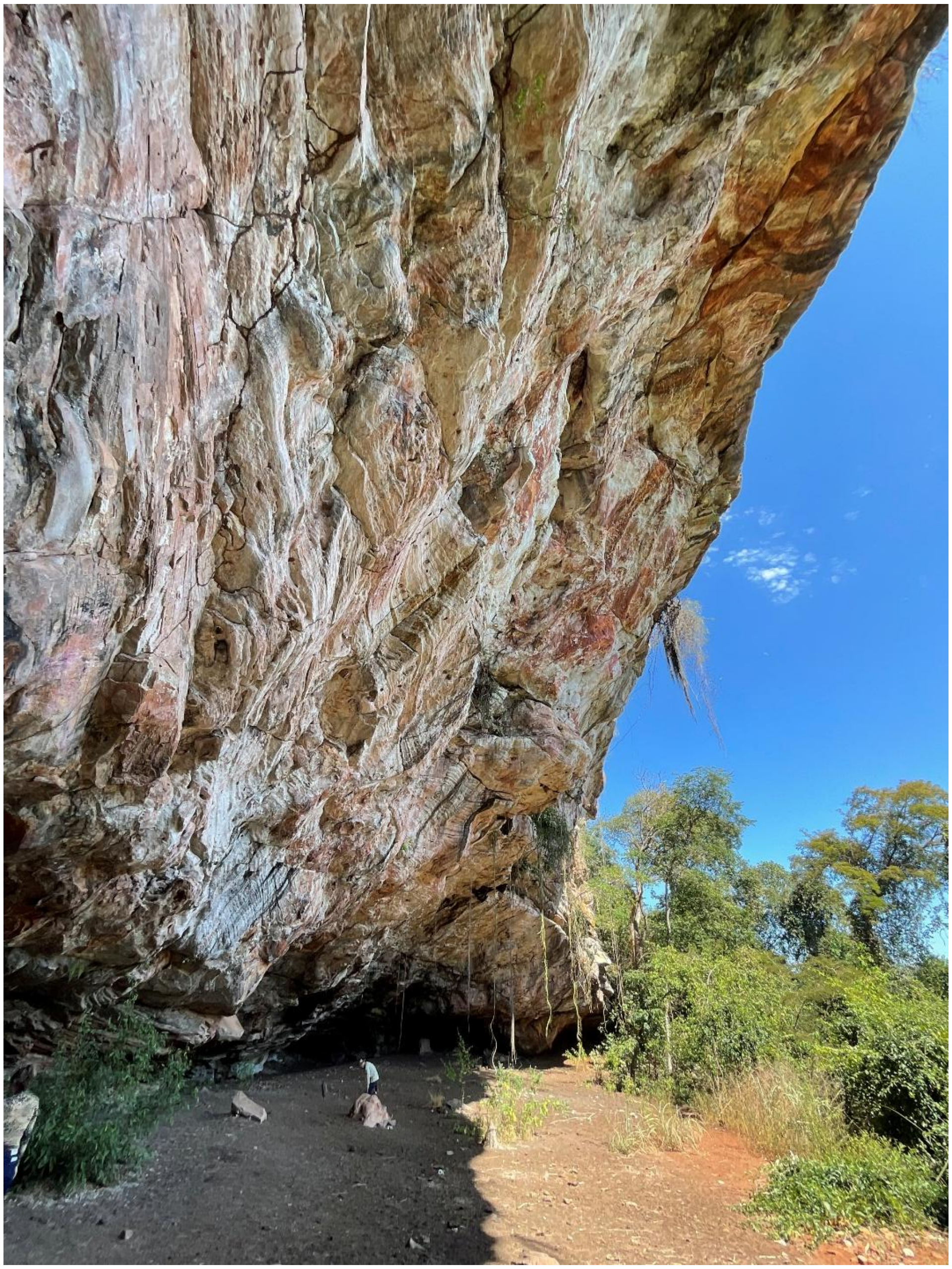

Fig. S15. GO-JA-01
Photo credit: Sibeli A. Viana.

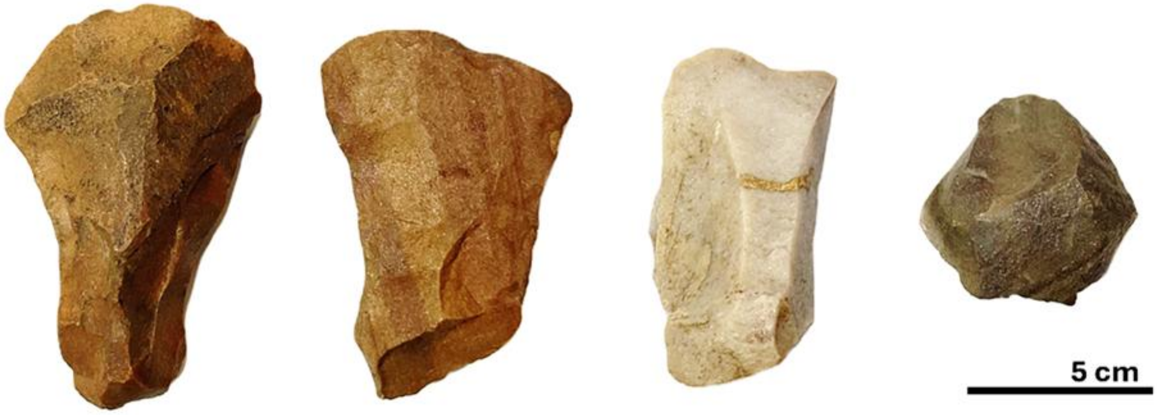

Fig. S16. GO-JA-01, lithic
Lithic tools. Photo Credit: Erica Rocha.

GO-JA-03 (Manoel Braga)
GO-JA-03 is an archaeological site (18°26'38.76"S 52° 0'1.78"W, state of Goiás, municipality of Serranópolis) located within a sandstone shelter in the Cerrado biome, at the base of a 64-meter-high rock massif. The sheltered area spans a maximum of 80 by 14 meters and features rock paintings and engravings. Excavations exposed 30 m² of the site, uncovering a sedimentary package that is thin near the shelter's wall (approximately 25 cm) and increases in thickness towards the drip line, reaching up to 350 cm (*76, 109*). The excavations were conducted by a team led by Dr. Pedro Ignacio Schmitz, between 1975 and 1999. The archaeological materials were classified into four occupation phases, three defined by large sedimentary packages and one with fewer remains. The oldest occupation, marked by unifacial lithic tools and projectile points, dates between 11,200 and 6,300 cal. BP (*76*) (Data S1), comprising layers 8, 9 and 10 (*108*). The second phase, characterized by debitage flakes with minimal retouching, begins around 6,300 cal. BP and is associated with layers 4, 5, 6 and 7, containing abundant charcoal (*110*). Silicified portions of the bedrock served as a source of raw material for lithic production in both phases (*108*). Without a clear stratigraphic boundary between the second and the third occupation, or absolute dates, ceramics of the Una tradition (Fig. S17) gradually appear from layer 3 to the surface, along with abundant plant remains, including *Arachis hypogaea* (peanut), *Zea mays* (maize), *Anacardium* sp. (cashew), *Annona* sp. (araticum), *Hymenaea courbaril* (jatobá), and various palm species (*108*). On the surface, Tupiguarani pottery was identified, characterizing the fourth and most recent layer of occupation (*108*). Two human burials very close to each other were discovered in the layers 1-3, associated with ceramics of the Una tradition, being one of a perinatal individual and another of an individual whose age was not estimated (*76*). In this study, we analyze the bone collagen of a rib from the second individual, probably an adult. Unfortunately, the sample did not have collagen. We also analyzed faunal samples from late Holocene layers of this site (Data S2). The material is part of the collection of the Pontifícia Universidade Católica de Goiás (Goiânia, GO).

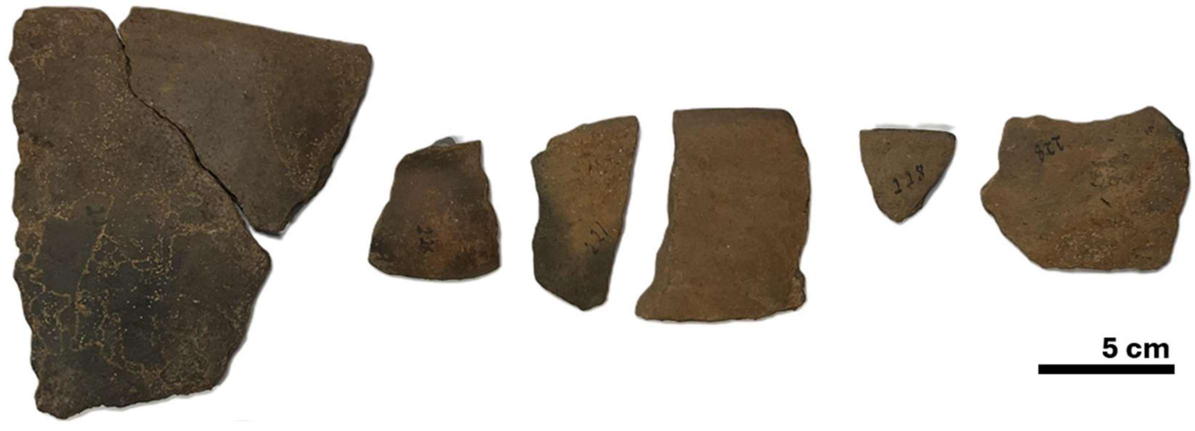

Fig. S17. GO-JA-03, pottery
Pottery fragments from the Una ceramic tradition. Photo Credit: Erica Rocha.

GO-RS-01 (Rasmusse Nóbrega)
GO-RS-01 (10°46'47.90"S 47°59'59.87"W, state of Tocantins, municipality of Monte do Carmo) is an archaeological site located within a sandstone shelter in the Cerrado biome. The sheltered area measures up to 40 by 14 meters, with 20 m² excavated. In the stratigraphic deposit, ranging between 80 and 130 cm thick, lithic materials, pottery (Fig. S18), and 10 human burials were excavated by Dr. Pedro Ignacio Schmitz and Dr. Altair Salles Barbosa between 1978 and 1982 (*26*). The pottery is simple and smoothed with a few fragments with red engobe, assigned to Una tradition (Fig. S18), although changes in temper over time were noted (*26*). Pottery fragments found in layers dated to ca. 4,500-4,000 cal. BP (Data S1) at the base of the deposit raise questions about their original context, with the possibility of bioturbation or anthropogenic displacement (*26*). More reliable radiocarbon dates for the layers with pottery suggest an age of at most 2,698-2,133 cal. BP (*29*) (Data S1), characterizing this as one of the oldest occurrences of the Una pottery. In this study, we analyzed a molar tooth and a femur fragment from Burial 3 and long bone fragments from Burials 1 and 8. Burial 1 was directly dated to 921-769 cal. BP (this work) (Data S1). We also analyzed faunal samples from this collection (Data S2). The material is part of the collection of the Pontifícia Universidade Católica de Goiás (Goiânia, GO).

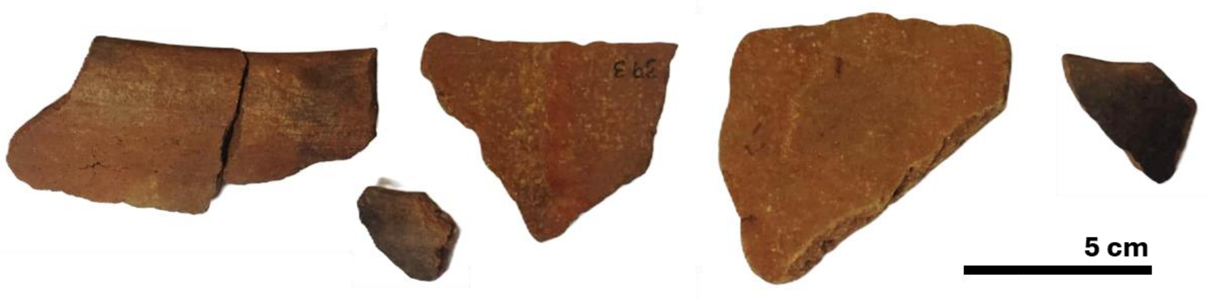

Fig. S18. GO-RS-01, pottery
Pottery fragments from the Una ceramic tradition. Photo Credit: Erica Rocha.

Gruta dos Milagres

Gruta dos Milagres (15°19'5.90"S 47°43'34.48"W, state of Goiás, municipality of Planaltina), located in the Cerrado biome, is an archaeological site situated within a limestone cave featuring two main halls at different levels. This site was also registered as GO-NI-003, GO-NI-011 e GO-CB-13. The site was excavated in 1983 as part of the Anhanguera Project, led by Dr. Margarida Andreatta. Excavations in an area of 4 m^2 recovered lithic artifacts, pottery, charcoal, and animal remains, including bones of anteater (*Myrmecophaga tridactyla*), deer (Cervidae) and lizard (*Tupinambis* sp.), analyzed here to construct a dietary baseline for comparison with human diet in the Cerrado (Data S2). The material is part of the collection of the Museu Antropológico of the Universidade Federal de Goiás (Goiânia, GO).

José Honório

José Honório (14°22'10"S 43°39'31"W, state of Bahia, municipality of Iuiú) is an open-air archaeological site located in the Caatinga-Cerrado ecotone, where several human burials were exposed by slow erosion processes. Three of these burials were excavated: the first in the 2000's by Dr. Joaquim Perfeito, from the Universidade Estadual do Sudoeste da Bahia (UESB), and the other two in 2022 by Luydy Fernandes, Maria Ana Correia, Andersen Liryo, Eliane Chim and Haruan Straioto. Urn 1 [2019] and Urn 1 [2022] were buried in Aratu tradition funerary urns, while Burial 2 was directly buried in the soil, with the body covered by two pottery pots also with Aratu features (Fig. S19). In this study, we analyzed a second molar tooth and a rib fragment from the individual of the Urn 1 [2019] and long bone fragments from individuals of Urn 1 [2022] and Burial 2. Two human skeletons were directly dated to 505-333 cal. BP and 495-350 cal. BP (this work) (Data S1). These surprisingly late dates suggest the continuity of Aratu sites after the European invasion, challenging the current hypothesis that large Aratu villages disappeared due to conflicts with other societies at 590 BP, before the arrival of Europeans. The material is part of the collection of the Universidade Federal do Recôncavo da Bahia (Cachoeira, BA).

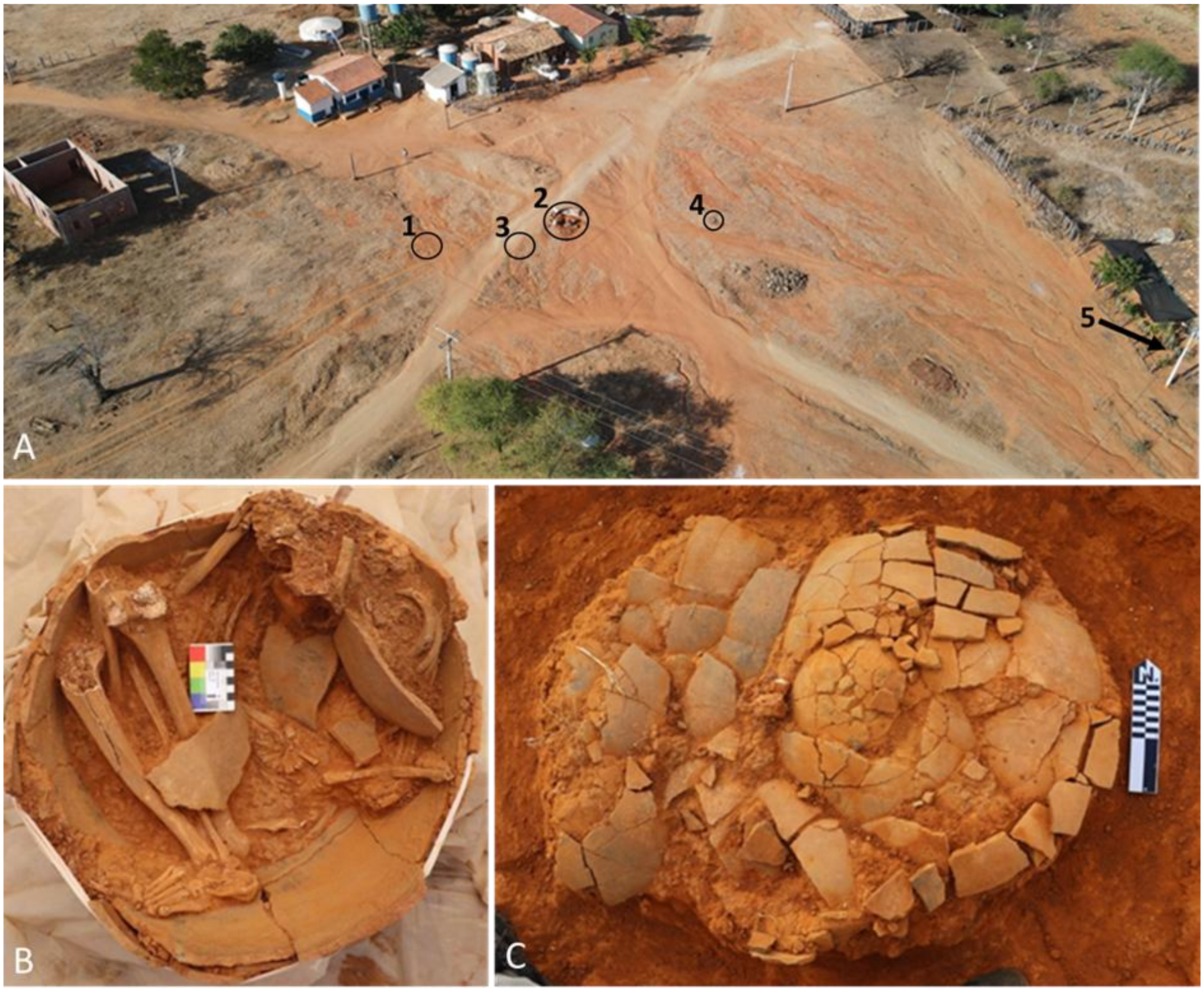

Fig. S19. José Honório

A) Aerial view, note de erosive process. The number correspond to human burials identified in the 2022 field work. B) Urn 1 during excavation; C) Burial 2 in situ, two pottery pots fragmented covering the human bones. Photo Credit: Haruan Straioto (A), Andersen Liryo (B) and Luydy Fernandes (C).

Lamarão

During maintenance work on a rural road in the Lamarão municipality (11°48'16.34"S 38°53'59.33"W, state of Bahia), situated in the ecotone between the Cerrado and Caatinga biomes, an Aratu funerary urn was accidentally discovered (Fig. S20). The operation of the road maintenance machinery damaged the urn, exposing the human bones inside. The only archaeological activity conducted at the site was the rescue of the funerary urn in 2020 by Dr. Luydy Fernandes. Here we analyzed a third molar and a long bone fragment of this individual, dated to 631-541 cal. BP (this work) (Data S1). The material is part of the collection of the Universidade Federal do Recôncavo da Bahia (Cachoeira, BA).

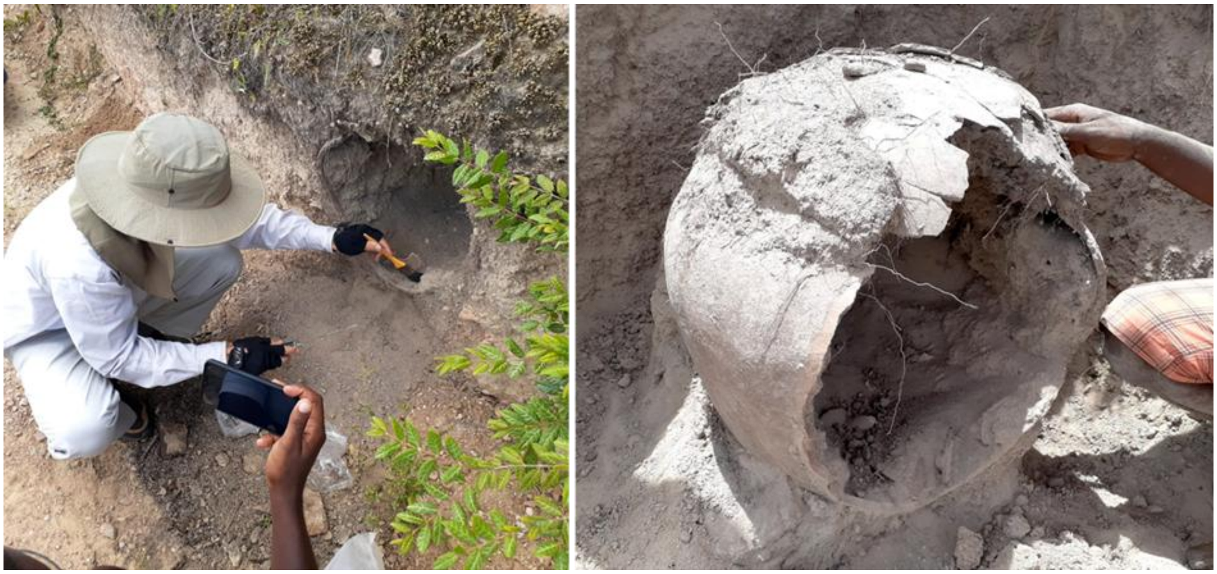

Fig. S20. Lamarão

Rescue of an Aratu funerary urn exposed during maintenance of a rural road. Photo Credit: Luydy Fernandes.

Lapa do Boquete

The Lapa do Boquete (Fig. S21) archaeological site (15° 6'14.04"S 44°14'14.66"W, state of Minas Gerais, municipality of Januária) lies in a limestone cave in the Cerrado biome, at the ecotone with the Caatinga. The site is situated in the Cavernas do Peruaçu National Park, an UNESCO World Heritage Site. Occupied throughout the Holocene, it has more than 50 radiocarbon dates ranging from ca. 14,000 to 500 cal. BP (*111*) (Data S1). Exceptional preservation conditions allowed the recovery of perishable materials, including vegetable fibers, strings, baskets ("silos"), seeds, fruits and leaves of domesticated and wild plants (*44, 112*) (Fig. S21). Faunal remains, and six human burials were also excavated (*81, 113*). Relative dating based on stratigraphy suggests these burials date from the middle to late Holocene (*81*). Unfortunately, these materials were destroyed in the fire that struck the Museum of Natural History and Botanical Garden of the Federal University of Minas Gerais in 2020. To continue studying this important site, our team has been conducting new field research there since 2021.

In previous research, stable carbon and nitrogen isotopic analysis of 11 human burials from Boquete and Abrigo do Malhador (a neighboring site), potentially dating back to the middle and late Holocene, revealed a predominant consumption of C_3 plants. The average $\delta^{13}C$ and $\delta^{15}N$ values are -21.6 ± 1.2‰ and 5.2 ± 1.9‰, respectively (including perinatal, subadult and adult individuals), suggesting a predominantly C_3 diet and low trophic level (*38*). Baseline values for $\delta^{13}C$ and $\delta^{15}N$ interpretation were established using bones from herbivores (Cervidae and Rodentia), omnivores (Dasypodidae), and one carnivore (*Panthera onca*) (*38*). Although this preliminary isotopic work has been done, we seek to expand the sample set by analyzing bone collagen $\delta^{13}C$ and $\delta^{15}N$ from one individual directly dated to 626-498 cal. BP (Burial 4) (*73*) (Data S1), associated with Una pottery tradition, as well as faunal (n = 16) remains (Data S2). Burial 4 was excavated in the 1990s, during the research project led by Dr. Andre Prous, while faunal remains were collected in the current project led by Eliane Chim and Dr. André Strauss.

The material excavated in the 21st century is housed at the Museu de Arqueologia e Etnologia of the Universidade de São Paulo (São Paulo, SP).

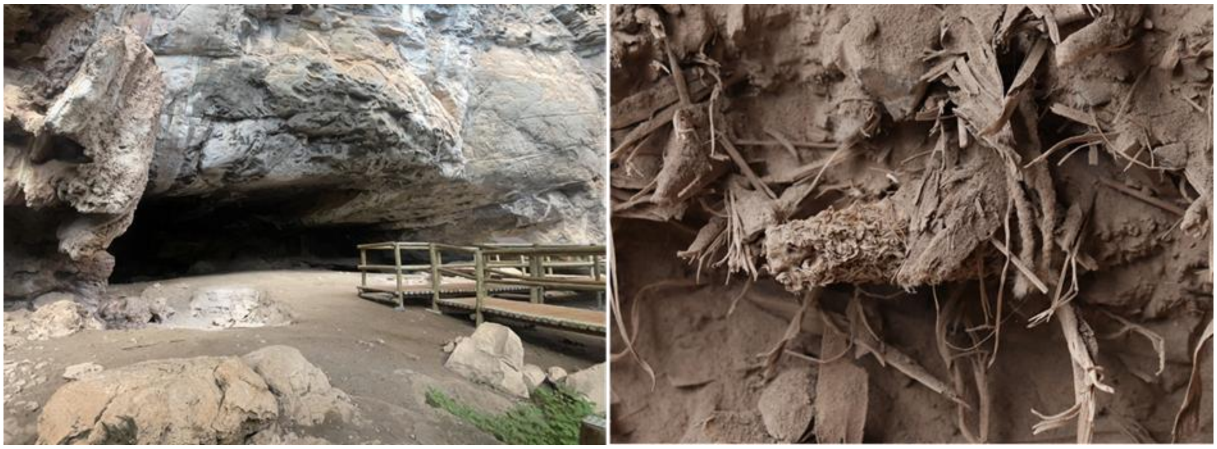

Fig. S21. Lapa do Boquete

View of the entrance to the archaeological site, opening to the south, and detail of and detail of plant basketry, with grass, a corn cob and a string, dated around 800 cal. BP. Photo Credit: Eliane Chim.

Lapa do Caboclo

Lapa do Caboclo is an archaeological site (18°17'7"S 43°51'5"W, state of Minas Gerais, municipality of Diamantina) located in a quartzite rock shelter in the Cerrado biome. Excavations led by Dr. Andrei Isnardis, in the 2000's, covered an area of 13 m², revealing stratigraphic deposits approximately 30 cm thick (*114*). These deposits contain two archaeological layers, dating from the early (12,689 to 11,934 cal. BP) and late (1,179 to 546 cal. BP) Holocene (Data S1). In the layer from the late Holocene, five secondary human burials, with at least six individuals, were excavated, along with features composed of botanical macroremains of *Manihot* sp., *Zea mays*, *Lagenaria* sp., cf. *Arachis* sp., and wild plants, such as *Hymenaea* sp. (*114*). Additionally, perishable materials such as wood, vegetable fibers, and leather were found (*115*). Lithic materials were present across all three layers, but no pottery fragments were identified (*114*). Here, we analyze a bone sample from one individual, the Burial 2. Burial 2 is a secondary burial, with the bones deposited in a cylindrical case made from tree bark. Resin or reddish wax was applied to several bones before burial, and the exceptional preservation of the remains allowed the identification of feather imprints on a femur and the skull. The individual was an adult male, estimated to be between 41 and 60 years old, according to bioarchaeological analysis (*115*). The material is part of the collection of the Museu de História Natural e Jardim Botânico of the Universidade Federal de Minas Gerais.

Lapa dos Desenhos

Lapa dos Desenhos (15° 6'29"S 44°14'1.34"W, state of Minas Gerais, municipality of Januária) is a large limestone rock shelter renowned for its extensive collection of over a thousand cave paintings, some reaching heights of up to 13 meters (Figs. S22 and S23). The site is situated in the Cavernas do Peruaçu National Park, an UNESCO World Heritage Site. Nearby archaeological sites, such as Lapa do Boquete, located just 1 kilometer away, show evidence of

continuous occupation throughout the Holocene (See Data S1). Surface findings at Desenhos site include perishable materials, lithic artifacts, and ceramic fragments of the Una tradition. Human bones and teeth were also discovered, likely displaced by a fossorial animal that had dug a burrow in the area. In this study, we analyzed a molar tooth and a long bone fragment from these materials, presumably associated with the most recent ceramic occupations. The materials were collected by Eliane Chim and Haruan Straioto in 2023 and is part of the collection of the Museu de Arqueologia e Etnologia of the Universidade de São Paulo (São Paulo, SP).

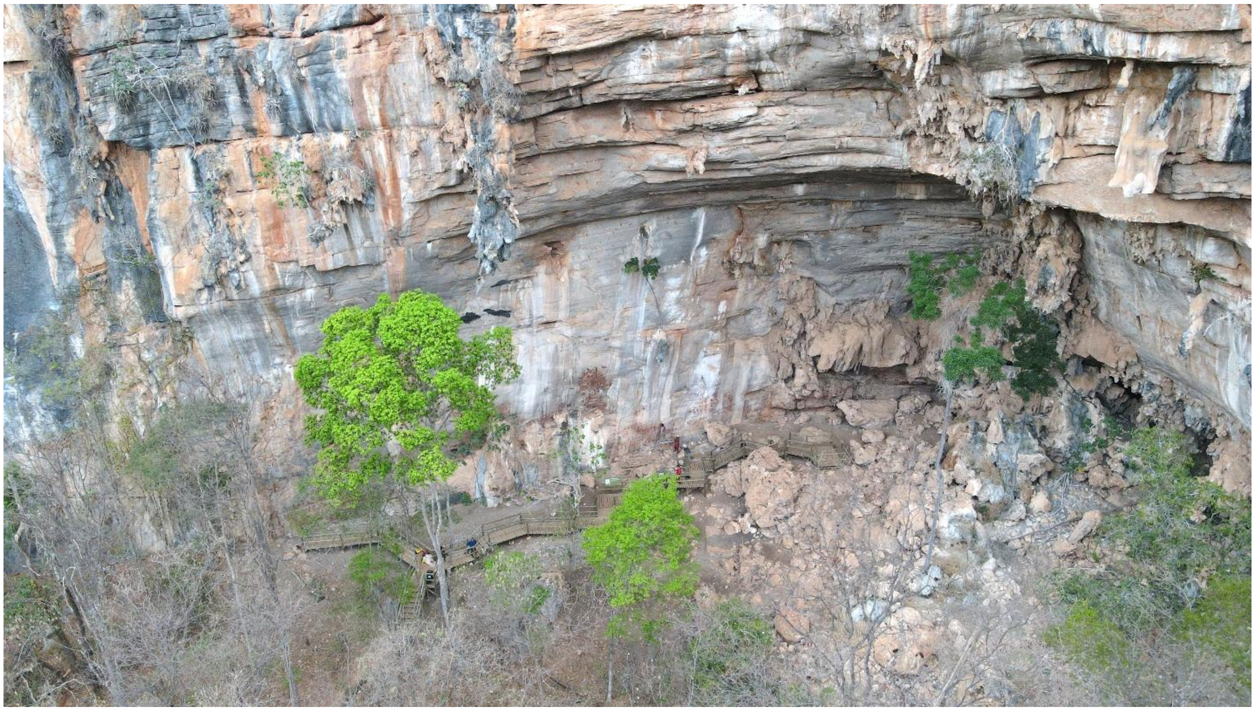

Fig. S22. Lapa dos Desenhos

Aerial view of the rock shelter. Photo Credit: Haruan Straioto.

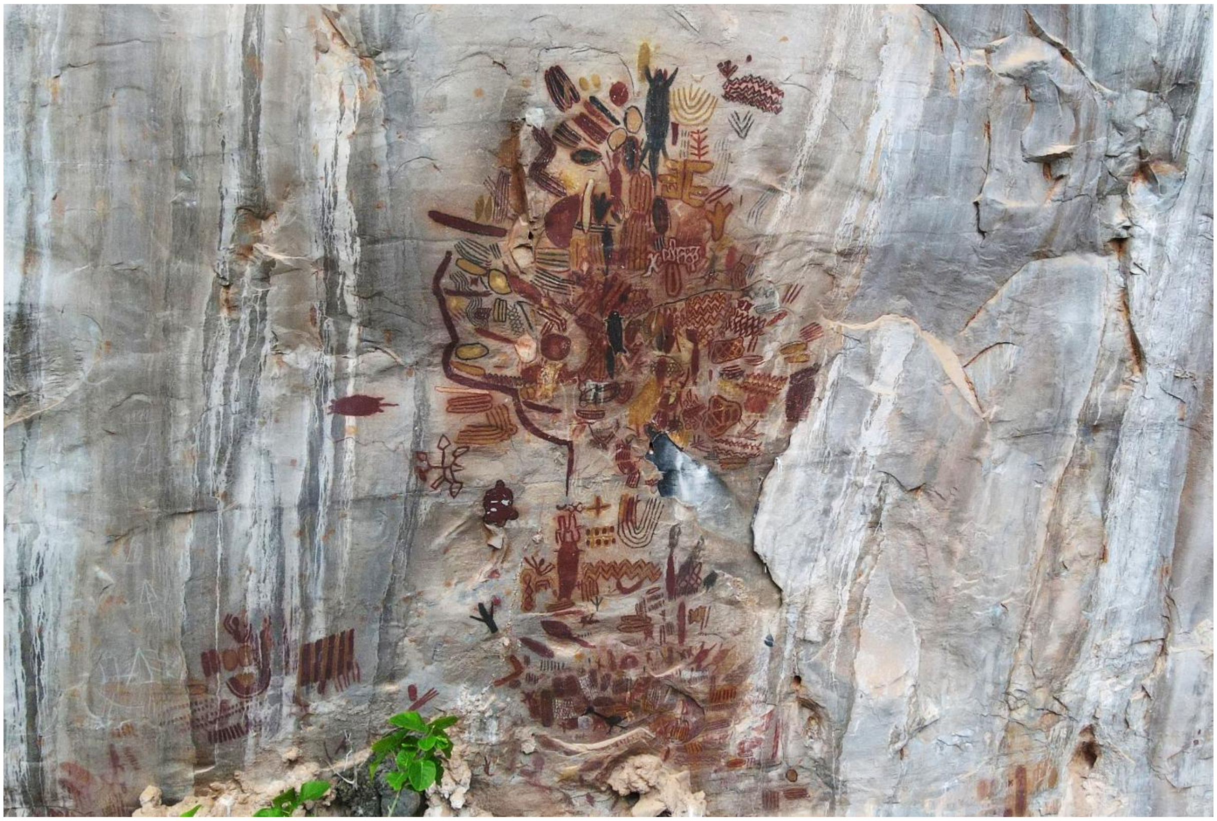

Fig. S23. Lapa dos Desenhos, rock art
Photo Credit: Haruan Straioto.

Lapa Pintada

Lapa Pintada (16°41'30.99"S 43°56'22.61"W, state of Minas Gerais, municipality of Montes Claros), located in the Cerrado biome, is a limestone shelter with semi-circular walls formed by fluvial erosion. The shelter features a hall measuring 40 meters in length, 25 meters in depth, and 50 meters in height, with an opening to the southeast (*116, 117*). In the 1950s, a local avocational archaeologist excavated the site due to the presence of rock paintings, unearthing about 14 skeletons and a large quantity of lithic material (*117*). Decades later, in the 21st century, Dr. Lucas Bueno re-excavated the site. In the most superficial layers, plant structures, artifacts made from perishable materials, and braided fabrics were identified, along with a few fragments of pottery from the Una tradition. Bottle gourd (*Lagenaria siceraria*) and manioc (cf. *Manihot esculenta*) were found in layers dated to 4,400 BP to 4,300 BP, while maize (*Zea mays*) was dated to 1,200 BP to 800 BP (*43*). An heir of the 1950s excavators later donated some human skeletal remains, primarily skulls and mandibles, to the Universidade de São Paulo. From these remains, three second molars and two third molars from five individuals were sampled and analyzed in this study. Human remains were dated to 761-465 cal. BP (this work) (Data S1). The material is part of the collection of the Museu de Arqueologia e Etnologia of the Universidade de São Paulo (São Paulo, SP).

Loca

The Loca archaeological site (7°20'36.13"S 35°50'22.72"W, state of Paraíba, municipality of Queimadas) is located within a rock shelter in the Caatinga biome. Over the years, it has suffered significant disturbances due to the use of dynamite for rock extraction and unauthorized excavations by local residents seeking human remains (*118*). The only professional archaeological investigation at the site was carried out by Dr. Juvandi Santos in 2009, during which human bones and pottery sherds were collected. The ceramics display characteristics typical of the Sertão region. Unfortunately, due to the extent of the damage and the lack of stratigraphic integrity, it was not possible to estimate the minimum number of individuals originally interred at the site. In this study, we analyzed a metatarsal fragment of one individual. The material is part of the collection of the Museu de História Natural of the Universidade Estadual da Paraíba (Campina Grande, PB).

Loca da Caveira

The Loca da Caveira archaeological site (7°8'54.52"S 36°1'10.82"W, state of Paraíba, municipality of Pocinhos) is located in Serra da Raposa, within the Caatinga biome. The site has been severely impacted by treasure hunters, who disturbed the sedimentary layers while searching for precious metals. The only professional archaeological research conducted there took place in 2010, led by Dr. Juvandi Santos. Human skeletal remains were recovered, however it was not possible to establish anatomical connections between the elements due to the extent of the disturbances. In this study, we analyzed a third molar and a phalanx fragment, although it is not certain whether they belong to the same individual. The material is part of the collection of the Museu de História Natural of the Universidade Estadual da Paraíba (Campina Grande, PB).

Malfazido cave

Malfazido (24°39'39.86"S 49°32'20.63"W, state of Paraná, municipality of Doutor Ulysses) is a limestone cave located in the southernmost part of the Cerrado biome, in the ecotone with Atlantic Forest. During research activities in speleology and paleoclimate, coordinated by Dr. Nicolás Strikis in 2021, a fragment of a human mandible was discovered on the surface at the entrance of the aphotic zone (Fig. S24). This was the only archaeological finding, and no excavations were conducted at the site. It appears that the mandible was transported by water flow. The mandible, which still contained three molar teeth in the alveoli, showed signs of post-depositional processes, including the loss of two premolar teeth and one canine tooth, as well as the precipitation of calcium carbonate on its surface. The presence of the third molar indicates that the individual was an adult. In this study we analyzed the third molar and a mandible fragment. The material is dated to 1,055-924 cal. BP (this work) (Data S1) and is part of the collection of the Museu de Arqueologia e Etnologia of the Universidade de São Paulo (São Paulo, SP).

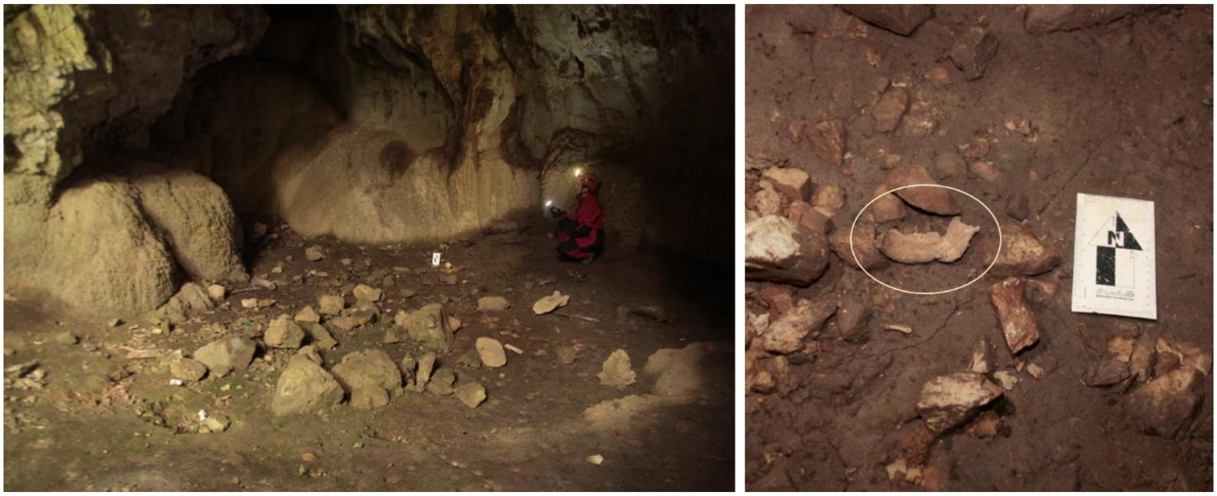

Fig. S24. Malfazido cave

Overall view of the entrance of the cave; human mandible found in the entrance of the cave. Photo Credit: Nicolás Strikis.

<u>Pambu</u>

In the Pambu locality (13°20'31.90"S 43°28'38.51"W, state of Bahia, municipality of Serra do Ramalho), situated in the Cerrado biome at the ecotone with the Caatinga, several archaeological sites were discovered by local residents. These sites contain patches of dark soil, ceramic fragments, stone tools, and shells from Bivalvia and Gastropoda. In 2017, during the digging of a garbage pit, residents uncovered an Aratu ceramic tradition funerary urn (Fig. S25). The burial differed slightly from the Aratu strictu sensu, as it represented a secondary burial. The only formal archaeological activity in the area was the documentation of the materials and the rescue of the urn, carried out by Dr. Luydy Fernandes. In this study we analyzed a second molar tooth and a long bone fragment from this individual dated to 521-494 cal. BP. The material is part of the collection of the Universidade Federal do Recôncavo da Bahia (Cachoeira, BA).

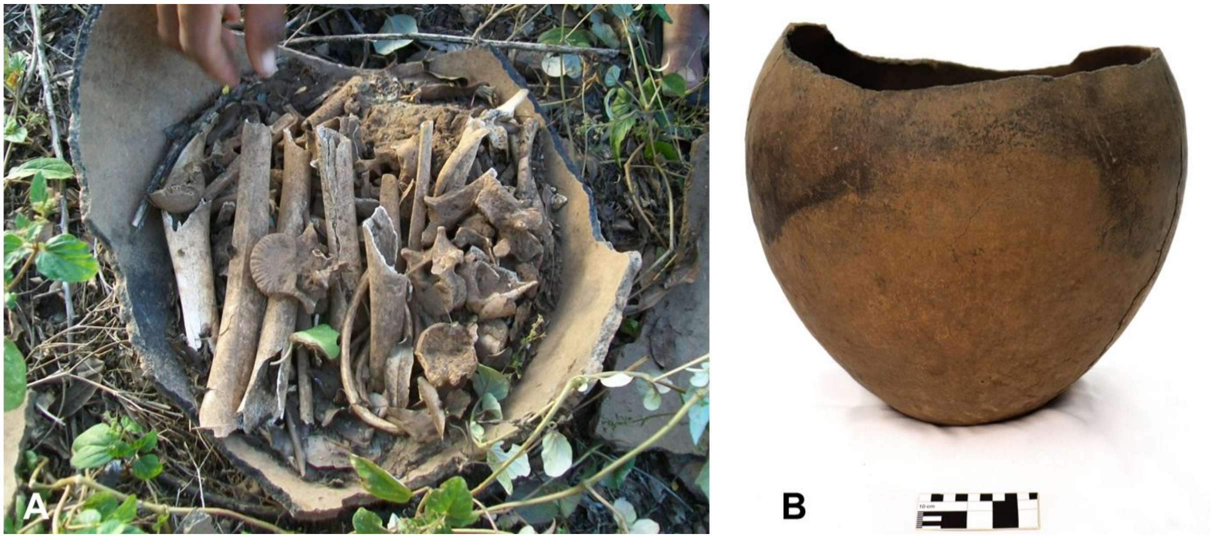

Fig. S25. Pambu

Human bones in the bottom of funerary urn excavated by local people and the reconstructed urn. Photo Credit: Henry Luydy Fernandes (A) and Mario Spock Fernandez (B).

Paulo Carapina (CT06E)

Paulo Carapina (18° 2'8.39"S 47°40'38.89"W, state of Goiás, municipality of Catalão) is an open-air archaeological site located in the Cerrado biome. Archaeological remains were dispersed over an area measuring approximately 300 by 200 meters and included lithic tools and hundreds of pottery sherds, among them twinned vessels, ceramic appendaged, and pear-shaped funerary urns. The presence of clay lumps was also recorded, interpreted as evidence of in situ ceramic production. Two funerary urns, spaced one meter apart, were excavated. The first urn was between 1.20 and 1.90 metres from the current ground surface, while the second was located between 0.80 and 1.20 meters. Unfortunately, the human skeletal remains were poorly preserved and were recovered only from Urn 2, represented by human teeth. In this study, we analyzed a tooth enamel sample from this individual, registered as Urn 2 (3859). The site was excavated by Fundação Aroeira during archaeological research conducted as part of environmental licensing prior to the construction of the Serra do Facão hydropower plant in 2011 (*119*). The material is part of the collection of the Pontifícia Universidade Católica de Goiás (Goiânia, GO).

Pinturas I

Pinturas I (8° 8'14.60"S 36°48'55.30"W, state of Paraíba, municipality of São João do Tigre) is an archaeological site in a rock shelter located at the top of a granite rock massif in the Caatinga biome (Fig. S26). The site was named after its red pigment cave paintings (Fig. S26), which attracted curious visitors and led to unauthorized excavations that exposed hundreds of human bones. A formal archaeological excavation covering 4 m² was later conducted by Dr. Juvandi Santos in 2008. The excavation reached a depth of up to 70 cm, with human bones, ceramics and stone tools being collected (*120*). Unfortunately, the human bones of at least 35 individuals were scattered throughout the area, making it impossible to collect complete skeletons or identify the burials (*120*). Here we analyzed three bone samples from this site, of which only one yielded collagen of sufficient quality. The material is part of the collection of the Museu de História Natural of the Universidade Estadual da Paraíba (Campina Grande, PB).

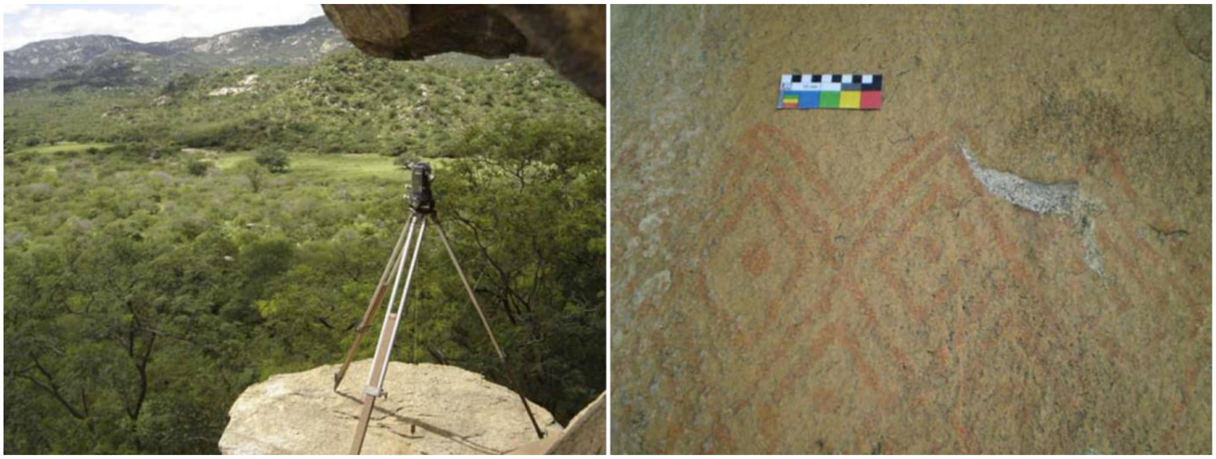

Fig. S26. Pinturas I

View of the landscape from the opening of the rock shelter and rock art identified in that place. Photo Credit: Juvandi de Souza Santos.

Praça de Piragiba

The archaeological site of Praça de Piragiba (12°11'57.86"S 43°49'51.53"W, state of Bahia, municipality of Muquém do São Francisco) (Fig. S27), located in the Cerrado biome at the ecotone with the Caatinga, contain one of the largest late Holocene burial grounds in pre-colonial Brazil. The site also comprised an Aratu ceramic tradition village, whose archaeological remains were mostly washed away by fluvial and pluvial erosion, preserving essentially the funerary urns. More than 120 urns were identified at the site, of which dozens were exhumed by Dr. Carlos Etchevarne and Dr. Henry Luydy Fernandes in the 1990's and 2000's (*84*). Based on ^{14}C collagen dates, the site was used as a burial ground between 1,043 and 664 cal. BP (*121*; this work) (Data S1). In this study, we analyzed samples from 13 individuals. From 10 of them, molar teeth and bone samples were examined, while from one only a molar tooth was analyzed, and from two only a bone sample. The material is part of the collections of the Museu de Arqueologia e Etnologia of the Universidade Federal da Bahia (UFBA) and of the Universidade Federal do Recôncavo da Bahia (Cachoeira, BA).

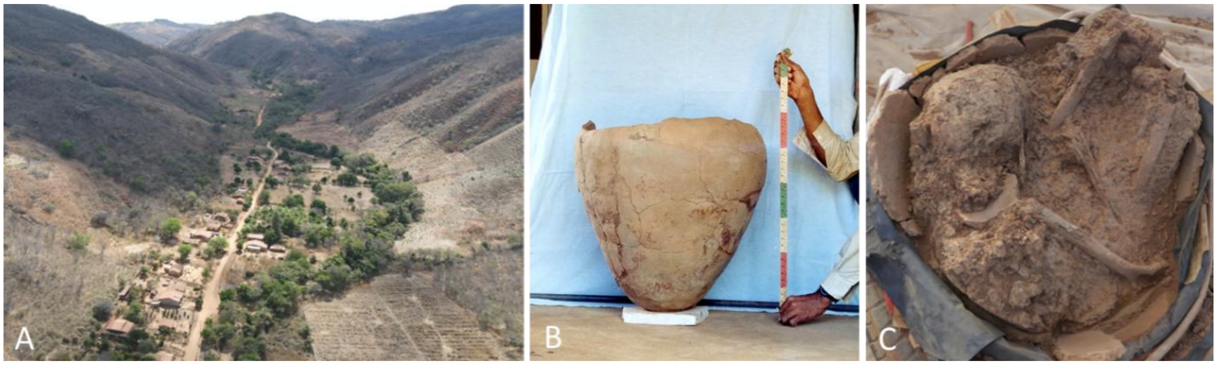

Fig. S27. Praça de Piragiba

A) Aerial view of the Piragiba modern-day village, where the site is located, in the floodplain of a stream; B) funerary urn of the Aratu ceramic tradition; C) excavation of Burial Un12Ur8. Photo Credit: Haruan Straioto (A); Luydy Fernandes (B and C).

Reitoria

In the Atlantic Forest biome, an Aratu funerary urn was found on Cruz das Almas campus of the Federal University of Recôncavo da Bahia (12°38'56.50"S 39° 5'10.76"W, state of Bahia, municipality of Cruz das Almas). Local residents have reported finding about 10 funerary urns at the site over the past 40 years, although only one has been excavated by archaeologists (Fig. S28), Dr. Luydy Fernandes, in 2020. In addition to ceramics, chipped and polished stone tools have also been identified, and several polishing channels are present along the banks of the stream adjacent to the site. Here we analyzed a long bone fragment from the urn excavated by local residents and sampled by Dr. Fernandes. The material is part of the collection of the Universidade Federal do Recôncavo da Bahia (Cachoeira, BA).

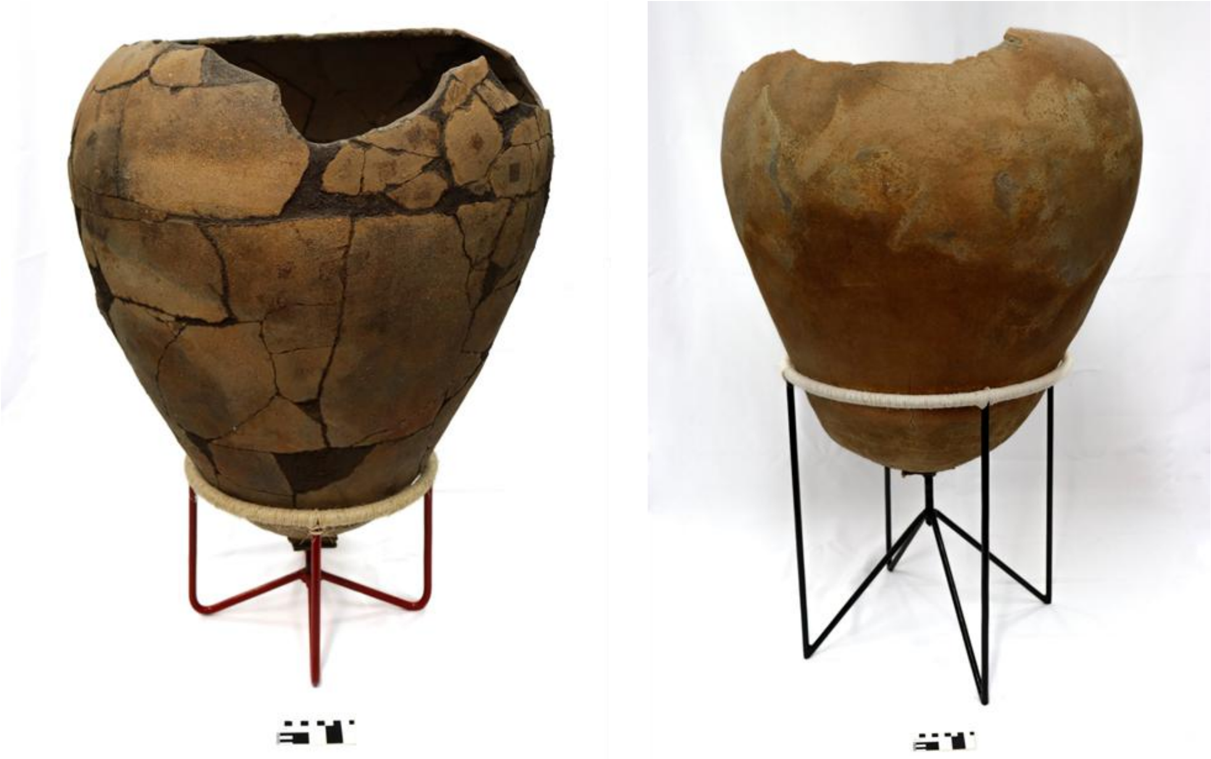

Fig. S28. Reitoria

Aratu funerary urns. Photo Credit: Mario Spock Fernandez.

<u>Serrote dos Ossos</u>

In a base of a granite block in the Caatinga biome, Serrote dos Ossos (7°49'39.90"S 36°30'9.90"W, state of Paraíba, municipality Caraúbas) is a small rock shelter measuring 4 by 3.5 meters, with a height of 1.60 meters at the opening (Fig. S29) (*122*). The site features rock paintings in red pigments depicting zoomorphic and anthropomorphic figures (*122*). Artifacts found at the site include necklace beads, pendants, plant fibers, ceramics, and shells, both on the surface and within the sedimentary context (*123*). Human bones from at least six individuals were scattered throughout the area, making it impossible to recover complete individualized burials (*123*). One bone fragment was radiocarbon dated by 1,059-932 cal. BP (*123*) (Data S1). In this study, we analyzed a molar tooth and a metacarpal from one individual and two molar teeth from two different individuals, as well as a bone sample from one metacarpal that may or not belong to the same two individuals. The material was excavated by Dr. Juvandi Santos in 2022 and is part of the collection of the Museu de História Natural of the Universidade Estadual da Paraíba (Campina Grande, PB).

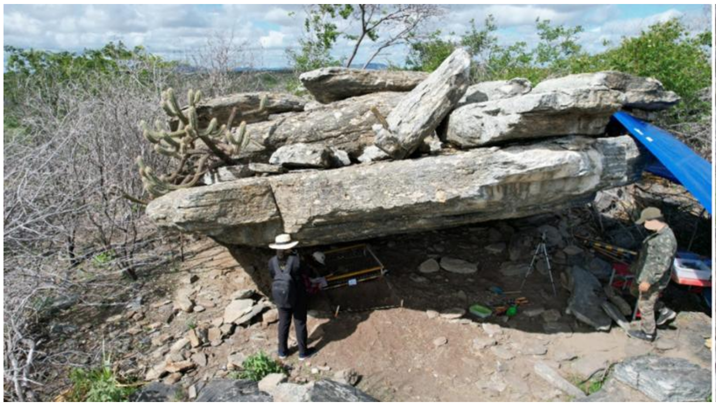
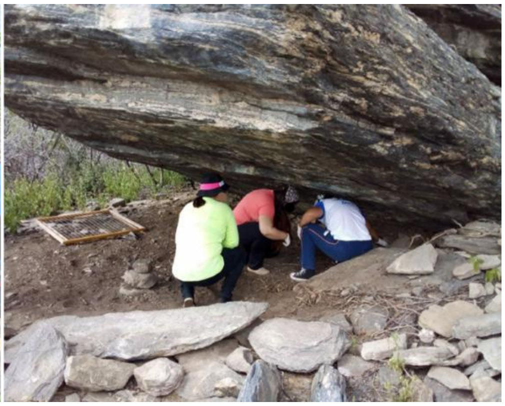

Fig. S29. Serrote dos Ossos

Overall view of the rockshelter and archaeological excavations. Photo Credit: Juvandi Santos.

Vale dos Sonhos

Vale do Sonhos (16°36'2.06"S 49°12'10.04"W, state of Goiás, municipality of Goiânia) is a village site in the Cerrado biome, covering an area of 320 m². Archaeological excavations at the site revealed thousands of pottery fragments from the Aratu ceramic tradition, as well as a polished axe. A single funerary urn (Fig. S30), containing only a few preserved bones and a tooth from one individual, was excavated at the site by Mariza de Oliveira Barbosa, Paulo Jobim and Sibeli Viana in 2002 (*124, 125*). Here, we analyzed a molar tooth and a femur fragment from this individual. The material is part of the collection of the Pontifícia Universidade Católica de Goiás (Goiânia, GO).

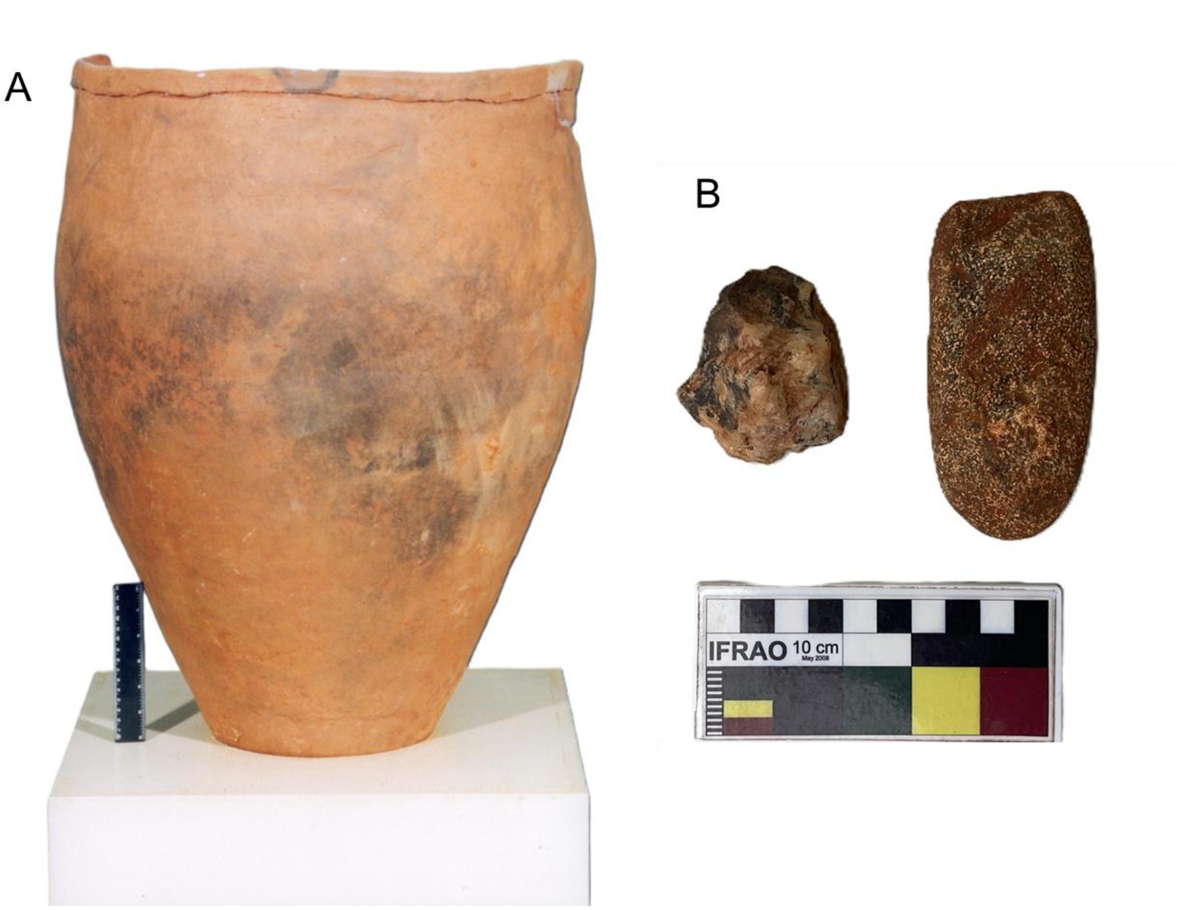

Fig. S30. Vale dos Sonhos

A) Funerary urn from Burial 1; B) Chipped and polished lithic tools. Photo Credit: Sibeli A. Viana (A) and Erica Rocha (B).

Vale Verde 1

The Vale Verde 1 archaeological site (12°37'31.57"S 43°19'56.16"W, state of Bahia, municipality of Sítio do Mato) is a village located in the Cerrado biome at the ecotone with Caatinga, on the left bank of the São Francisco River. Archaeological materials are dispersed in an area covering approximately 260 by 110 meters. The site was discovered by locals, in the rural area, when they found two funerary urns. Archaeological excavations conducted by Dr. Luydy Fernandes in 2011 revealed an archaeological sediment layer about 30 cm thick, within which five funerary urns were discovered (Fig. S31), along with chipped and polished axe blades and a spindle whorl (*126*). The site is dated to 673-567 cal. BP (this work) (Data S1). Burial 1 contained the only adult individual, while the remaining four urns held subadults. The vessels exhibit the typical morphology of the Aratu tradition, featuring smooth, undecorated surfaces. 'Opérculos' (lids) were identified on urns 4 and 5. Some burials also included beads made from small animal bones and shells. Here we analyzed a third molar and a metacarpal fragment from Burial 1, as well as long bone fragments from Burials 3, 4, and 5. The material is part of the collection of the Universidade Federal do Recôncavo da Bahia (Cachoeira, BA).

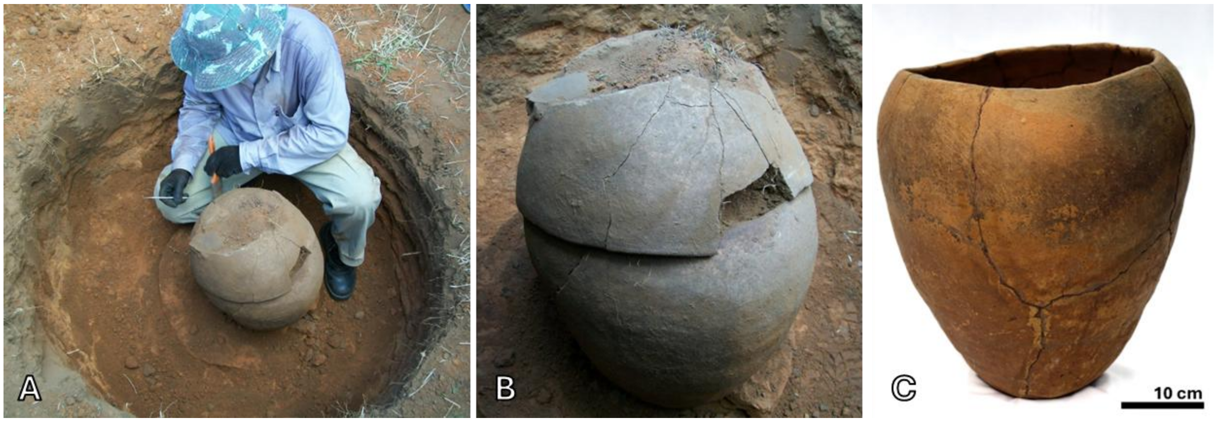

Fig. S31. Vale Verde 1

Funerary urns. Photo Credit: Luydy Fernandes (A, B) and Mario Spock Fernandez (C).

Várzea do Araticum

During maintenance work on a rural road in Várzea do Araticum (11°46'12"S 38°22'39"W, state of Bahia, municipality of Inhambupe), in the Atlantic Forest biome, an Aratu tradition funerary urn was accidentally discovered (Fig. S32). The only formal archaeological activity at this place was the documentation of the site and the rescue of the funerary urn by Dr. Luydy Fernandes in 2020. On the surface, abundant lithic raw material suitable for knapping, including chert nodules and silicified sandstone pebbles, were observed. Additionally, a road cut revealed a 40 cm thick occupation layer containing stone tools and ceramic fragments. The human skeletal remains, as well as the urn and its lid were well preserved. In this study, we analyzed a humerus fragment of this individual, dated to 724-662 cal. BP (this work) (Data S1). The material is part of the collection of the Universidade Federal do Recôncavo da Bahia (Cachoeira, BA).

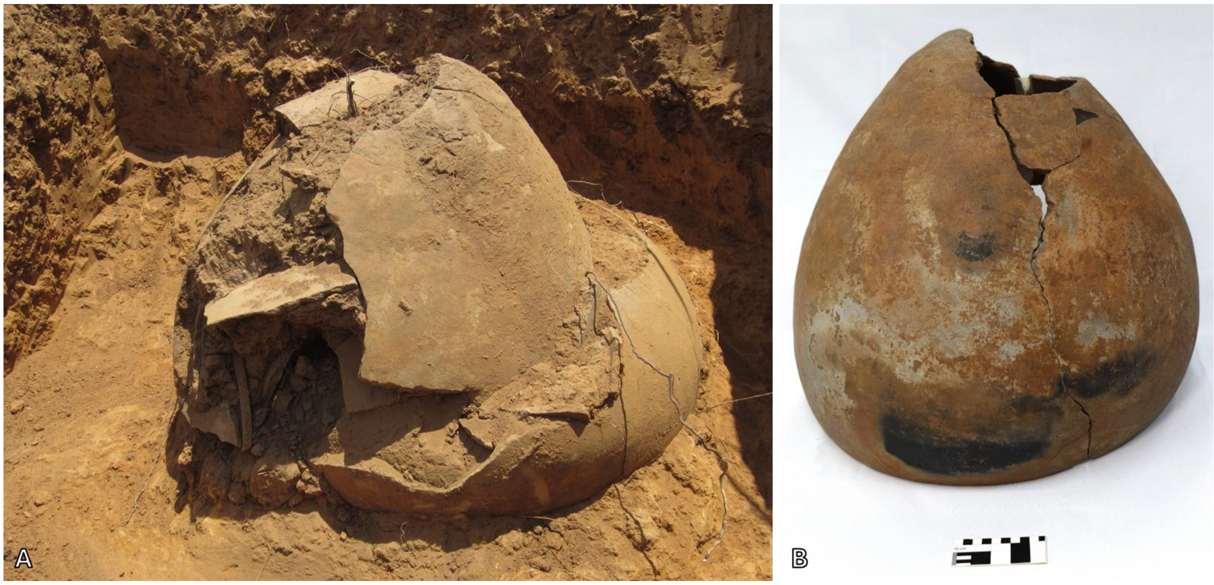

Fig. S32. Várzea do Araticum

A) excavation of funerary urn of burial 1; B) 'opérculo' of the funerary urn. Photo Credit: Luydy Fernandes (A) and Mario Spock Fernandez (B).

Vargem Formosa

The Vargem Formosa (MG00697) is an open-air archaeological site (19°30'0"S 46°20'0"W, state of Minas Gerais, municipality of Ibiá) located in the Cerrado biome. It is characterized by small concentrations of pottery fragments and a limited number of chipped and polished lithic materials. There is no noticeable difference in sediment color across the site. A notable discovery at this site is a human burial in an Aratu ceramic tradition funerary urn, from which a molar tooth has been analyzed in this study (*93*). There are no records of who excavated the material or when, however, it is part of the collection of the Museu de História Natural e Jardim Botânico of the Universidade Federal de Minas Gerais.

Vau I

Vau I (BA-RC-55) is an open-air archaeological site (13°13'36"S 44°35'27"W, state of Bahia, municipality of Santa Maria da Vitória) located in the Cerrado biome, within the São Francisco River basin. The site was accidentally discovered by the local community and later subjected to an emergency excavation in the 1990s, conducted by Dr. Altair Barbosa. During this process, ceramic vessels, faunal materials, and human burials were uncovered. The thin-walled, small, globular vessels without plastic or painted decoration (Fig. S33) found at the site were initially classified as Una tradition (*127*). However, these simple ceramics may have similarities with utilitarian artifacts of the Aratu tradition. Considering that this is an open-air site with funerary urns in a region where all other open-air sites belong to this tradition, in the present work this site will be considered to be part of the Aratu ceramic tradition. Unfortunately, after the excavation, the artifacts and human bones were displayed publicly before proper curation and inventory were completed, resulting in the loss of key contextual information about many of the pieces (*127*). Despite these challenges, analysis of the human bones whose origins could be confirmed through field documentation, identified at least 11 human burials (*127*), varying ages and both sexes, buried either directly in the soil or within ceramic urns (*89*). However, except for Burials 02 and

10, many bones, particularly skulls, mandibles, and teeth, were stored without proper information about their provenance. Additionally, about 30% of human bones have no contextual information (*127*). Consequently, we analyzed bone and tooth samples whose origins were clear and treated those without proper documentation as individual samples. In this study, we analyzed molar teeth and long bone fragments from two adult individuals, Burials 02 and 10, along with dental enamel from 10 loose second or third molars and collagen from 10 loose bones. Among these samples, a molar tooth from a skull, previously analyzed for ancient DNA (aDNA) and directly dated to 633-541 cal. BP (*89*), was also included. Overall, the site is dated from 633 to 517 cal. BP (Data S1). To construct a dietary baseline for comparison, we analyzed bone and tooth samples from species of deer (Cervidae), peccary (Tayassuidae), and wild cat (Felidae) found at the site (Data S2). The material is part of the collection of the Pontifícia Universidade Católica de Goiás (Goiânia, GO).

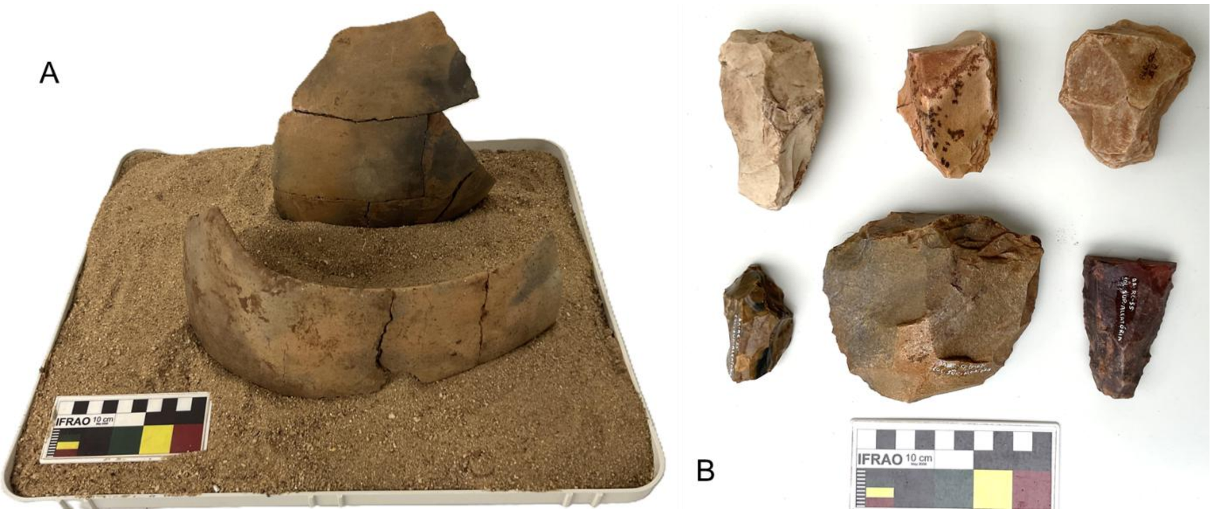

Fig. S33. Vau I

A) Ceramics of the Aratu tradition featuring thin-walled, small, globular vessels without plastic or painted decoration; B) Lithic tools. Photo Credit: Sibeli A. Viana (A) and Erica Rocha (B).

<u>Vau II</u>

Vau II (13°13'19.59"S 44°38'15.29"W, state of Bahia, municipality of Santa Maria da Vitória) is an archaeological site located 4 km from Vau I. At Vau II, three funerary urns of the Aratu tradition and lithic tools (Fig. S34) were excavated by Dr. Altair Salles Barbosa in the 1990s (*127*). In this study, we analyze a long bone fragment from Burial 1 and a molar tooth from Burial 3. The material is part of the collection of the Pontifícia Universidade Católica de Goiás (Goiânia, GO).

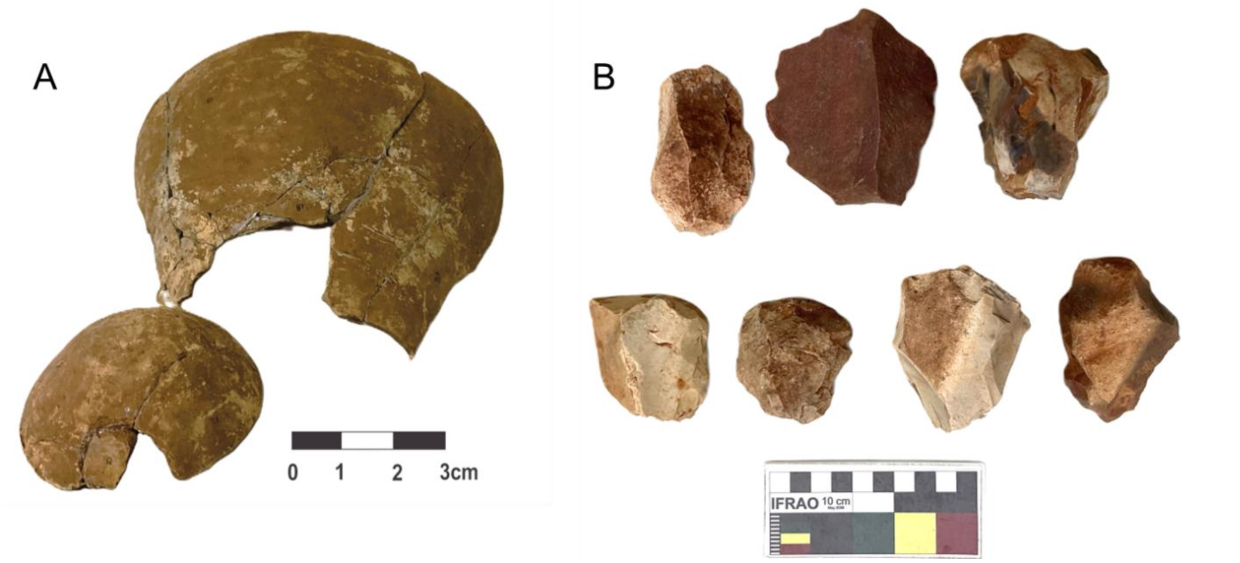

Fig. S34. Vau II

A) Ceramics of the Aratu tradition featuring thin-walled, small, globular vessels without plastic or painted decoration; B) Lithic tools. Photo Credit: Sibeli A. Viana (A) and Erica Rocha (B).

3. Stable isotopes

<u>Stable isotopes</u>

Stable carbon ($\delta^{13}C$) and nitrogen ($\delta^{15}N$) isotope analyses of human and animal skeletal remains offer valuable insights into the contribution of different resources to their diets (*51, 128–130*). This is possible because variation in $\delta^{13}C$ values reflects plant photosynthetic pathways in terrestrial ecosystems. C_3 plants (most trees, shrubs and herbs, temperate grasses and domesticates) are relatively depleted in ^{13}C, presenting lower $\delta^{13}C$ values (*131*). Meanwhile, C_4 plants (maize and most wild tropical grasses), are enriched due to less discrimination against ^{13}C, presenting higher $\delta^{13}C$ values (*131*). Crassulacean acid metabolism (CAM) plants resulting in $\delta^{13}C$ values that overlap or are intermediate to those of C_3 or C_4 plants (*132*). The 'canopy effect' further influences $\delta^{13}C$ variation within C_3 plants, with plants in dense forests being more depleted in ^{13}C compared to open environments (*129*). These distinctions are reflected in the tissues of consumers, providing information on their diet and environment (*36*). $\delta^{15}N$ measurements primarily reflect trophic levels (*130*), with an increase of ~2-6 ‰ observed between prey and consumers due to discrimination against ^{14}N during excretion (*51*). Environmental factors such as aridity and temperature also influence $\delta^{13}C$ and $\delta^{15}N$, necessitating regional baseline samples to best interpret datasets from humans (*133*). Tissue differences are also important for $\delta^{13}C$ interpretations. Bone collagen measurements primarily reflect the protein portion of the diet, potentially over-representing high-protein foods like meat and fish compared to low-protein resources like plants (*36*). In contrast, tooth enamel bioapatite $\delta^{13}C$ reflects the 'whole diet', including fats, protein, and carbohydrate (*37*). Additionally, tooth enamel analysis enables exploration of stable oxygen isotope ($\delta^{18}O$) variability, which primarily reflects the isotopic composition of imbibed water (*134*), which is linked to water temperature

and rainfall (*135, 136*). $\delta^{18}O$ analysis has been used to investigate mobility (*137*), paleoecology (*138*), and climate change (*139*).

Subsistence in late Holocene eastern South America

Since the 20th century, stable isotope analysis has been employed to assess the diet of pre-colonial human populations in South America (*46, 62, 140, 141*). However, it is only in recent decades that this method has been applied more widely to reconstruct diets during the Holocene in different parts of the South American continent. In the Amazon basin, analysis of stable carbon isotope measurements of human remains from Orinoco, Ucayali and Llanos de Mojos regions suggests maize had dietary significance in the last 1,200 years, as demonstrated by increasing consumption of C_4 plants (*46, 47, 141*). However, in Marajó Island, the data suggest a more diverse diet (*62*). Recent work has expanded this approach greatly within the Amazon Basin and isotopic analyses of human remains dated between 2,340 and 275 cal. BP have identified varied economic strategies, similar to those previously identified on Marajó. At the mouth of the Amazon River $\delta^{13}C$ and $\delta^{15}N$ values of bone collagen suggest that the diet was based on the exploitation of fish and a wide range of C_3 plant resources (*61*). The values of $\delta^{13}C$ and $\delta^{15}N$ of bone collagen recovered from the Amazon coast, meanwhile, indicate that C_3 plants and terrestrial mammals were more important for the diet than freshwater resources (*142*). Along the Xingu River, although the $\delta^{13}C$ and $\delta^{15}N$ values of bone collagen and $\delta^{13}C_{ap}$ suggest variability in individual diets, with some individuals consuming C_4 plants, the dataset points to the management of wild and cultivated plants combined with the exploitation of aquatic and terrestrial fauna resources (60). The available data thus suggests a large diversity of subsistence practices in the late Holocene pre-colonial Amazon, as well as supporting that forests have been intensively managed by indigenous populations for millennia (*7, 10, 13, 143, 144*).

In contrast to the Amazon, in eastern South America, most of the research on diet using stable isotopes has focused on assessing the coastal specialization of shell mound-building populations. Shell mound builders inhabited the Atlantic Forest of southeastern Brazil from around 11,000 to 5,000 cal. BP and the south and southeast coast from 8,000 to 700 cal. BP. Stable carbon and nitrogen isotope analyses of bone collagen indicated that terrestrial animals were the main source of protein for populations further away from the coast (*145*). The isotopic values of the coastal population revealed that the diet was based mainly on marine resources, with smaller contributions from terrestrial animals and C_3 plants (*140, 145–149*). Even after the appearance of cultivated plants in the coastal Atlantic Forest in the middle Holocene (*146, 150, 151*), marine resources remained a significant component of the diet, attesting to the long duration of coastal specialization (*145, 148, 149*). In one archaeological site along the southeast coast dated from 4,491 to 2,850 cal. BP a high reliance on marine food sources was identified as well as an important role of C_3 plants (*152*). Meanwhile, in other archaeological sites along the southern coast of Brazil, a trend to more terrestrial diets were identified after 1,000 cal. BP, albeit with a clear remaining dependence on marine food sources as well, even for pottery producing groups (*147, 153, 154*).

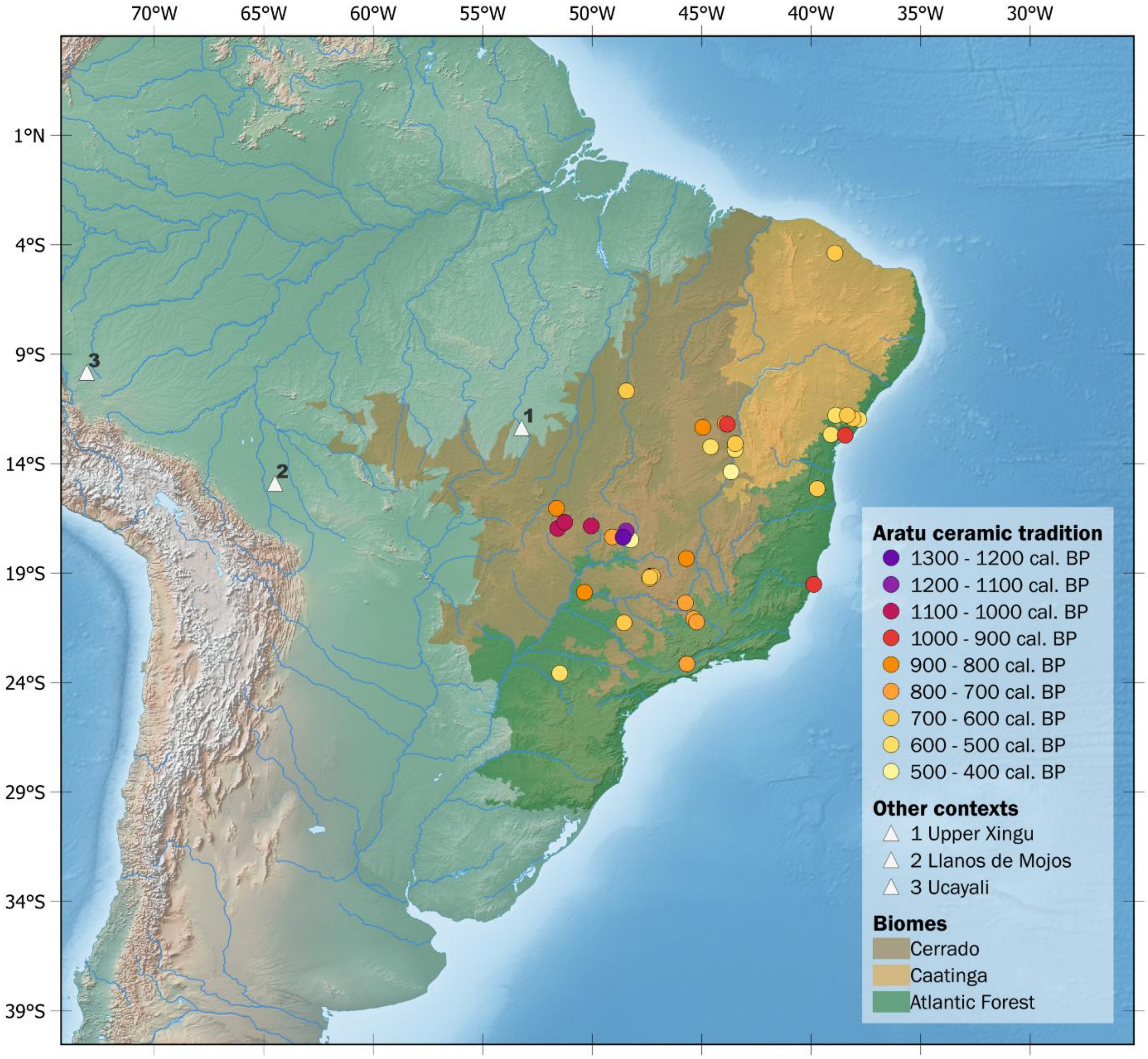

Fig. S35. Map showing biomes and archaeological sites of the Aratu ceramic tradition, as well as archaeological regions mentioned in the discussion

Aratu sites are shown according to radiocarbon dates of the Bayesian chronological modelling presented in Fig. S36. Complete data available in Data S1_C. Created for this study using QGIS 3.32.0 and Natural Earth.

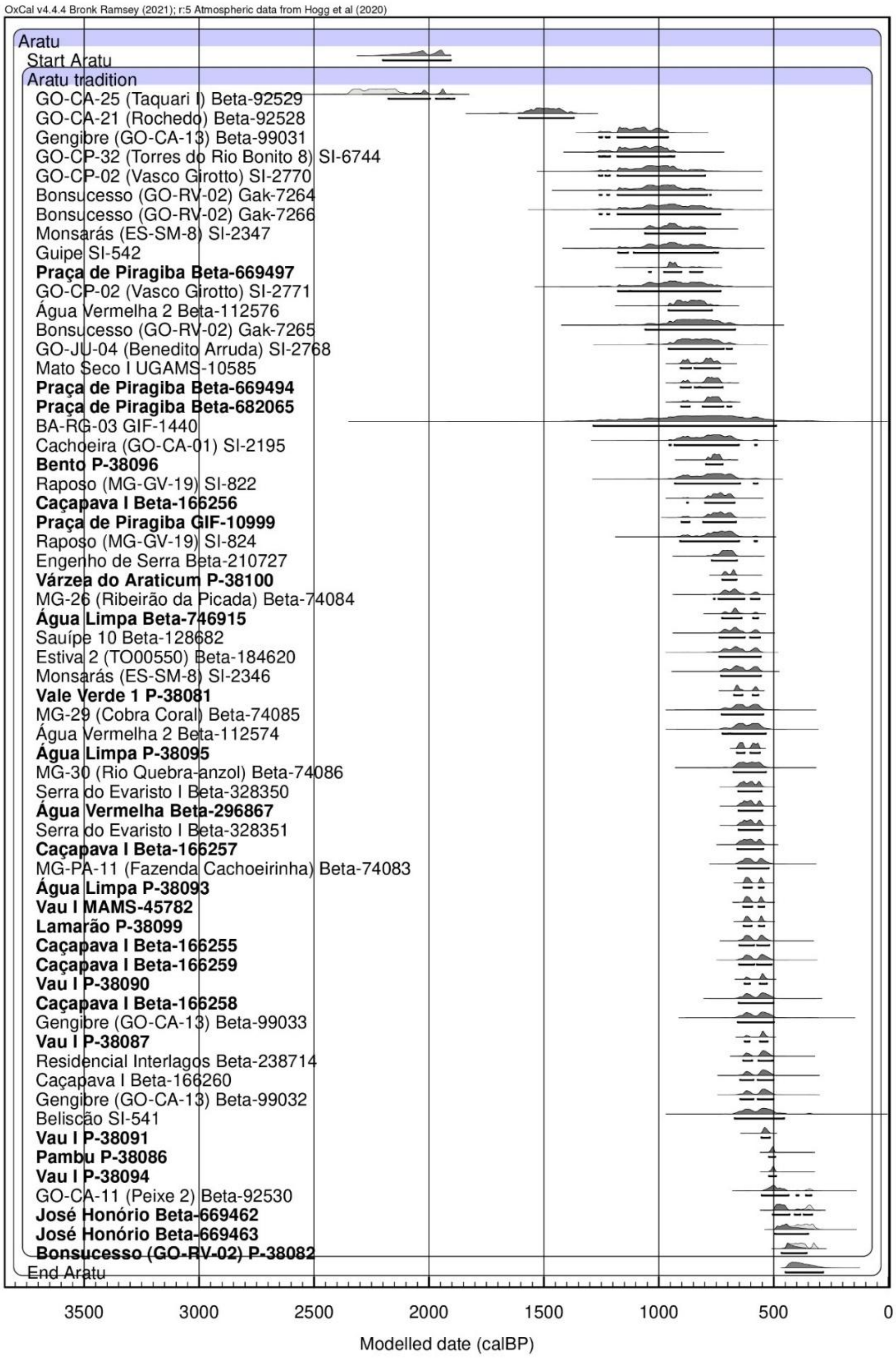

Fig. S36. Bayesian chronological modelling results for radiocarbon dates from charcoal and collagen of samples associated with the Aratu ceramic tradition

Collagen samples are shown in bold. Light grey represents unmodeled distributions of raw calibrated data, while dark grey represents modeled date ranges. Model agreement indices: Amodel = 74.5, Aoverall = 76. Complete data available in Data S1_C. Calibrations and modelling performed using OxCal 4.4.

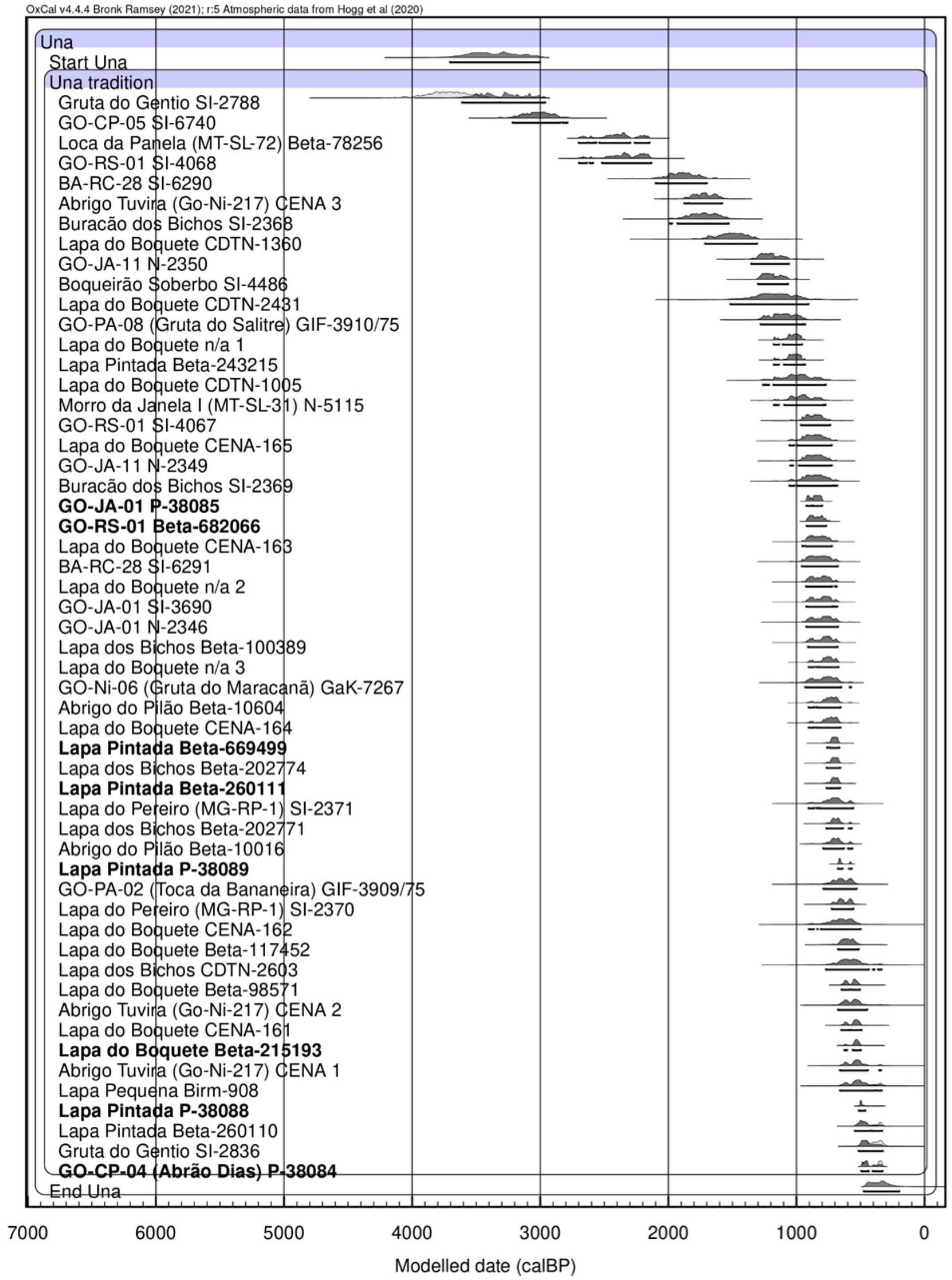

Fig. S37. Bayesian chronological modelling results for radiocarbon dates from charcoal and collagen of samples associated with the Una tradition

Collagen samples are shown in bold. Light grey represents unmodeled distributions of raw calibrated data, while dark grey represents modeled date ranges. Model agreement indices: Amodel = 85.3, Aoverall = 85.5. Complete data available in Data S1_E. Calibrations and modelling performed using OxCal 4.4.

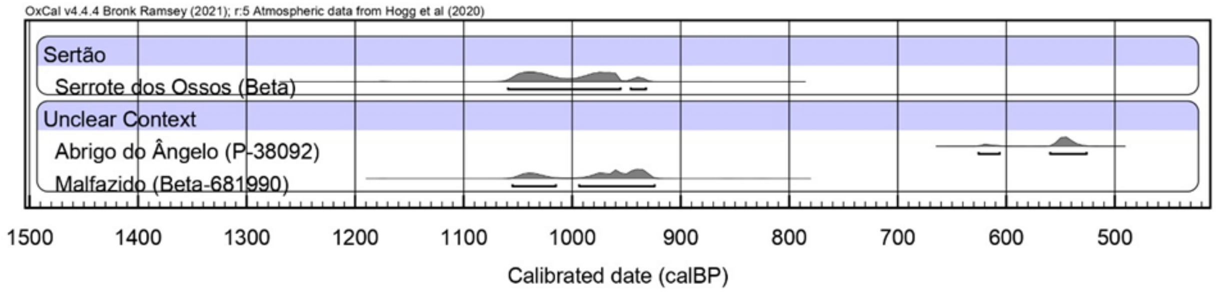

Fig. S38. Calibrated radiocarbon dates from collagen of sites from Caatinga (Sertão) and sites with unclear archaeological context

Complete data available in Data S1. Calibrations performed using OxCal 4.4.

Table S1.

Results of post-hoc Wilcoxon pair-wise comparison for $\delta^{13}C$ of fauna.

	**Carnivorous**	**Herbivorous**	**Myrmecophagous**
**Herbivorous**	0.71	-	-
**Myrmecophagous**	0.71	0.57	-
**Omnivorous**	0.57	0.71	0.43

Table S2.
Results of post-hoc Wilcoxon pair-wise comparison for $\delta^{15}N$ of fauna.

	**Carnivorous**	**Herbivorous**	**Myrmecophagous**
**Herbivorous**	0.60	-	-
**Myrmecophagous**	0.67	0.60	-
**Omnivorous**	0.60	0.60	0.60

Table S3.

Results of Shapiro Wilk normality test of $\delta^{13}C$ and $\delta^{15}N$ of humans from Aratu and Una tradition, and sertão pottery.

**Group**	**n**	**p_shapiro_d13Ccol**	**p_shapiro_d15N**
Aratu	49	0.326	0.0715
Una	12	0.259	0.281
Sertão	9	0.587	0.879

Table S4.

Results of Levene's variance test of $\delta^{13}C$ of humans from Aratu and Una tradition, and sertão pottery.

**Source**	**Degrees of freedom**	**F value**	**p-value**
Group	2	4.9644	0.009762
Residuals	67	-	-

Table S5.
Results of Welch's ANOVA statistical test of $\delta^{13}C$ of humans from Aratu and Una tradition, and sertão pottery.

**F**	**num df**	**denom df**	**p-value**
176.24	2	25.032	0

Table S6.

Results of Games-Howell post-hoc test of $\delta^{13}C$ of humans from Aratu and Una tradition, and sertão pottery.

Comparison	Estimate (mean diff)	95% CI (conf.low)	95% CI (conf.high)	p.adj	p.adj.signif
Aratu-sertão	-5.05	-5.70	-4.40	0	($p < 0.0001$)
Aratu-Una	-1.87	-3.47	-0.26	0.022	($p < 0.05$)
sertão-Una	3.18	1.61	4.75	0.000402	($p < 0.001$)

Table S7.

Results of Levene's variance test of $\delta^{15}N$ of humans from Aratu and Una tradition, and sertão pottery.

**Source**	**Degrees of freedom**	**F value**	**Pr(>F)**
Group	2	1.7488	0.1818
Residuals	67	-	-

Table S8.
Results of ANOVA statistical test of $\delta^{15}N$ of humans from Aratu and Una tradition, and sertão pottery.

	**Degrees of freedom**	**Sum of squares**	**Mean of squares**	**F value**	**Pr(>F)**
Group	2	9.66	4.832	2.252	0.113
Residuals	67	143.77	2.146	-	-

Table S9.

Results of Shapiro Wilk normality test of $\delta^{13}C$ and $\delta^{15}N$ of humans from Caatinga, Cerrado, and Atlantic Forest biomes.

Category	n	p_shapiro_d13Ccol	p_shapiro_d15N
Caatinga	9	0.587	0.879
Cerrado	59	0.0812	0.647
Atlantic Forest	4	0.751	0.193

Table S10.

Results of Levene's variance test of $\delta^{15}N$ of humans from Caatinga, Cerrado, and Atlantic Forest biomes.

**Source**	**Degrees of freedom**	**F value**	**Pr(>F)**
Group	2	2.2687	0.1111
Residuals	69	-	-

Table S11.

Results of ANOVA statistical test of $\delta^{15}N$ of humans from Caatinga, Cerrado, and Atlantic Forest biomes

	**Degrees of freedom**	**Sum of squares**	**Mean of squares**	**F value**	**Pr(>F)**
Biome	2	36.84	18.419	10.82	< 0.001
Residuals	69	117.50	1.703	-	-

Table S12.

Results of Tukey post-hoc pairwise test of $\delta^{15}N$ of humans from Caatinga, Cerrado, and Atlantic Forest biomes.

	**diff**	**lwr**	**upr**	**p adj**
Cerrado – Caatinga	-0.9891664	-2.10773715	0.1294043	0.0935565
Atlantic Forest – Caatinga	1.9218070	0.04345627	3.8001577	0.0437830
Atlantic Forest – Cerrado	2.9109734	1.29598082	4.5259659	0.0001516

Table S13.

Results of Shapiro Wilk normality test of $\delta^{13}C_{en}$ and $\delta^{18}O$ of humans from Aratu and Una traditions, sertão pottery and rock shelters with unclear cultural context

Category	n	p_shapiro_d13Cen	p_shapiro_d18O
Aratu	38	0.00641	0.000608
Una	8	0.845	0.205
Sertão	4	0.233	0.966
Unclear context (rock shelter)	3	0.965	0.487

Table S14.

Results of Levene's variance test of $\delta^{13}C_{en}$ of humans from Aratu and Una traditions, sertão pottery and rock shelters with unclear cultural context

Source	Degrees of freedom	F value	p-value
Group	3	1.5939	0.2028
Residuals	49	-	-

Table S15.

Results of Kruskal-Wallis test of $\delta^{13}C_{en}$ of humans from Aratu and Una traditions, sertão pottery and rock shelters with unclear cultural context

**Source**	**Chi-squared**	**Degrees of freedom**	**p-value**
Group	25.783	3	0.00001059

Table S16.

Results of Wilcoxon post hoc test of $\delta^{13}C_{en}$ of humans from Aratu and Una traditions, sertão pottery and rock shelters with unclear cultural context

	**Aratu**	**Sertão pottery**	**Una**
**Sertão pottery**	0.002	-	-
**Una**	0.0000086	0.596	-
**Unclear context (rock shelter)**	0.031	1.000	0.596

Table S17.
Number of human individuals analyzed per site

Site	Number of individuals analyzed
Abrigo do Ângelo	1
Abrigo Tuvira	1
Água Limpa	9
Água Vermelha	2
Alagamar	1
Antônio Beiraldo	1
Bento	3
Bonsucesso (Go-RV.2)	1
Buriti (GO-JU-54)	1
Camp. 1 (Camalaú)	1
Embiara	1
Furna dos Ossos	1
GO-CP-04 (Abraão Dias)	1
GO-JA-01	2
GO-JA-03	1
GO-RS-01	3
Jose Honorio	3
Lamarão	1
Lapa do Boquete	1
Lapa do Caboclo	1
Lapa dos Desenhos	1
Lapa Pintada	5
Loca	1
Loca da Caveira	2
Malfazido	1
Pambu	1
Paulo Carapina	1
Pinturas I	3
Piragiba	13
Reitoria	1
Serrote dos Ossos	5
Vale dos Sonhos	1
Vale Verde	4
Várzea do Araticum	1
Vargem Formosa	1
Vau I	22
Vau II	2

Supplementary Data files for this manuscript include the following:

Data S1 (separate file). Chronological data. Files include:

- Data S1_A – All dates from the analyzed archaeological sites, which were described in the Supplementary Text.
- Data S1_B – OxCal code for calibrating radiocarbon dates from the analyzed archaeological sites. Results presented in Data S1_A.
- Data S1_C – Radiocarbon dates from the Aratu ceramic tradition sites, including conventional ages, calibrated (unmodelled) dates, and Bayesian model results.
- Data S1_D – OxCal code for the Bayesian chronological model of the Aratu ceramic tradition. Results presented in Data S1_C.
- Data S1_E – Radiocarbon dates from the Una ceramic tradition sites, including conventional ages, calibrated (unmodelled) dates, and Bayesian model results.
- Data S1_F – OxCal code for the Bayesian chronological model of the Una ceramic tradition. Results presented in Data S1_E.

Data S2 (separate file). Results of stable isotope analyses ($\delta^{13}C_{en}$, $\delta^{18}O$, $\delta^{15}N$, $\delta^{13}C_{col}$):

- Data S2_A – Human individuals, including cultural and environmental information and identification of the analyzed tooth or bone.
- Data S2_B – Faunal samples, including site, taxonomic identification, common name, and the identification of the analyzed tooth or bone.

Data S3 (separate file). Published data used for the composition of Fig. 8.

Data S4 (separate file). R scripts used to generate Figs. 2–8.

REFERENCES

1. P. Roberts, C. Hunt, M. Arroyo-Kalin, D. Evans, N. Boivin, The deep human prehistory of global tropical forests and its relevance for modern conservation. *Nat. Plants* **3**, 17093 (2017).

2. N. L. Boivin, M. A. Zeder, D. Q. Fuller, A. Crowther, G. Larson, J. M. Erlandson, T. Denham, M. D. Petraglia, Ecological consequences of human niche construction: Examining long-term anthropogenic shaping of global species distributions. *Proc. Natl. Acad. Sci. U.S.A.* **113**, 6388–6396 (2016).

3. D. R. Piperno, A. J. Ranere, I. Holst, J. Iriarte, R. Dickau, Starch grain and phytolith evidence for early ninth millennium B.P. maize from the Central Balsas River Valley, Mexico. *Proc. Natl. Acad. Sci. U.S.A.* **106**, 5019–5024 (2009).

4. A. Roosevelt, *Subsistence in the Tropics: Parmana. Prehistoric Maize and Manioc Subsistence Along the Amazon and Orinoco* (Studies in Archaeology, Academic Press, 1980).

5. U. Lombardo, L. Hilbert, M. Bentley, C. Bronk Ramsey, K. Dudgeon, A. Gaitan-Roca, J. Iriarte, A. G. Mejía Ramón, S. Quezada, M. Raczka, J. G. Watling, E. Neves, F. Mayle, Maize monoculture supported pre-Columbian urbanism in southwestern Amazonia. *Nature* **639**, 119–123 (2025).

6. L. Kistler, S. Y. Maezumi, J. Gregorio de Souza, N. A. S. Przelomska, F. Malaquias Costa, O. Smith, H. Loiselle, J. Ramos-Madrigal, N. Wales, E. R. Ribeiro, R. R. Morrison, C. Grimaldo, A. P. Prous, B. Arriaza, M. T. P. Gilbert, F. de Oliveira Freitas, R. G. Allaby, Multiproxy evidence highlights a complex evolutionary legacy of maize in South America. *Science* **362**, 1309–1313 (2018).

7. S. Y. Maezumi, D. Alves, M. Robinson, J. G. de Souza, C. Levis, R. L. Barnett, E. Almeida de Oliveira, D. Urrego, D. Schaan, J. Iriarte, The legacy of 4,500 years of polyculture agroforestry in the eastern Amazon. *Nat. Plants* **4**, 540–547 (2018).

8. S. Y. Maezumi, S. Elliott, M. Robinson, C. J. Betancourt, J. Gregorio de Souza, D. Alves, M. Grosvenor, L. Hilbert, D. H. Urrego, W. D. Gosling, J. Iriarte, Legacies of Indigenous land use

and cultural burning in the Bolivian Amazon rainforest ecotone. *Philos. Trans. R. Soc. Lond. B Biol. Sci.* **377**, 20200499 (2022).

9. M. Arroyo-Kalin, The Amazonian formative: Crop domestication and anthropogenic soils. *Diversity* **2**, 473–504 (2010).

10. J. Watling, M. P. Shock, G. Z. Mongeló, F. O. Almeida, T. Kater, P. E. de Oliveira, E. G. Neves, Direct archaeological evidence for Southwestern Amazonia as an early plant domestication and food production centre. *PLOS ONE* **13**, e0199868 (2018).

11. J. Iriarte, S. Elliott, S. Y. Maezumi, D. Alves, R. Gonda, M. Robinson, J. Gregorio de Souza, J. Watling, J. Handley, The origins of Amazonian landscapes: Plant cultivation, domestication and the spread of food production in tropical South America. *Quat. Sci. Rev.* **248**, 106582 (2020).

12. C. R. Clement, W. M. Denevan, M. J. Heckenberger, A. B. Junqueira, E. G. Neves, W. G. Teixeira, W. I. Woods, The domestication of Amazonia before European conquest. *Proc. R. Soc. B* **282**, 20150813 (2015).

13. C. Levis, F. R. C. Costa, F. Bongers, M. Peña-Claros, C. R. Clement, A. B. Junqueira, E. G. Neves, E. K. Tamanaha, F. O. G. Figueiredo, R. P. Salomão, C. V. Castilho, W. E. Magnusson, O. L. Phillips, J. E. Guevara, D. Sabatier, J.-F. Molino, D. C. López, A. M. Mendoza, N. C. A. Pitman, A. Duque, P. N. Vargas, C. E. Zartman, R. Vasquez, A. Andrade, J. L. Camargo, T. R. Feldpausch, S. G. W. Laurance, W. F. Laurance, T. J. Killeen, H. E. M. Nascimento, J. C. Montero, B. Mostacedo, I. L. Amaral, I. C. G. Vieira, R. Brienen, H. Castellanos, J. Terborgh, M. de Jesus Veiga Carim, J. R. da Silva Guimarães, L. de Souza Coelho, F. D. de Almeida Matos, F. Wittmann, H. F. Mogollón, G. Damasco, N. Dávila, R. García-Villacorta, E. N. H. Coronado, T. Emilio, D. Andrade Lima Filho, J. Schietti, P. Souza, N. Targhetta, J. A. Comiskey, B. S. Marimon, B.-H. Marimon, D. Neill, A. Alonso, L. Arroyo, F. A. Carvalho, F. C. de Souza, F. Dallmeier, M. P. Pansonato, J. F. Duivenvoorden, P. V. A. Fine, P. R. Stevenson, A. Araujo-Murakami, G. A. Aymard C, C. Baraloto, D. D. do Amaral, J. Engel, T. W. Henkel, P. Maas, P. Petronelli, J. D. C. Revilla, J. Stropp, D. Daly, R. Gribel, M. R. Paredes, M. Silveira, R. Thomas-Caesar, T. R. Baker, N. F. da Silva, L. V. Ferreira, C. A. Peres, M. R. Silman, C. Cerón, F. C. Valverde, A. Di Fiore, E. M. Jimenez, M.

C. P. Mora, M. Toledo, E. M. Barbosa, L. C. de Matos Bonates, N. C. Arboleda, E. de Sousa Farias, A. Fuentes, J.-L. Guillaumet, P. M. Jørgensen, Y. Malhi, I. P. de Andrade Miranda, J. F. Phillips, A. Prieto, A. Rudas, A. R. Ruschel, N. Silva, P. von Hildebrand, V. A. Vos, E. L. Zent, S. Zent, B. B. L. Cintra, M. T. Nascimento, A. A. Oliveira, H. Ramirez-Angulo, J. F. Ramos, G. Rivas, J. Schöngart, R. Sierra, M. Tirado, G. van der Heijden, E. V. Torre, O. Wang, K. R. Young, C. Baider, A. Cano, W. Farfan-Rios, C. Ferreira, B. Hoffman, C. Mendoza, I. Mesones, A. Torres-Lezama, M. N. U. Medina, T. R. van Andel, D. Villarroel, R. Zagt, M. N. Alexiades, H. Balslev, K. Garcia-Cabrera, T. Gonzales, L. Hernandez, I. Huamantupa-Chuquimaco, A. G. Manzatto, W. Milliken, W. P. Cuenca, S. Pansini, D. Pauletto, F. R. Arevalo, N. F. C. Reis, A. F. Sampaio, L. E. U. Giraldo, E. H. V. Sandoval, L. V. Gamarra, C. I. A. Vela, H. ter Steege, Persistent effects of pre-Columbian plant domestication on Amazonian forest composition. *Science* **355**, 925–931 (2017).

14. I. Wüst, C. Barreto, The ring villages of Central Brazil: A challenge for Amazonian archaeology. *Lat. Am. Antiq.* **10**, 3–23 (1999).

15. P. I. Schmitz, I. Wüst, S. M. Cope, U. Elfriede Thies, Arqueologia do Centro-Sul de Goiás. Uma fronteira de horticultores indígenas no Centro do Brasil. *Pesquisas* **33**, 1–281 (1982).

16. E. Robrahn Gonzalez, Os grupos ceramistas pré-coloniais do Centro-Oeste brasileiro. *Rev. Mus. Arqueol. Etnol.* **6**, 83–121 (1996).

17. J. Brochado, "An ecological model of the spread of pottery and agriculture into Eastern South America," thesis, University of Illinois, Urbana, IL (1984).

18. R. M. Bird, O. Dias Jr., E. de Carvalho, Subsídios para a arqueobotânica no Brasil: O milho antigo em cavernas de Minas Gerais, Brasil. *Rev. Arqueol.* **6**, 15–31 (1991).

19. D. M. Arruda, E. I. Fernandes-Filho, R. R. C. Solar, C. E. G. R. Schaefer, Combining climatic and soil properties better predicts covers of Brazilian biomes. *Sci. Nat.* **104**, 32 (2017).

20. N. Myers, R. A. Mittermeier, C. G. Mittermeier, G. A. B. da Fonseca, J. Kent, Biodiversity hotspots for conservation priorities. *Nature* **403**, 853–858 (2000).

21. M. J. Ferreira, C. Levis, L. Chaves, C. R. Clement, G. T. Soldati, Indigenous and traditional management creates and maintains the diversity of ecosystems of South American tropical savannas. *Front. Environ. Sci.* **10**, 809404 (2022).

22. P. da Conceição Bispo, M. C. A. Picoli, B. S. Marimon, B. H. Marimon Junior, C. A. Peres, I. O. Menor, D. E. Silva, F. de Figueiredo Machado, A. A. C. Alencar, C. A. de Almeida, L. O. Anderson, L. E. O. C. Aragão, F. M. Breunig, M. Bustamante, R. Dalagnol, J. A. F. Diniz-Filho, L. G. Ferreira, M. E. Ferreira, G. Fisch, L. S. Galvão, A. Giarolla, A. R. Gomes, P. de Marco Junior, T. N. Kuck, C. E. R. Lehmann, M. R. Lemes, V. Liesenberg, R. Loyola, M. N. Macedo, F. de Souza Mendes, S. do Couto de Miranda, D. C. Morton, Y. M. Moura, J. A. Oldekop, M. B. Ramos-Neto, T. M. Rosan, S. Saatchi, E. E. Sano, C. Segura-Garcia, J. Z. Shimbo, T. S. F. Silva, D. P. Trevisan, B. Zimbres, N. C. Wiederkehr, C. H. L. Silva-Junior, Overlooking vegetation loss outside forests imperils the Brazilian Cerrado and other non-forest biomes. *Nat. Ecol. Evol.* **8**, 12–13 (2024).

23. N. M. Stríkis, P. F. S. M. Buarque, F. W. Cruz, J. P. Bernal, M. Vuille, E. Tejedor, M. S. Santos, M. H. Shimizu, A. Ampuero, W. Du, G. Sampaio, H. dos Reis Sales, J. L. Campos, M. T. Kayano, J. Apaèstegui, R. R. Fu, H. Cheng, R. L. Edwards, V. C. Mayta, D. da Silva Francischini, M. A. Z. Arruda, V. F. Novello, Modern anthropogenic drought in Central Brazil unprecedented during last 700 years. *Nat. Commun.* **15**, 1728 (2024).

24. L. Bueno, A. Isnardis, Peopling Central Brazilian Plateau at the onset of the Holocene: Building territorial histories. *Quat. Int.* **473**, 144–160 (2018).

25. I. Wüst, Continuities and discontinuities: Archaeology and ethnoarchaeology in the heart of the Eastern Bororo territory, Mato Grosso, Brazil. *Antiquity* **72**, 663–675 (1998).

26. A. S. Barbosa, P. I. Schmitz, A. Stobäus, A. F. de Miranda, Projeto Médio-Tocantins: Monte Do Carmo, GO. Fase Cerâmica Pindorama. *Pesquisas* **34**, 49–92 (1982).

27. I. Wust, *Aspectos da ocupação pré-colonial em uma área do Mato Grosso de Goiás: Tentativa de análise espacial [1983]* (UNESC, Criciúma, 2019). [Aspects of pre-colonial occupation in an area of Mato Grosso de Goiás: Spatial analysis attempt].

28. J. Gregorio de Souza, J. A. Mateos, M. Madella, Archaeological expansions in tropical South America during the late Holocene: Assessing the role of demic diffusion. *PLOS ONE* **15**, e0232367 (2020).

29. P. I. Schmitz, M. B. Ribeiro, A. S. Barbosa, M. Barbosa, A. F. Miranda, *Caiapônia: Arqueologia nos Cerrados do Brasil Central* (Publicações Avulsas, 1986), vol. 8.

30. M. J. Heckenberger, "War and peace in the shadow of empire: Sociopolitical change in the Upper Xingu of Southeastern Amazonia, AD 1400-2000," thesis, University of Pittsburgh, Pittsburgh, PA (1996).

31. M. J. Heckenberger, A. Kuikuro, U. T. Kuikuro, J. C. Russell, M. Schmidt, C. Fausto, B. Franchetto, Amazonia 1492: Pristine forest or cultural parkland? *Science* **301**, 1710–1714 (2003).

32. M. J. DeNiro, Postmortem preservation and alteration of in vivo bone collagen isotope ratios in relation to palaeodietary reconstruction. *Nature* **317**, 806–809 (1985).

33. L. A. Martinelli, G. B. Nardoto, A. Soltangheisi, C. R. G. Reis, A. L. Abdalla-Filho, P. B. Camargo, T. F. Domingues, D. Faria, A. M. Figueira, T. F. Gomes, S. R. M. Lins, S. F. Mardegan, E. Mariano, R. C. Miatto, R. Moraes, M. Z. Moreira, R. S. Oliveira, J. P. H. B. Ometto, F. L. S. Santos, J. Sena-Souza, D. M. L. Silva, J. C. S. S. Silva, S. A. Vieira, Determining ecosystem functioning in Brazilian biomes through foliar carbon and nitrogen concentrations and stable isotope ratios. *Biogeochemistry* **154**, 405–423 (2021).

34. G. G. Gardiman, I. M. M. Rodrigues, L. M. Cascon, A. Isnardis, A morfologia dos vasos Jê na produção de cauim de milho em Vereda III: Uma proposição. *Rev. Mus. Arqueol. Etnol.* **27**, 111–120 (2016). [The morphology of Jê vessels in the production of maize cauim at Vereda III].

35. I. M. Rodrigues, "A ocupação do sítio arqueológico Vereda III," thesis, Universidade Federal de Minas Gerais, Belo Horizonte, MG (2011). [The occupation of the Vereda III archaeological site].

36. S. H. Ambrose, L. Norr, "Experimental evidence for the relationship of the carbon isotope ratios of whole diet and dietary protein to those of bone collagen and carbonate," in *Prehistoric Human Bone: Archaeology at Molecular Level* (Springer Berlin Heidelberg, ed. 1, 1993), pp. 1–37.

37. J. A. Lee-Thorp, J. C. Scaly, N. J. Van Der Merwe, Stable carbon isotope ratio differences between bone collagen and bone apatite, and their relationship to diet. *J. Archaeol. Sci.* **16**, 585–599 (1989).

38. T. Hermenegildo, "Dieta e padrões de subsistência das populações pré-históricas de caçadores-coletores do Brasil Central," thesis, Universidade de São Paulo, Piracicaba, SP (2009). [Diet and subsistence patterns of prehistoric hunter-gatherer populations of Central Brazil].

39. M. Q. R. Bastos, A. Solari, S. F. S. M. Da Silva, G. Martin, Estudo preliminar de dieta a partir de isótopos em grupos caçadores-coletores do agreste pernambucano (Holoceno recente-Nordeste do Brasil). *FUMDHAMentos* **16**, 3–18 (2019). [Preliminary dietary study based on isotopes from hunter-gatherer groups of the Pernambuco Agreste (Late Holocene, Northeastern Brazil)].

40. J. Steward, L. Faron, *Native Peoples of South America* (McGraw-Hill, ed. 1, 1959).

41. D. R. Gross, "A new approach to central Brazilian social organization," in *Brazil: Anthropological Perspectives - Essays in Honor of Charles Wagley*, M. L. Margolis, W. E. Carter, Eds. (Columbia Univ. Press, 1979), vol. 1, pp. 317–342.

42. A. Koch, C. Brierley, M. M. Maslin, S. L. Lewis, Earth system impacts of the European arrival and Great Dying in the Americas after 1492. *Quat. Sci. Rev.* **207**, 13–36 (2019).

43. M. Shock, R. Kipnis, L. Bueno, F. Silva, A chronology of the introduction of domesticated plants in central Brazil. *Tipití* **11**, 52–59 (2013).

44. E. Resende, J. Cardoso, Vestígios vegetais: Arqueobotânica e técnicas tradicionais de armazenamento. *Arq. Mus. Hist. Nat. Jard. Bot.* **19**, 229–258 (2009).

45. A. Strauss, R. E. Oliveira, X. S. Villagran, D. V. Bernardo, D. C. Salazar-García, M. C. Bissaro, F. Pugliese, T. Hermenegildo, R. Santos, A. Barioni, E. C. de Oliveira, J. C. Moreno de Sousa, K. Jaouen, M. Ernani, M. Hubbe, M. Inglez, M. Gratão, H. Rockwell, M. Machado, G. de Souza, F. Chemale, K. Kawashita, T. C. O'Connell, I. Israde, J. Feathers, C. Campi, M. Richards, J. Wahl, R. Kipnis, A. G. M. Araujo, W. Neves, Early Holocene ritual complexity in South America: The archaeological record of Lapa do Santo (east-central Brazil). *Antiquity* **90**, 1454–1473 (2016).

46. A. Roosevelt, Resource management in Amazonia before the conquest: Beyond ethnographic projection. *Adv. Econ. Bot.* **7**, 30–62 (1989).

47. T. Hermenegildo, H. Prümers, C. Jaimes Betancourt, P. Roberts, T. C. O'Connell, Stable isotope evidence for pre-colonial maize agriculture and animal management in the Bolivian Amazon. *Nat. Hum. Behav.* **9**, 464–471 (2025).

48. H. Prümers, C. J. Betancourt, J. Iriarte, M. Robinson, M. Schaich, Lidar reveals pre-Hispanic low-density urbanism in the Bolivian Amazon. *Nature* **606**, 325–328 (2022).

49. C. Jaimes-Betancourt, G. Fernandez, M. P. Shock, H. Nina, H. Delgadillo, G. Prestes-Carneiro, A. Lima, V. M. Nunes, R. Torrico, Historic landscapes, diversified livelihoods in the southwestern Amazon: The case of Lake Rogaguado and Lake Ginebra (Bolivia). *Front. Environ. Archaeol.* **4**, 1662950 (2025).

50. F. M. Costa, R. Vidal, N. C. de Almeida Silva, E. A. Veasey, F. de Oliveira Freitas, M. I. Zucchi, Archaeological findings show the extent of primitive characteristics of maize in South America. *Sci. Adv.* **10**, eadn1466 (2024).

51. R. E. M. Hedges, L. M. Reynard, Nitrogen isotopes and the trophic level of humans in archaeology. *J. Archaeol. Sci.* **34**, 1240–1251 (2007).

52. C. F. A. Moorrees, E. A. Fanning, E. E. Hunt Jr., Age variation of formation stages for ten permanent teeth. *J. Dent. Res.* **42**, 1490–1502 (1963).

53. R. Longin, New method of collagen extraction for radiocarbon dating. *Nature* **230**, 241–242 (1971).

54. A. Steinhof, M. Altenburg, H. Machts, Sample preparation at the Jena ^{14}C laboratory. *Radiocarbon* **59**, 815–830 (2017).

55. L. Wacker, G. Bonani, M. Friedrich, I. Hajdas, B. Kromer, M. Němec, M. Ruff, M. Suter, H.-A. Synal, C. Vockenhuber, MICADAS: Routine and high-precision radiocarbon dating. *Radiocarbon* **52**, 252–262 (2010).

56. A. Steinhof, Data analysis at the Jena ^{14}C laboratory. *Radiocarbon* **55**, 282–293 (2013).

57. M. Bleasdale, H.-P. Wotzka, B. Eichhorn, J. Mercader, A. Styring, J. Zech, M. Soto, J. Inwood, S. Clarke, S. Marzo, B. Fiedler, V. Linseele, N. Boivin, P. Roberts, Isotopic and microbotanical insights into Iron Age agricultural reliance in the Central African rainforest. *Commun. Biol.* **3**, 619 (2020).

58. A. Ventresca Miller, R. Fernandes, A. Janzen, A. Nayak, J. Swift, J. Zech, N. Boivin, P. Roberts, Sampling and pretreatment of tooth enamel carbonate for stable carbon and oxygen isotope analysis. *J. Vis. Exp.* **138**, e58002 (2018).

59. Posit team, *RStudio: Integrated Development. Environment for R*, version 2025.5.1.513 (Posit Software, PBC, 2025).

60. L. M. Müller, R. Kipnis, M. P. Ferreira, S. Marzo, B. Fiedler, M. Lucas, J. Ilgner, H. P. Silva, P. Roberts, Late Holocene dietary and cultural variability on the Xingu River, Amazon Basin: A stable isotopic approach. *PLOS ONE* **17**, e0271545 (2022).

61. T. Hermenegildo, T. C. O’Connell, V. L. C. Guapindaia, E. G. Neves, New evidence for subsistence strategies of late pre-colonial societies of the mouth of the Amazon based on carbon and nitrogen isotopic data. *Quat. Int.* **448**, 139–149 (2017).

62. A. Roosevelt, *Moundbuilders of the Amazon: Geophysical Archaeology on Marajo Island, Brazil* (Academic Press, 1991).

63. D. M. Arruda, C. E. G. R. Schaefer, R. S. Fonseca, R. R. C. Solar, E. I. Fernandes-Filho, Vegetation cover of Brazil in the last 21 ka: New insights into the Amazonian refugia and Pleistocenic arc hypotheses. *Glob. Ecol. Biogeogr.* **27**, 47–56 (2018).

64. J. Ratter, The Brazilian Cerrado vegetation and threats to its biodiversity. *Ann. Bot.* **80**, 223–230 (1997).

65. C. A. Klink, R. B. Machado, Conservation of the Brazilian Cerrado. *Conserv. Biol.* **19**, 707–713 (2005).

66. B. B. N. Strassburg, T. Brooks, R. Feltran-Barbieri, A. Iribarrem, R. Crouzeilles, R. Loyola, A. E. Latawiec, F. J. B. Oliveira Filho, C. A. de Mattos Scaramuzza, F. R. Scarano, B. Soares-Filho, A. Balmford, Moment of truth for the Cerrado hotspot. *Nat. Ecol. Evol.* **1**, 99 (2017).

67. J. Tomasella, A. P. M. A. Cunha, P. A. Simões, M. Zeri, Assessment of trends, variability and impacts of droughts across Brazil over the period 1980–2019. *Nat. Hazards* **116**, 2173–2190 (2023).

68. M. F. Moro, E. Nic Lughadha, F. S. de Araújo, F. R. Martins, A phytogeographical metaanalysis of the semiarid Caatinga domain in Brazil. *Bot. Rev.* **82**, 91–148 (2016).

69. N. M. Stríkis, F. W. Cruz, H. Cheng, I. Karmann, R. L. Edwards, M. Vuille, X. Wang, M. S. de Paula, V. F. Novello, A. S. Auler, Abrupt variations in South American monsoon rainfall during the Holocene based on a speleothem record from central-eastern Brazil. *Geology* **39**, 1075–1078 (2011).

70. M. Deininger, B. M. Ward, V. F. Novello, F. W. Cruz, Late quaternary variations in the South American monsoon system as inferred by speleothems—New perspectives using the SISAL database. *Quaternary* **2**, 6 (2019).

71. G. Utida, F. W. Cruz, R. V. Santos, A. O. Sawakuchi, H. Wang, L. C. R. Pessenda, V. F. Novello, M. Vuille, A. M. Strauss, A. C. Borella, N. M. Stríkis, C. C. F. Guedes, F. R. Dias De Andrade, H. Zhang, H. Cheng, R. L. Edwards, Climate changes in Northeastern Brazil

from deglacial to Meghalayan periods and related environmental impacts. *Quat. Sci. Rev.* **250**, 106655 (2020).

72. H. Cheng, A. Sinha, F. W. Cruz, X. Wang, R. L. Edwards, F. M. d'Horta, C. C. Ribas, M. Vuille, L. D. Stott, A. S. Auler, Climate change patterns in Amazonia and biodiversity. *Nat. Commun.* **4**, 1411 (2013).

73. S. Mendonça de Souza, L. Sianto, A. Fernandes, A. M. Jansen, A. C. Vicente, R. Kipnis, L. F. Ferreira, K. Dittmar, A. Araujo, Sepultamento IV do sítio arqueológico Lapa do Boquete, MG: Patologias ósseas, parasitoses e doença de Chagas. *Arq. Mus. Hist. Nat. Jard. Bot.* **19**, 207–228 (2009). [Burial IV from the Lapa do Boquete archaeological site, Minas Gerais: Bone pathologies, parasitic infections, and Chagas disease].

74. A. Prous, *Arqueologia Brasileira* (Editora Universidade de Brasília, ed. 1, 1992). [Brazilian Archaeology].

75. P. I. Schmitz, A. S. Barbosa, *Horticultores Pré-Históricos do Estado de Goiás* (Instituto Anchietano de Pesquisas, 1985). [Prehistoric Horticulturalists of the State of Goiás].

76. P. I. Schmitz, A. S. Barbosa, A. Jacobus, M. Barbieri, Arqueologia nos cerrados do Brasil Central: Serranópolis I. *Pesquisas* **44**, 1–208 (1989). [Archaeology in the Cerrados of Central Brazil: Serranópolis I].

77. F. de Oliveria Freitas, Influência cultural-alimentar das populações pré-históricas do vale do rio Peruaçu. Estudo de caso - milho (*Zea mays mays*) e feijão (*Phaseolus vulgaris*). *Arq. Mus. Hist. Nat. Jard. Bot.* **19**, 259–274 (2009). [Dietary-cultural influence of the prehistoric populations of the Peruaçu River Valley: A case study of maize (Zea mays mays) e common bean (Phaseolus vulgaris)].

78. A. Prous, Artefatos de cerâmica, argila, osso, chifre, dente, vegetal e concha. *Arq. Mus. Hist. Nat. Jard. Bot.* **12**, 171–178 (1991). [Artifacts of ceramic, clay, bone, antler, tooth, plant, and shell].

79. A. Prous, Artefatos e adornos sobre suportes de origem animal, vegetal ou mineral (concha, casca de ovo, dente, osso, cera, fibras vegetais e calcita). *Arq. Mus. Hist. Nat. Jard. Bot.* **19**, 371–413 (2009). [Artifacts and ornaments made from animal, plant, or mineral raw materials (shell, eggshell, tooth, bone, wax, plant fibers, and calcite)].

80. E. Resende, A. Prous, Os vestígios vegetais do Grande Abrigo de Santana do Riacho. *Arq. Mus. Hist. Nat. Jard. Bot.* **12**, 87–111 (1991). [Plant remains from the Grande Abrigo de Santana do Riacho].

81. A. Prous, M. C. Schlobach, Sepultamentos pré-históricos do Vale do Peruaçu - MG. *Rev. Mus. Arqueol. Etnol.* **7**, 3–21 (1997). [Prehistoric burials from the Peruaçu Valley].

82. G. A. M. Sene, "Indicadores de gênero na pré-história brasileira: Contexto funerário, simbolismo e diferenciação social. O sítio arqueológico Gruta do Gentio II, Unaí, Minas Gerais," thesis, Universidade de São Paulo, São Paulo, SP (2007). [Gender indicators in Brazilian prehistory: Funerary context, symbolism, and social differentiation. The Gruta do Gentio II archaeological site, Unaí, Minas Gerais].

83. A. Strauss, I. M. Mariano Rodrigues, A. Baeta, X. S. Villagran, M. Alves, F. Pugliese, M. Bissaro, R. E. de Oliveira, G. N. de Souza, L. Bueno, J. C. M. de Sousa, J. J. Morrow, K. J. Reinhard, T. Hermenegildo, G. C. Perez, E. N. Chim, R. de Oliveira dos Santos, M. de Paiva, R. Kipnis, W. Neves, "The archaeological record of Lagoa Santa (East-Central Brazil): From the Late Pleistocene to Historical Times," in *Lagoa Santa Karst: Brazil's Iconic Karst Region*, A. Auler, P. Pessoa, Eds. (Springer, 2020).

84. H. L. Fernandes, "Os sepultamentos do sítio Aratu de Piragiba–BA," thesis, Universidade Federal da Bahia, Salvador, BA (2003). [The burials from the Aratu site of Piragiba, Bahia].

85. C. Barreto, A Construção Social do Espaço: De volta às aldeias circulares do Brasil Central. *Rev. Habitus* **9**, 61–80 (2012). [The Social Construction of Space: Back to the Circular Villages of Central Brazil].

86. A. Nikulin, "Proto-Macro-Jê: Um estudo reconstrutivo," thesis, Universidade de Brasília, Brasília, DF (2020). [Proto-Macro-Jê: A reconstructive study].

87. C. Nimuendajú, *Mapa Etno-Histórico Do Brasil e Regiões Adjacentes [1944]* (IPHAN, IBGE, ed. 2, 2017). [Ethno-Historical Map of Brazil and Adjacent Regions].

88. V. Ramallo, R. Bisso-Machado, C. Bravi, M. D. Coble, F. M. Salzano, T. Hünemeier, M. C. Bortolini, Demographic expansions in South America: Enlightening a complex scenario with genetic and linguistic data. *Am. J. Phys. Anthropol.* **150**, 453–463 (2013).

89. T. Ferraz, X. Suarez Villagran, K. Nägele, R. Radzevičiūtė, R. Barbosa Lemes, D. C. Salazar-García, V. Wesolowski, M. Lopes Alves, M. Bastos, A. Rapp Py-Daniel, H. Pinto Lima, J. Mendes Cardoso, R. Estevam, A. Liryo, G. M. Guimarães, L. Figuti, S. Eggers, C. R. Plens, D. M. Azevedo Erler, H. A. Valadares Costa, I. da Silva Erler, E. Koole, G. Henriques, A. Solari, G. Martin, S. F. Serafim Monteiro da Silva, R. Kipnis, L. M. Müller, M. Ferreira, J. Carvalho Resende, E. Chim, C. A. da Silva, A. C. Borella, T. Tomé, L. Müller Plumm Gomes, D. Barros Fonseca, C. Santos da Rosa, J. D. de Moura Saldanha, L. Costa Leite, C. M. S. Cunha, S. A. Viana, F. Ozorio Almeida, D. Klokler, H. L. A. Fernandes, S. Talamo, P. DeBlasis, S. Mendonça de Souza, C. de Paula Moraes, R. E. Oliveira, T. Hünemeier, A. Strauss, C. Posth, Genomic history of coastal societies from eastern South America. *Nat. Ecol. Evol.* **7**, 1315–1330 (2023).

90. E. R. Ribeiro, Tapuya connections: Language contact in eastern Brazil. *LIAMES* **9**, 61–76 (2010).

91. E. Koole, "Entre as tradições planálticas e meridionais: Caracterização arqueológica dos grupos caçadores coletores a partir da análise de sete elementos e suas implicações para a ocupação pré-cerâmica da Região Cárstica do Alto São Francisco, Minas Gerais, Brasil," thesis, Universidade de São Paulo, São Paulo, SP (2014). [Archaeological characterization of hunter-gatherer groups based on the analysis of seven elements and its implications for the pre-ceramic occupation of the karst region of the Upper São Francisco, Minas Gerais, Brazil].

92. D. C. Martins, M. E. Borges, S. F. Silva, S. C. Piedade, O homem do Rio das Almas: Remanescentes esqueletais humanos do GO-Ni.217, Sítio Abrigo Tuvira, município de Barro Alto, Estado de Goiás. *Rev. Mus. Arqueol. Etnol.* **12**, 55–70 (2002). [The man from Rio das Almas: Human skeletal remains from GO-Ni.217, Tuvira Rock Shelter site, Barro Alto, Goiás].

93. IPHAN, *Cadastro Nacional de Sítios Arqueológicos (CNSA)* (IPHAN, 2018). [National Register of Archaeological Sites (CNSA)].

94. D. Martins, A Arqueologia da Serra da Mesa. *Rev. Mus. Antropol.* **8**, 85–118 (2005). [The Archaeology of Serra da Mesa].

95. M. A. Alves, L. Cheuiche, Estruturas Arqueológicas e Padrões de Sepultamento do Sítio de Água Limpa, Município de Monte Alto, São Paulo, in *Coleção Arqueologia* (EDIPUCRS, 1995), vol. 1, pp. 295–310. [Archaeological Structures and Burial Patterns at the Água Limpa Site, Municipality of Monte Alto, São Paulo, in Archaeology Collection].

96. C. Etchevarne, L. A. Fernandes, "Urnas funerárias encontradas em Água Vermelha, Reserva Indígena Caramuru-Paraguassu (BA): Informe acerca dos procedimentos adotados no Laboratório de Arqueologia da Faculdade de Filosofia e Ciências Humanas da Universidade Federal da Bahia" (2011). [Funerary urns found in Água Vermelha, Caramuru-Paraguaçu Indigenous Reserve (Bahia)].

97. O. A. de Carvalho, C. Etchevarne, A. N. de Queiroz, Associação de vasos cerâmicos e ossos de animais: Ritual funerário ou resto de cozinha em populações do passado provenientes da região nordeste do Brasil? *Rev. Etnobiol.* **17**, 76–88 (2019). [Association between ceramic vessels and animal bones: Funerary ritual or kitchen waste in past populations from Northeastern Brazil?].

98. M. Andreatta, Projeto Anhanguera de Arqueologia de Goiás: 1975-1985. *Rev. Mus. Paul.* **33**, 275–282 (1988). [Anhanguera Archaeology Project of Goiás: 1975-1985].

99. M. Andreatta, "Padrões de povoamento em pré-história goiana: Análise de sítio tipo," thesis, Universidade de São Paulo, São Paulo, SP (1982). [Settlement patterns in Goiás prehistory: Analysis of type site].

100. I. Wüst, "Salvamento de uma urna com restos osteológicos da fase Mossâmedes no município de Sanclerlândia, Goiás" (Goiânia, 1992). [Rescue of an urn with osteological remains from the Mossâmedes phase in the municipality of Sanclerlândia, Goiás].

101. T. B. Oliveira, J. de Souza Santos, “Abrigos rochosos e sepultamentos indígenas no interior da Paraíba, Brasil,” in *Anais do 34° Congresso Brasileiro de Espeleologia*, M. Rasteiro, C. Teixeira-Silva, S. Lacerda, Eds. (SBE, 2017), pp. 587–593. [Rock shelters and Indigenous burials in the interior of Paraíba, Brazil].

102. L. Meneses, A. P. M. de Araújo, F. A. de Aguiar Moraes, Inventário de sítios pré-históricos na área do projeto Geoparque Cariri Paraibano, Brasil. *GEOgraphia* **24**, 1–23 (2022). [Inventory of prehistoric sites in the area of the Cariri Paraibano Geopark project, Brazil].

103. J. de Souza Santos, A. A. de Farias, Diagênese óssea nos cemitérios indígenas dos Sertões da Paraíba. *Clio Arqueol.* **24**, 111–125 (2009). [Bone diagenesis in Indigenous cemeteries of the Sertões of Paraíba].

104. J. C. R. de Rubin, R. T. da Silva, D. S. Correa, S. M. da Silva, J. B. Barbosa, “Nueva perspectiva del abrigo del sítio arqueológico GO-CP-04, Goiás, Brasil,” in *El Poblamiento Temprano en América*, F. A. Arellano, E. G. S. Bautista, Eds. (José Concepción Jiménez López, 2016), pp. 195–210. [A new perspective on the GO-CP-04 rock shelter site, Goiás, Brazil,” in Early Settlement in the Americas].

105. S. A. Viana, M. P. Ramos, C. L. Dantas, E. Boeda, J. C. Rubin, S. A. Viana, E. Yokoyama, C. Wichers, M. Barbieri, E. Nunes, G. Procópio, W. Vaz-Silva, N. Mundin, “As sutilezas do registro arqueológico de Palestina de Goiás, Brasil,” in *Arqueologia no Centro-Oeste do Brasil: Cultura, Ciência e Política*, D. T. Mendes, R. L. Souza, Eds. (Cegraf UFG, 2025), pp. 208–259. [The subtleties of the archaeological record of Palestina de Goiás, Brazil].

106. J. L. Couto, S. A. Viana, E. T. de Souza, Da produção ao funcionamento: Uma abordagem experimental sobre “lesmas” líticas do tecno-complexo Itaparica de Serranópolis, Brasil. *Rev. Habitus* **23**, 25–41 (2025). [From production to use: An experimental approach to lithic “limaces” from the Itaparica techno-complex of Serranópolis, Brazil].

107. S. A. Viana, J. C. Rubin, S. E. Hoeltz, M. Barberi, K. G. de Oliveira, F. Oliveira, Techno-cultural singularities in the southwestern region of the Brazilian central plateau in the early Holocene. *J. Lithic Stud.* **11**, 1–36 (2024).

108. P. I. Schmitz, A. O. Rosa, A. L. V. Bittencourt, Arqueologia nos cerrados do Brasil central: Serranópolis III. *Pesquisas* **60**, 3–286 (2004).

109. P. I. Schmitz, A evolução da cultura no sudoeste de Goiás. *Pesquisas* **30**, 185–225 (1980). [The evolution of culture in southwestern Goiás].

110. P. I. Schmitz, Caçadores Antigos no Sudoeste de Goiás, Brasil. *Estud. Atacam.* **8**, 16–35 (1987). [Ancient Hunters in Southwestern Goiás, Brazil].

111. A. Prous, Fouilles de l'abri du Boquete, Minas Gerais, Brésil. *J. Soc. Am.* **77**, 77–109 (1991). [Excavations at the Boquete rock shelter, Minas Gerais, Brazil].

112. L. D. Romualdo da Silva, M. Okumura, Cestos enterrados no Vale do Peruaçu: Classificação e utilização dos artefatos têxteis e trançados dos sítios sob abrigo do norte de Minas Gerais. *Rev. Arqueol.* **31**, 131–150 (2018). [Buried baskets in the Peruaçu Valley: Classification and use of textile artifacts from rock shelter sites in northern Minas Gerais].

113. R. Kipnis, "Foraging societies of Eastern Central Brazil: An evolutionary ecological study of subsistence strategy," thesis, University of Michigan, Ann Arbor, MI (2002).

114. A. Isnardis, V. Linke, De estruturas a corpos e seres: Os vestígios perecíveis da Lapa do Caboclo em Diamantina, Minas Gerais. *Rev. Arqueol.* **34**, 122–145 (2021). [From structures to bodies and beings: The perishable remains of Lapa do Caboclo in Diamantina, Minas Gerais].

115. A. Solari, A. Isnardis, V. Linke, Entre Cascas e Couros: Os sepultamentos secundários da Lapa do Caboclo (Diamantina, Minas Gerais). *Rev. Habitus* **10**, 115–134 (2012). [Between bark and leather: The secondary burials of Lapa do Caboclo (Diamantina, Minas Gerais)].

116. P. A. Junqueira, Pinturas e gravações rupestres das Lapas Pequena e Pintada, município de Montes Claros, Minas Gerais. *Arq. Mus. Hist. Nat. Jard. Bot* **3**, 327–341 (1978). [Rock paintings and engravings from Lapa Pequena and Lapa Pintada, municipality of Montes Claros, Minas Gerais].

117. L. Bueno, Tecnologia e Território no Centro-Norte mineiro: Um estudo de caso na região de Montes Claros, MG, Brasil. *Rev. Espinhaço* **2**, 168–186 (2013). [Technology and Territory in north-central Minas Gerais: A case study in the Montes Claros region, Minas Gerais, Brazil].

118. J. L. A. Silva, J. A. C. Almeida, Reflexões arqueológicas: Estudo dos sítios arqueológicos do município de Queimadas/PB. *Tarairiú* **1**, 112–126 (2011). [Study of the archaeological sites in the municipality of Queimadas, Paraíba].

119. P. J. Mello, "Programa de Preservação do Patrimônio Arqueológico do AHE Serra do Facão - Relatório Final" (Goiânia, 2011). [Archaeological Heritage Preservation Program of the Serra do Facão Hydroelectric Project (AHE) – Final Report].

120. J. de Souza Santos, "Práticas funerárias e cultura material nos Sertões da Paraíba: A necrópole sítio Pinturas I, em São João do Tigre," thesis, Universidade Federal de Pernambuco, Recife, PE (2009). [Funerary practices and material culture in the Sertões of Paraíba: The Pinturas I site necropolis in São João do Tigre].

121. C. Etchevarne, A ocupação humana do Nordeste brasileiro antes da colonização portuguesa. *Rev. USP* **44**, 112–141 (1999). [Human occupation of Northeastern Brazil before Portuguese colonization].

122. A. F. F. Lopes, J. de S. Santos, Cavidades naturais como espaços de sepultamentos indígenas Cariri: O caso do sítio Serrote dos Ossos, Caraúbas, Paraíba. *Tarairiú* **1**, 1–13 (2023). [Natural caves as burial spaces: The case of the Serrote dos Ossos site, Caraúbas, Paraíba].

123. T. S. Cavalcante, M. V. da Silva Limeira, J. de Souza Santos, O sítio arqueológico Serrote dos Ossos, Caraúbas – PB. *Tarairiú* **1**, 1–16 (2023). [The Serrote dos Ossos archaeological site, Caraúbas, Paraíba].

124. M. de O. Barbosa, P. J. C. Mello, S. A. Viana, "Relatório Parcial do Resgate Arqueológico do Sítio Vale dos Sonhos, Goiânia-GO" (Goiânia, 2002). [Excavations at the Vale dos Sonhos site, Goiânia, Goiás].

125. M. de O. Barbosa, P. J. C. Mello, "Relatório de Levantamento do Patrimônio Arqueológico da Área Diretamente Afetada pela Implantação do Projeto Urbanístico da Etapa II do Residencial Vale dos Sonhos, Goiânia - GO" (Goiânia, 2004). [Archaeological Heritage Survey of the Vale dos Sonhos Residential Area, Goiânia, Goiás].

126. H. L. A. Fernandes, "Sítio Vale Verde 1: Escavações emergenciais no município de Sítio do Mato - Bahia" (Cachoeira, BA, 2011). [Vale Verde 1 Site: Excavations in the municipality of Sítio do Mato, Bahia].

127. J. C. Resende, "Estudos bioarqueológicos dos sepultamentos do sítio cerâmico Vau 1, Bahia," thesis, PUC-GO, Goiânia, GO (2021). [Bioarchaeological studies of the burials from the Vau 1 site, Bahia].

128. P. Roberts, N. Perera, O. Wedage, S. Deraniyagala, J. Perera, S. Eregama, M. D. Petraglia, J. A. Lee-Thorp, Fruits of the forest: Human stable isotope ecology and rainforest adaptations in Late Pleistocene and Holocene (~36 to 3 ka) Sri Lanka. *J. Hum. Evol.* **106**, 102–118 (2017).

129. N. J. van der Merwe, E. Medina, The canopy effect, carbon isotope ratios and foodwebs in amazonia. *J. Archaeol. Sci.* **18**, 249–259 (1991).

130. M. J. Schoeninger, M. J. DeNiro, Nitrogen and carbon isotopic composition of bone collagen from marine and terrestrial animals. *Geochim. Cosmochim. Acta* **48**, 625–639 (1984).

131. B. N. Smith, S. Epstein, Two categories of $^{13}C/^{12}C$ ratios for higher plants. *Plant Physiol.* **47**, 380–384 (1971).

132. M. H. O'Leary, Carbon isotope fractionation in plants. *Phytochemistry* **20**, 553–567 (1981).

133. J. V. Tejada, J. J. Flynn, P.-O. Antoine, V. Pacheco, R. Salas-Gismondi, T. E. Cerling, Comparative isotope ecology of western Amazonian rainforest mammals. *Proc. Natl. Acad. Sci. U.S.A.* **117**, 26263–26272 (2020).

134. M. Sponheimer, J. A. Lee-Thorp, Oxygen isotopes in enamel carbonate and their ecological significance. *J. Archaeol. Sci.* **26**, 723–728 (1999).

135. W. Dansgaard, Stable isotopes in precipitation. *Tellus* **16**, 436–468 (1964).

136. J. R. Gat, Oxygen and hydrogen isotopes in the hydrologic cycle. *Annu. Rev. Earth Planet. Sci.* **24**, 225–262 (1996).

137. B. L. Turner, V. Bélisle, A. R. Davis, M. Skidmore, S. L. Juengst, B. J. Schaefer, R. A. Covey, B. S. Bauer, Diet and foodways across five millennia in the Cusco region of Peru. *J. Archaeol. Sci.* **98**, 137–148 (2018).

138. P. Roberts, D. Gaffney, J. Lee-Thorp, G. Summerhayes, Persistent tropical foraging in the highlands of terminal Pleistocene/Holocene New Guinea. *Nat. Ecol. Evol.* **1**, 44 (2017).

139. A. Willis, P. Roberts, M. M. Kyaw, T. T. Win, B. Pradier, C. King, J. Ilgner, M. Lucas, A. Cook, P. Piper, T. O. Pryce, Living in the dry zone: Stable isotope insights into palaeodiet in ancient Myanmar. *J. Archaeol. Sci. Rep.* **48**, 103900 (2023).

140. M. A. De Masi, "Prehistoric hunter-gatherer mobility on the southern Brazilian coast: Santa Catarina Island," thesis, Stanford University, Stanford, CA (1999).

141. N. J. van der Merwe, A. C. Roosevelt, J. C. Vogel, Isotopic evidence for prehistoric subsistence change at Parmana, Venezuela. *Nature* **292**, 536–538 (1981).

142. A. C. Colonese, R. Winter, R. Brandi, T. Fossile, R. Fernandes, S. Soncin, K. McGrath, M. Von Tersch, A. M. Bandeira, Stable isotope evidence for dietary diversification in the pre-Columbian Amazon. *Sci. Rep.* **10**, 16560 (2020).

143. L. Hilbert, E. G. Neves, F. Pugliese, B. S. Whitney, M. Shock, E. Veasey, C. A. Zimpel, J. Iriarte, Evidence for mid-Holocene rice domestication in the Americas. *Nat. Ecol. Evol.* **1**, 1693–1698 (2017).

144. U. Lombardo, J. Iriarte, L. Hilbert, J. Ruiz-Pérez, J. M. Capriles, H. Veit, Early Holocene crop cultivation and landscape modification in Amazonia. *Nature* **581**, 190–193 (2020).

145. A. C. Colonese, M. Collins, A. Lucquin, M. Eustace, Y. Hancock, R. de Almeida Rocha Ponzoni, A. Mora, C. Smith, P. DeBlasis, L. Figuti, V. Wesolowski, C. R. Plens, S. Eggers,

D. S. E. de Farias, A. Gledhill, O. E. Craig, Long-term resilience of late holocene coastal subsistence system in Southeastern South America. *PLOS ONE* **9**, e93854 (2014).

146. L. Pezo-Lanfranco, S. Eggers, C. Petronilho, A. Toso, D. da Rocha Bandeira, M. Von Tersch, A. M. P. dos Santos, B. Ramos da Costa, R. Meyer, A. C. Colonese, Middle Holocene plant cultivation on the Atlantic Forest coast of Brazil? *R. Soc. Open Sci.* **5**, 180432 (2018).

147. M. A. De Masi, Aplicações de isótopos estáveis de O, C e N em estudos de sazonalidade, mobilidade e dieta de populações pré-históricas no sul do Brasil. *Rev. Arqueol.* **22**, 55–76 (2009). [Applications of stable isotopes of O, C, and N in studies of seasonality, mobility, and diet of prehistoric populations in southern Brazil].

148. A. Toso, E. Hallingstad, K. McGrath, T. Fossile, C. Conlan, J. Ferreira, D. da Rocha Bandeira, P. C. F. Giannini, S.-P. Gilson, L. de Melo Reis Bueno, M. Q. R. Bastos, F. M. Borba, A. M. P. do Santos, A. C. Colonese, Fishing intensification as response to Late Holocene socio-ecological instability in southeastern South America. *Sci. Rep.* **11**, 23506 (2021).

149. J. Mendes Cardoso, F. Merencio, X. Villagran, V. Wesolowski, R. Estevam, B. T. Fuller, P. DeBlasis, S. Pierre-Gilson, D. Guiserix, P. Méjean, L. Figuti, D. Farias, G. Guimaraes, A. Strauss, K. Jaouen, Late shellmound occupation in southern Brazil: A multi-proxy study of the Galheta IV archaeological site. *PLOS ONE* **19**, e0300684 (2024).

150. V. Wesolowski, S. M. F. M. de Souza, K. Reinhard, G. Ceccantini, Grânulos de amido e fitólitos em cálculos dentários humanos: Contribuição ao estudo do modo de vida e subsistência de grupos sambaquianos do litoral sul do Brasil. *Rev. Mus. Arqueol. Etnol.* , 191–210 (2007). [Starch and phytoliths in human dental calculus: Contribution to the study of lifestyle and subsistence of sambaqui groups from the southern Brazilian coast].

151. V. Wesolowski, S. M. Ferraz Mendonça de Souza, K. J. Reinhard, G. Ceccantini, Evaluating microfossil content of dental calculus from Brazilian sambaquis. *J. Archaeol. Sci.* **37**, 1326–1338 (2010).

152. M. Q. R. Bastos, V. Guida, C. Rodrigues-Carvalho, A. Toso, R. V. Santos, A. Colonese, Elucidating pre-columbian tropical coastal adaptation through bone collagen stable isotope analysis and bayesian mixing models: Insights from Sambaqui do Moa (Brazil). *Rev. Antropol. Mus. Entre Ríos* **7**, 1–10 (2022).

153. G. Oppitz, M. Q. R. Bastos, L. Z. Scherer, A. Lessa, V. Martins, P. Camargo, P. DeBlasis, Pensando sobre mobilidade, dieta e mudança cultural: Análises isotópicas no sítio Armação do Sul, Florianópolis/SC. *Cad. Lepaarq* **15**, 237–266 (2018). [Mobility, diet, and cultural change: Isotopic analyses at the Armação do Sul site, Florianópolis, Santa Catarina].

154. M. Q. R. Bastos, A. Lessa, C. Rodrigues-Carvalho, R. H. Tykot, R. V. Santos, Análise de isótopos de carbono e nitrogênio: A dieta antes e após a presença de cerâmica no sítio Forte Marechal Luz. *Rev. Mus. Arqueol. Etnol.* **24**, 137–151 (2014). [Carbon and nitrogen isotope analysis: Diet before and after the presence of ceramics at the Forte Marechal Luz site].
